# Mitochondrial Biogenesis in Diverse Cauliflower Cultivars under Mild and Severe Drought. Impaired Coordination of Selected Transcript and Proteomic Responses, and Regulation of Various Multifunctional Proteins

**DOI:** 10.3390/ijms19041130

**Published:** 2018-04-10

**Authors:** Michał Rurek, Magdalena Czołpińska, Tomasz Andrzej Pawłowski, Aleksandra Maria Staszak, Witold Nowak, Włodzimierz Krzesiński, Tomasz Spiżewski

**Affiliations:** 1 Department of Molecular and Cellular Biology, Institute of Molecular Biology and Biotechnology, Adam Mickiewicz University, Poznań, Umultowska 89, 61-614 Poznań, Poland; magczo@amu.edu.pl; 2 Institute of Dendrology, Polish Academy of Sciences, Parkowa 5, 62-035 Kórnik, Poland; tapawlow@man.poznan.pl (T.A.P.); a.staszak@uwb.edu.pl (A.M.S.); 3 Present address: Department of Plant Physiology, Institute of Biology, Faculty of Biology and Chemistry, University of Białystok, Ciołkowskiego 1J, 15-245 Białystok, Poland; 4 Molecular Biology Techniques Laboratory, Faculty of Biology, Adam Mickiewicz University, Poznań, Umultowska 89, 61-614 Poznań, Poland; nowak@amu.edu.pl; 5 Department of Vegetable Crops, Poznan University of Life Sciences, Dąbrowskiego 159, 60-594 Poznań, Poland; wlodzimierz.krzesinski@up.poznan.pl (W.K.); tomasz.spizewski@up.poznan.pl (T.S.)

**Keywords:** dehydrins, 2D PAGE, drought, mitochondrial biogenesis, mitochondrial proteome, plant transcriptome

## Abstract

Mitochondrial responses under drought within *Brassica* genus are poorly understood. The main goal of this study was to investigate mitochondrial biogenesis of three cauliflower (*Brassica oleracea* var. *botrytis*) cultivars with varying drought tolerance. Diverse quantitative changes (decreases in abundance mostly) in the mitochondrial proteome were assessed by two-dimensional gel electrophoresis (2D PAGE) coupled with liquid chromatography-tandem mass spectrometry (LC-MS/MS). Respiratory (e.g., complex II, IV (CII, CIV) and ATP synthase subunits), transporter (including diverse porin isoforms) and matrix multifunctional proteins (e.g., components of RNA editing machinery) were diversely affected in their abundance under two drought levels. Western immunoassays showed additional cultivar-specific responses of selected mitochondrial proteins. Dehydrin-related tryptic peptides (found in several 2D spots) immunopositive with dehydrin-specific antisera highlighted the relevance of mitochondrial dehydrin-like proteins for the drought response. The abundance of selected mRNAs participating in drought response was also determined. We conclude that mitochondrial biogenesis was strongly, but diversely affected in various cauliflower cultivars, and associated with drought tolerance at the proteomic and functional levels. However, discussed alternative oxidase (AOX) regulation at the RNA and protein level were largely uncoordinated due to the altered availability of transcripts for translation, mRNA/ribosome interactions, and/or miRNA impact on transcript abundance and translation.

## 1. Introduction

Under drought, plants respond through numerous physiological and molecular mechanisms [1]. Each plant species possesses a unique drought resistance response, which is accompanied by the diverse sensitivity of selected growth and metabolic processes to progressing stress conditions [2]. The balance between water uptake and transpiration is controlled by water potential. The leaf surface controls CO_2_ assimilation as well as photosynthetic and respiration rates. Excessive transpiration may decrease the water potential in plants, resulting in growth cessation [3]. Initially, drought results in stomatal closure and declined transpiration to prevent further water losses among drought-sensitive species, in osmolyte synthesis, and consequently, leaf cell growth inhibition. Drought regulates leaf respiration in various directions, nevertheless, those alterations may enable prompt stress recovery [4,5,6,7,8]. 

The mitochondrial proteome is a highly dynamic entity containing at least 1500 diverse proteins (1060 of which have been identified in potato (*Solanum tuberosum*) mitochondria by Salvato et al. [9]) actively responding to environmental conditions. Over the past 15 years, significant progress has been made on the elucidation of key steps of mitochondrial biogenesis, which implies finely coordinated expression of mitochondrial and nuclear genes that can be disrupted under stress action [10,11,12,13]. Taylor et al. [14] estimated 22% of the stress-responsive organellar proteins in *Arabidopsis* to be targeted to mitochondria, but the number of mitochondrial proteins involved in diverse stress response still remains underestimated. More complex studies integrating various approaches for better understanding of drought responses are required. 

Drought also results in dynamic alterations within the cellular transcriptome and proteome [7,15,16,17], including the mitochondrial proteome [18,19]. Drought response is often connected with the increase or induction of diverse protective proteins, such as dehydrins [20]. Mitochondrial proteins (including key enzymes) may be directly involved in developing of drought tolerance as well; under drought, some novel protein isoforms may be also induced, although the proteolysis of some mitochondrial proteins was also reported [19,21,22,23,24,25,26,27]. Variations in the abundance of numerous mitochondrial proteins, however, may not clearly correspond with the drought intensity. 

*Brassica* genus contains important plant species for worldwide agriculture. Total cellular proteomic and transcriptomic responses of *Brassica* species in drought have already been investigated, although without deepened attention towards elucidation of the particular aspects of mitochondrial biogenesis [28,29,30,31,32]. Interestingly, drought response of close *Brassica* relatives, including *Thellungiella*, are distinct from that observed for *Arabidopsis* [33]. However, reports comparing responses of *Brassica* cultivars with contrasting drought tolerance are still limited [34,35,36,37,38], contrary to other species data [6,39,40,41,42,43,44,45,46,47,48]. Search for protein markers in order to develop drought-tolerant plant accessions belongs to the current goals of proteomic analyses [7]. 

Owing to recent research trends, this work was undertaken to gain a comprehensive view of the influence of middle and severe water deficiency conditions on the mitochondrial biogenesis of three cauliflower (*Brassica oleracea* var. *botrytis*) cultivars displaying diverse drought tolerance. Previously, we studied cauliflower mitochondrial biogenesis under temperature stress and subsequent recovery [13]. Cauliflower belongs to vegetables with the major cultivation yield in Central Europe. The early generative phase of curd ripening belongs to the key developmental stages with some physiological demands. In addition, due to the size of its vegetative organs, it is sensitive to the low water level in the soil. To determine mitochondrial responses in relation to plant respiration, we aimed (1) to investigate the dynamic nature of the mitochondrial proteome; (2) to identify the most variable proteins in cauliflower curd mitochondria; (3) to determine the abundance of selected mitochondrial proteins, including dehydrin-like proteins previously investigated by us [49]; (4) to analyze relevant proteomic and transcriptomic alterations; and (5) to link them with the physiological level (respiratory and photorespiratory alterations) for the discussion of the general responses to mild and severe water deficit. This is the first comprehensive study of the mitochondrial proteome of the *Brassica* genus member which allowed characterization of a broadened set of drought-responsive mitochondrial proteins in the cultivar context. It highlights the participation not only of oxidative phosphorylation (OXPHOS) proteins, but a number of multifunctional mitochondrial proteins (including RNA editing factors and dehydrin-like proteins) in drought response.

## 2. Results 

### 2.1. Respiration and Photorespiration Pattern in Cauliflower Leaves

In order to study mitochondrial response at physiological and molecular levels in “*Adelanto*” (“*A*”) and “*Casper*” (“*C*”) drought-sensitive cultivars, as well as in the “*Pionier*” (“*P*”) drought-tolerant cultivar, total light (R_T_), day (R_d_), and night (R_n_) respiration, as well as the photorespiration (PhR) rates were determined. 

Generally, R_T_ slightly increased under mild drought, but markedly decreased in severe treatment in “*A*” and especially in “*C*”. In “*P*”, R_T_ rate only slightly decreased under severe drought (Figure S1). R_d_ (the average from all illumination conditions), as well as R_n_ increased progressively in the drought-sensitive cultivars under all investigated treatments. R_d_ rate was markedly decreased in “*P*” in mild drought. The highest increase of R_n_ and R_d_ rates was noted under severe drought in “*A*” and “*P*”; Figure S1). Interestingly, respiratory effects depended on the cultivar. In “*P*” R_d_ exceeded R_n_, both in control conditions of growth and under severe drought treatment, while in “*A*” cultivar the R_d_ exceeded R_n_ rate only under the severe stress. In “*C*” R_d_ was always lower than R_n_, regardless of the drought level. 

PhR rate progressively declined along the stress duration in “*A*” and “*C*”. In contrast, a slight increase of PhR rate was observed after mild drought in “*P*” (Figure S1). In the course of the drought progression, both in “*A*” and (to a lesser extent) in “*C*” cultivar, the rapid increase of the contribution of R_d_ and the decrease of the contribution of PhR in the R_T_ value was noticed. Under mild drought in “*P*” leaves, R_d_ and PhR rates contributed in the other way to R_T_ rate (Figure S1). 

Next, we investigated the dynamic nature of the cauliflower mitochondrial proteome in three cultivars under water deficit. We studied whether diverse pools of drought-responsive mitochondrial proteins accompany the analyzed respiratory alterations in stress-sensitive and stress-tolerant cultivars. 

### 2.2. Specificity of Mitochondrial Proteome Alterations under Drought in Diverse Cauliflower Cultivars

Mitochondrial proteins isolated from curds of cauliflower plants of three investigated cultivars (Section 2.1) grown in control conditions (0) as well as in mild (1) and severe (2) water deficit were resolved by two-dimensional gel electrophoresis (2D PAGE). Experimental variants were analyzed in pairs as follows: “*A1*” vs. “*A0*”, “*A2*” vs. “*A0*”, “*C1*” vs. “*C0*”, “*C2*” vs. “*C0*”, “*P1*” vs. “*P0*”, “*P2*” vs. “*P0*”. 2D gels for nine different variants, including control, were run in triplicate. The Coomassie Brilliant Blue (CBB)-stained master gel (a fused image) was created based on pooled samples containing equal amounts of mitochondrial proteins from all experimental variants, resulting in 370 different spots. The number of spots on the particular 2D gel varied from 231 to 370 between all analyzed variants (including controls) for each cultivar (Figure S2). 

**Figure 1:**
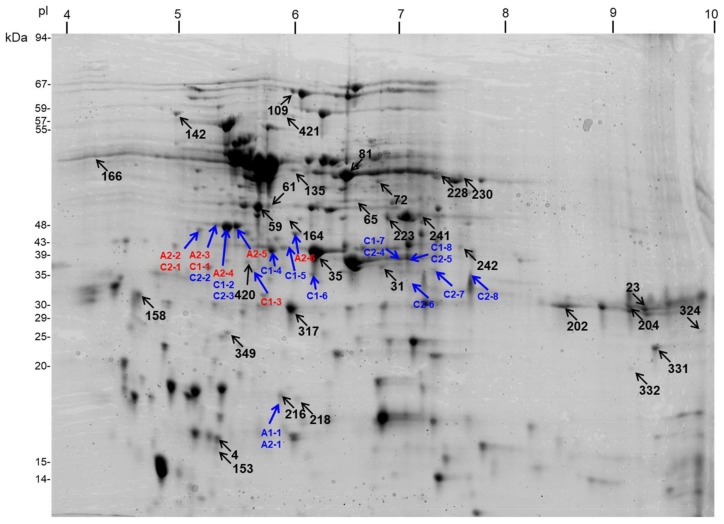
Position of 32 drought-responsive spots (*black arrows*) on Coomassie Brilliant Blue (CBB)-stained two–dimensional (2D) master gel with separated cauliflower mitochondrial proteins (proposed identities of all protein spots are shown in Table S1 and peptide data in Table S4). This map also shows positions of additional protein spots (*blue arrows*) from two-dimensional (2D) gels containing resolved mitochondrial proteins of “*Adelanto*” (A) and “*Casper*” (C), that were cut out and used for the identification of tryptic peptides specific to dehydrin-like proteins (proposed identities for those spots appear in Table S6). Identifiers for spots containing the mentioned peptides are marked *in red* (remaining labels—*in blue*). For molecular mass calibration (kDa) of protein spots, PageRuler Prestained Protein Ladder (Thermo Scientific, Gdańsk, Poland) and Low Molecular Weight (LMW)-SDS Marker Kit (GE Healthcare Poland, Warsaw, Poland) are used. For calibration of spot isoelectric point (pI), Broad pI Kit (GE Healthcare) is used. Further data found in the text.

**Figure 2:**
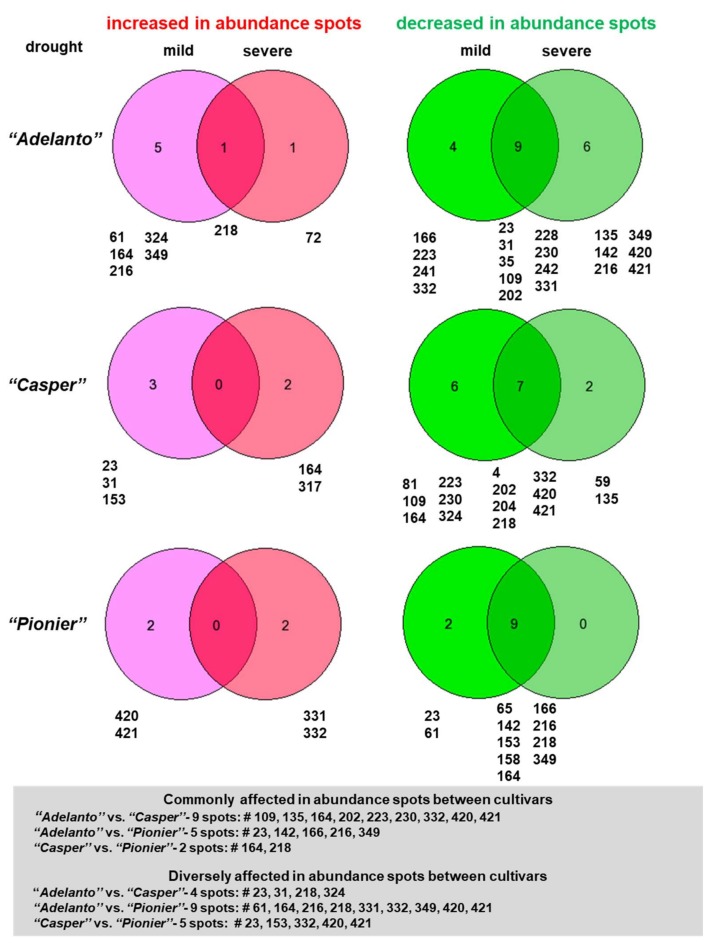
Venn diagrams showing distribution of increased and decreased in abundance specific/common protein spots to mild and severe drought across investigated cultivars. Numbers refer to protein spot identifiers. Increased (magenta and pink diagrams) and decreased (light and dark green diagrams) in abundance spots between cultivars are marked below each diagram; commonly and diversely affected in abundance spots are also listed. Further data in the text.

Thirty-two spots (8.65% of all spots from the master gel) from all cultivars significantly varied in abundance and their positions (Figure 1 and Figure S2) were assessed from three biological replicates. Experimental as well as statistical data for responsive protein spots are shown in Tables S1 and S2 and on Figure S3. Mitochondrial proteomes varied among all cultivars. Massively decreased in abundance spots exceeded those ones which increased in abundance. Under mild drought, four (in “*A*”), six (in “*C*”), and two spots (in “*P*”) were specifically decreased in abundance. In the same conditions, a specific increase in abundance for five, three, and two spots in those cultivars, respectively, was noted. Under severe drought, six, two, and no spots were specifically decreased in abundance, and only one, two, and two spots were specifically increased in abundance in the mentioned cultivars, respectively (Figure 2 and Figure S3). Thus, increased abundance in spots specific to the given drought level dominated over common spots for both treatments. The number of spots diversely affected in abundance between investigated drought-sensitive and drought-tolerant cultivars exceeded the number of such spots within the drought-sensitive cultivars. Conversely, spots commonly affected in abundance increased in number between the drought-sensitive cultivars (Figure 2). Drought responses for the drought-tolerant cultivar (“*P*”) were particularly specific (alterations in abundance related with the distinct set of protein spots when compared to the impact of mild stress). 

In addition, most of protein spots displayed the relationship in abundance, evaluated by the correlation analysis (Table S3). Negatively correlated pairs exceeded in number the positively correlated ones in their abundance across all cultivars and treatments. All significant correlations are strong; the correlation pattern exemplifies the diversity of spot abundance alterations in drought.

### 2.3. Functional Categorization of Drought-Responsive Proteins in Diverse Cultivars of Cauliflower

Proteins from spots collected from the master gel were identified by the tandem mass spectrometry coupled with liquid chromatography (LC-MS/MS). Obtained data were used for searching Mascot against the *Viridiplantae* section of the NCBInr database (version 20160525 containing 88005140 protein sequences). In addition, Gelmap tool (Available online: https://gelmap.de/projects-arabidopsis/) was applied to compare cauliflower and *Arabidopsis* proteomic maps, and to validate MS identifications. Because mitochondrial proteome from the non-green apical part of cauliflower curds was investigated, the use of a 2D PAGE reference map of *Arabidopsis* cell culture mitochondrial proteome was particularly advisable. In some cases, we also used the map representing *Arabidopsis* mitochondrial proteome of green tissues [13]. 

Identities of protein spots are presented in Table S1; individual peptides for each protein spot are also listed in Table S4. All spots represented 91 non-redundant proteins within records that fit to the experimental data. Of this number, 69 non-redundant proteins with the highest probability of identification were found (bolded records in Table S1). Proteins were identified based mostly on high similarity to sequences of diverse cruciferous species. Various respiratory (e.g., ATP synthase, proteins for respiratory complexes (CII and CIV) biogenesis, mostly decreased in abundance), transporter (e.g., diverse voltage-dependent anion channel (VDAC) isoforms and dicarboxylate antiporters) and matrix proteins (ex. heat shock-proteins (HSPs), DNA-binding proteins, RNA editing and translation factors, mitochondrial thioredoxins, diverse multifunctional enzymes for amino acid, carbohydrate, lipid, and nucleotide metabolism, and some novel proteins) responded to drought. For instance, diversely affected in abundance spots between cauliflower cultivars (Section 2.2; Figure 2) included, inter alia, VDAC isoform 2, α/β hydrolase domain-containing protein 11, RNA editing factor 6, copper ion binding protein, mitochondrial elongation factor EF-Tu, single-stranded DNA-binding protein WHY2 (mitochondrial isoform X1), NADH-cytochrome b_5_ reductase-like protein, mitoribosomal protein L21, malonyl-CoA-acyl carrier protein, SWIB/MDM2 domain superfamily protein, HSPs (e.g., HSP70-9) and a few uncharacterized proteins. Selected alterations in protein abundance are further discussed in Section 3.2.

Based on *Arabidopsis* protein orthologs, next we used the functional classification by the Munich Information Center for Protein Sequences (MIPS) at VirtualPlant 1.3 (Available online: http://virtualplant.bio.nyu.edu; [50]) for the clustering of drought-responsive proteins resolved on 2D gels into the functional categories (Table S5). The majority of proteins belonged to the class participating in various metabolic routes (ca. 44%). Next classes were represented by C compounds and carbohydrate metabolism (23.1%), amino acid metabolism (18.7%), cell rescue, defense and virulence (16.5%), energy conversions (12,1%) as well as in N and S metabolism proteins (7.7%). Participation of electron transport (7.7%), complex cofactor binding proteins (6.6%) and folding proteins (4.4%) in drought response were also distinctive (Table S5). 

In the case of drought-tolerant “*P*” cultivar, some differences in the abundance of functional categories across proteins compared to the all-cultivar data were observed. C-compound and carbohydrate metabolism and energy conversion proteins contributed to a lesser degree (18.6 and 9.3%, respectively), however, N- and S-metabolism proteins, folding and stress-responding proteins contributed to a greater extent (9.3%, 7%, and 16.3%, respectively). Stress response proteins within cell rescue, defense, and virulence category were significantly enriched in drought-tolerant cultivar (16.3%) (Table S5). 

Accordingly to the data in Tables S1 and S2, proteins within spots No. 23, 135, 164, 204, 331, 332, and 421 seem to be the best candidates for stress tolerance in cauliflower. These are mainly VDAC isoforms, ATP synthase subunit β (ATP2), At1g18480-like protein, WHY2 factor (isoform X1), OB-fold-like protein, and NADH-cytochrome b_5_ reductase-like protein, as well as HSP70-9 (which notably increased in abundance in “*P*”). Thus, they represent quite broad protein classes (oxidative phosphorylation (OXPHOS) proteins, as well as proteins involved in metabolite exchange, mitochondrial DNA-binding proteins, some enzymes and chaperones). Additional candidate proteins for the drought tolerance, according to results of immunoassays (Section 2.4 and Section 2.5) are listed in the final Conclusions section. 

### 2.4. Abundance of Key Matrix Proteins, cyt. c and Components of Dissipating Energy Systems is Diversely Affected under Two Drought Levels across Cauliflower Cultivars

**Figure 3:**
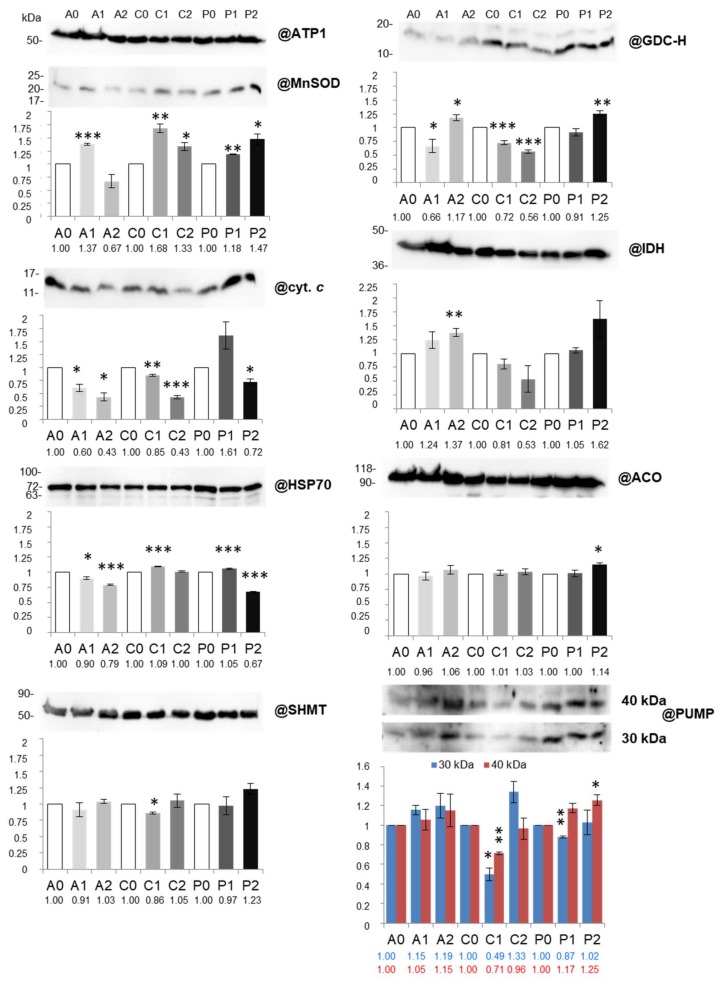
Abundance of Mn-superoxide dismutase (@MnSOD), cytochrome *c* (@cyt. *c*), heat shock protein 70 (@HSP70), serine hydroxymethyltransferase (@SHMT), glycine decarboxylase subunit H (@GDC-H), isocitrate dehydrogenase (@IDH), aconitase (@ACO) and 40 and 30kDa isoforms of uncoupling proteins (@PUMP) in mitochondria isolated from control grown “*Adelanto*”, “*Casper*”, and “*Pionier*” plants (A0, C0, P0), plants grown in moderate (A1, C1, P1) and severe water deficiency (A2, C2, P2, respectively). Results from representative SDS-polyacrylamide gel blots using listed antibodies (@) are shown. For loading control, antibody against mitochondrial ATP synthase subunit α (@ATP1) is used. For molecular mass calibration, PageRuler Prestained Protein Ladder (Thermo Scientific, Gdańsk, Poland) is applied. Protein molecular mass is indicated in kDa. All results are presented as mean values (±SE) from triplicate detection. Significant alterations are marked with asterisks: ***, *p* < 0.001, **, *p* < 0.01, *, *p* < 0.05 versus control values for each cultivar. Further data in the text.

To assess the abundance of additional, drought-responsive mitochondrial proteins and to validate abundance of selected proteins identified in 2D spots, we performed Western immunoassays (Figure 3), using specific antisera dedicated against investigated proteins. 

The accumulation profile of those proteins varied depending on cultivar and stress intensity. Under mild drought, a significant decrease in glycine decarboxylase subunit H (GDC-H) abundance was visible only in “*A*” and “*C*” mitochondria, however, in severe stress, such a decrease was detected only in “*C*”; in other stress variants (especially in “*P*”) GDC-H abundance increased. Strikingly, another important photorespiratory enzyme, serine hydroxymethyltransferase (SHMT), decreased in abundance in “*C*” under mild drought only, and remained stable in other experimental variants. Changes in GDC-H and SHMT abundance analyzed by immunoassays were not fully associated with accumulation of spots No. 158, 228, 230 and 241 (Figure 3; Table S1). 

The abundance of mitochondrial heat shock protein HSP70 decreased in “*A*” and “*P*” (particularly in severe drought) and slightly increased in “*C*” under mild stress (Figure 3). Interestingly, changes in HSP70 abundance roughly agreed with 2D PAGE data (spot No. 421; Table S1). Accumulation of isocitrate dehydrogenase (IDH) increased only in severe drought in “*A*” (Figure 3); in contrast, spots No. 223 and 241 containing this protein under mild drought were decreased in abundance in the same cultivar (Table S1). Aconitase (ACO) abundance (the applied antibodies can cross-react with both ACO2 and ACO3 isoforms) was relatively stable, and it was increased only in “*P*” under severe treatment; ACO2 decrease in abundance in drought-sensitive cultivars was detected by 2D PAGE (spot No. 109; Table S1). The abundance of Mn superoxide dismutase (Mn-SOD) increased in almost all investigated stress conditions, except severe drought treatment in “*A*” (Figure 3). Accordingly, we noticed variations in the abundance of spot No. 4 containing a mitochondrial-like glutaredoxin in this cultivar (Table S1). The intramitochondrial pool of cytochrome *c* (cyt. *c*) was decreased in severe drought in all investigated cauliflower cultivars, but in mild stress, among drought-sensitive ones (“*A*” and “*C*”; Figure 3) only. 

**Figure 4:**
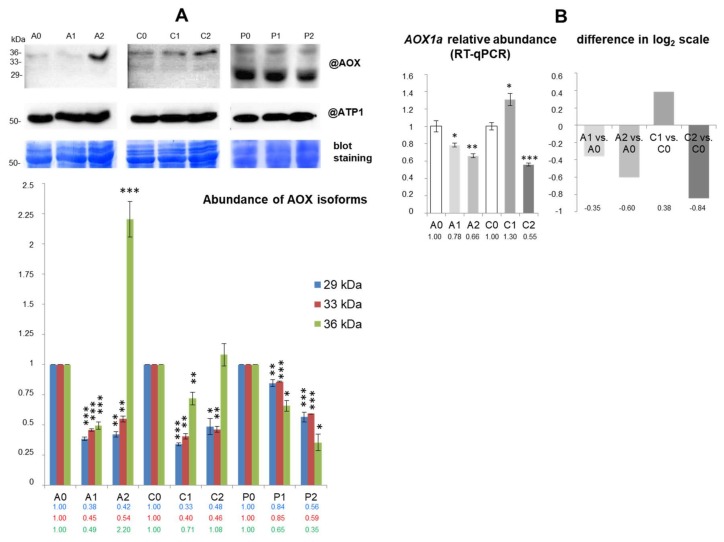
AOX protein and mRNA abundance in mitochondria isolated from control grown “*Adelanto*”, “*Casper*”, and “*Pionier*” plants (A0, C0, P0), plants grown in moderate (A1, C1, P1) and severe water deficiency (A2, C2, P2, respectively). (**A**) Analysis of detected AOX polypeptides (29, 33, 36 kDa) on representative SDS-polyacrylamide gel blots using respective antibodies (@AOX). For loading control, antibody against mitochondrial ATP synthase subunit α (@ATP1) is used. Equal protein loading is also shown by Coomassie Brilliant Blue (CBB) staining of Western blots. For molecular mass calibration, PageRuler Prestained Protein Ladder (Thermo Scientific, Gdańsk, Poland) is applied. Protein molecular mass is indicated in kDa. (**B**) Relative abundance of *AOX1a* by reverse transcription quantitative PCR (RT-qPCR). Graph *at the left*, relative abundance normalized to average level (mean log expression = 1). Graph *at the right*, differences in log_2_ scale between treated and control variants. For normalization, actin1 (*ACT1*) is used. Results of analyses are presented as mean values (±SE) from triplicate detection. Significant alterations in (**A**) and (**B**) are marked with asterisks: ***, *p* < 0.001, **, *p* < 0.01, *, *p* < 0.05 versus control values for each cultivar. Further data found in the text.

In addition, the abundance of selected components of energy dissipating systems was investigated. Antisera against potato detected two isoforms of plant-uncoupling mitochondrial proteins (PUMPs) of 30 and 45 kDa in cauliflower mitochondria. The abundance of both polypeptides representing isoforms of uncoupling proteins was decreased significantly in “*C*” and to a lesser extent in “*P*” under mild drought. However, in severe drought, we noticed visible increase in abundance of PUMP 40 kDa in “*P*” (Figure 3). We also characterized the alternative oxidase (AOX) abundance. Monoclonal antibodies raised against the *Sauromatum guttatum* enzyme cross-reacted with three polypeptides of 29–36 kDa in cauliflower mitochondria (Figure 4A). Polypeptides of 29 and 33 kDa were significantly decreased in their abundance in all investigated cauliflower cultivars; in “*P*”, such a trend was less pronounced than among drought-sensitive cultivars. In contrast, a massive accumulation of AOX 36 kDa protein after severe drought treatment of “*A*” was noted. In general, variations in abundance of PUMP 40 kDa and AOX 36 kDa isoforms differentiate drought-sensitive and tolerant cauliflower cultivars.

### 2.5. Pattern of Dehydrin-Like Proteins (Dlps) in Cauliflower Mitochondria is Affected in Abundance by Drought

**Figure 5:**
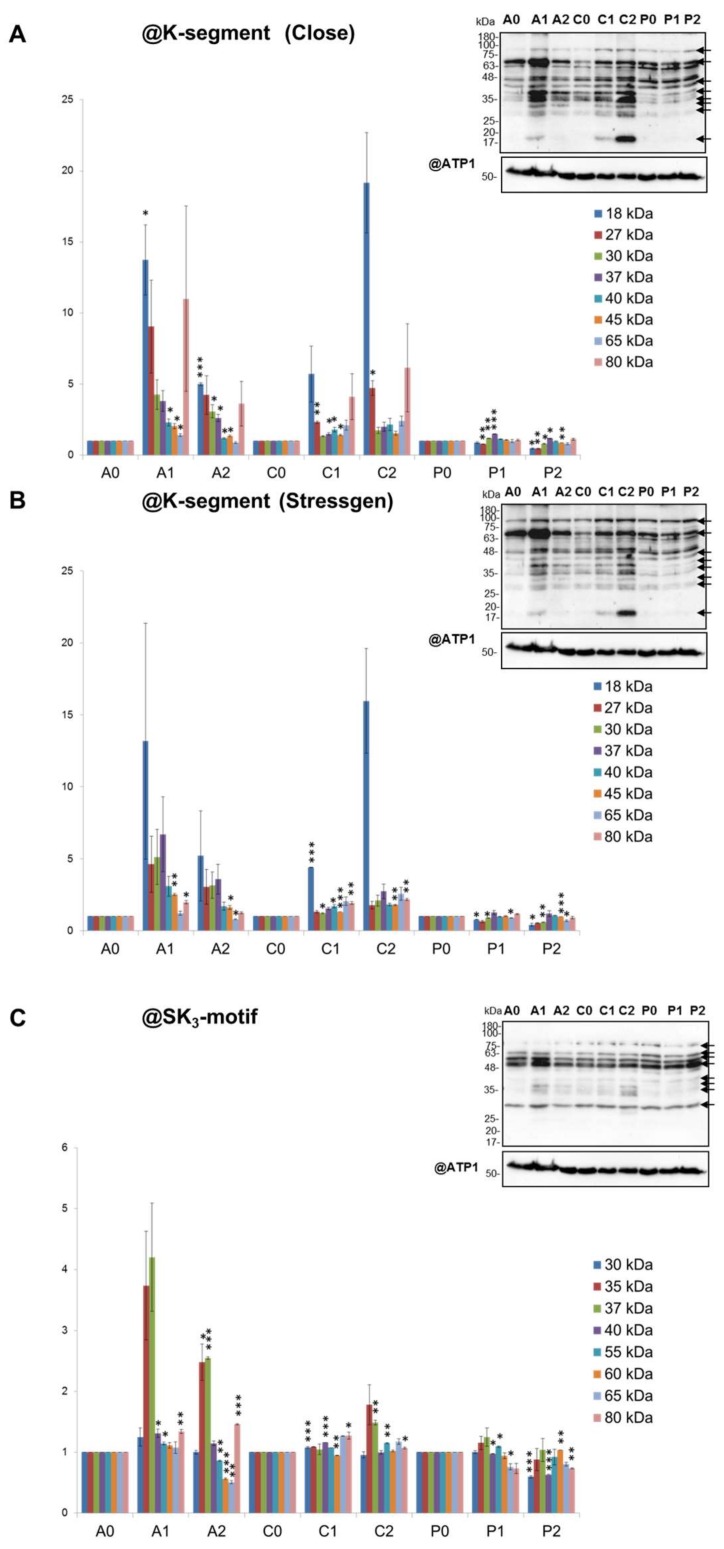
(pp. 11 and 12) Dlps abundance in mitochondria from control grown “*Adelanto*”, “*Casper*” and “*Pionier*” plants (A0, C0, P1), plants grown in moderate (A1, C1, P1) and severe water deficiency (A2, C2, P2, respectively). Results of analysis of dlps of various size (in kDa) on representative SDS-polyacrylamide gel blots with antibodies directed against (**A**) dehydrin K-segment from Close [51] (@K-segment (Close)) or from (**B**) Stressgen (@K-segment (Stressgen)), or (**C**) antibodies recognizing dehydrin SK_3_ motif (@SK_3_) are shown. For loading control, antibody against mitochondrial ATP synthase subunit α (@ATP1) is used. For molecular mass calibration, PageRuler Prestained Protein Ladder (Thermo Scientific, Gdańsk, Poland) is applied. The protein molecular mass is indicated in kDa. Investigated dlps are marked by arrows on gel blots. Significant alterations from triplicate detection in (**A**), (**B**) and (**C**) are marked with asterisks: ***, *p* < 0.001, **, *p* < 0.01, *, *p* < 0.05 versus control values for each cultivar. Further data in the text.

We also investigated the pattern of cauliflower mitochondrial dehydrin-like proteins (dlps) under mild and severe drought (Figure 5). According with our previous data [49], three independent dehydrin-specific antisera were used (Section 4.8). 

Obtained results indicate the response in abundance of low-molecular weight dlps (18–80 kDa) in “*A*” and “*C*”. Stressgen antibodies detected the most evident increase in abundance of 18 and 27 kDa dlps (and to a lesser extent, in “*A*” for 37 kDa protein) under two drought levels in “*A*” (mild drought) and “*C*” (severe treatment). Large-sized dlps (ca. 80 kDa) also markedly increased in abundance in drought-sensitive cultivars, although the highest increase in abundance of those proteins was noted under mild stress in “*A*” (Figure 5A,B).

Antisera against dehydrin SK_3_-motif recognized smaller alterations, but there was a significant increase in abundance of ca. 30/35 kDa dlps under both drought conditions in “*A*” and under severe drought in “*C*” (Figure 5C). Alterations in the abundance of middle and large-sized dlps were less pronounced; the accumulation of dlps of 55–65 kDa slightly decreased under severe drought in “*A*”. Similar results were observed for 30 and 40 kDa dlps in “*P*”. 

Overall, dlps in “*P*”, contrary to “*A*” and “*C*” were relatively stable in abundance under the analyzed adverse conditions, and dlps of 18 and 27 kDa differentiate best the drought-sensitive from the drought-tolerant cauliflower cultivars.

Relationships between abundance of immunodetected proteins (Section 2.4 and Section 2.5) were evaluated by correlation analysis (Table S3). We found strong positive correlation between IDH and GDC-H abundance, as well as between components of energy-dissipating systems (AOX 29 and 33 kDa isoforms, and in addition, AOX 33 kDa and PUMP 40 kDa isoforms). Positive correlations involved also dlps of various sizes, confirming their participation in drought response. On the contrary, accumulation of relatively stable IDH negatively correlated with drought-affected HSP70. Similarly, accumulation of PUMP 30 kDa isoform negatively correlated with and cyt. *c*, and dlp 18 kDa accumulation with GDC-H and PUMP 40 kDa isoform abundance. Negative correlations involved also AOX 33 kDa isoform (not induced by drought in our study) and dlp 80 kDa (Table S3).

### 2.6. Identification of Drought-Responsive Spots Containing Putative Dehydrin-Like Proteins

**Figure 6:**
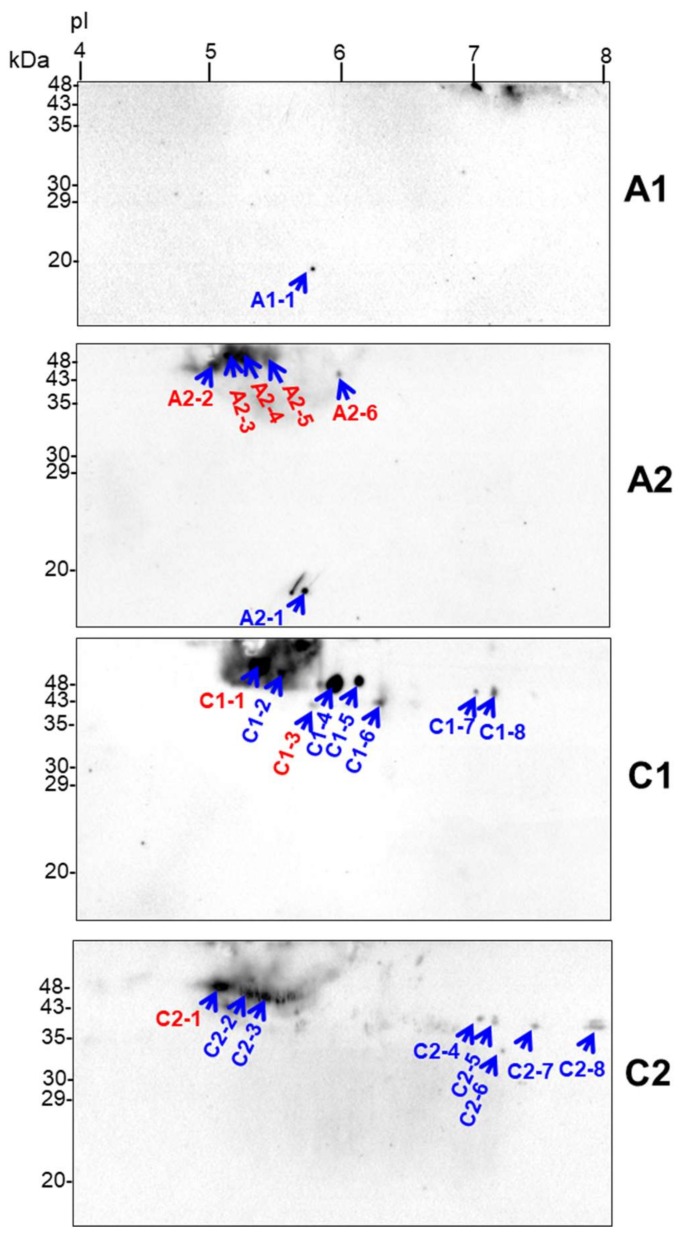
Representative pattern of dlps of “*Adelanto*” and “*Casper*” plants grown in moderate (A1, C1) and severe drought (A2, C2, respectively) immunodetected with antibodies directed against dehydrin K-segment [52] on two dimensional (2D) blots. Spots referring to detected proteins that were cut out from the respective 2D gels for protein identification by liquid chromatography-tandem mass spectrometry (LC-MS/MS, *blue arrows*) also appear in Figure 1 (denoted *in blue* and *red*). Proposed identities for those spots and all tryptic peptide data are indicated in Table S6. Identifiers of spots containing tryptic peptides specific to dehydrins are marked *in red* (remaining labels are shown *in blue*). For molecular mass calibration (kDa) of protein spots, PageRuler Prestained Protein Ladder (Thermo Scientific, Gdańsk, Poland) and Low Molecular Weight (LMW)-SDS Marker Kit (GE Healthcare Poland, Warsaw, Poland) are used. For calibration of spot isoelectric point (pI), Broad pI Kit (GE Healthcare) is applied. Further data found in the text.

The most notable changes in the accumulation of dlps were noticed in “*A*” and “*C*” cultivars under all analyzed drought conditions (Section 2.5). To further characterize the drought-responsive cauliflower dlps, we prepared 2D blots with separated whole mitochondrial proteins from “*A*” and “*C*” cultivars submitted to mild and severe drought, and we used such blots to immunodetect dlps (within the particular protein spots) by antisera against dehydrin K-segment (Section 4.8). Results of the dlp immunodetection on 2D spots are shown in Figure 6. 

Due to the assay sensitivity, we were able to detect several spots per investigated cultivar/treatment within pI range of ca. 5–8 and molecular weight of 18–48 kDa (35–48 kDa mostly) with varying abundance under the water deficit (Section 2.5). However, the only single spot was immunodetected in “*A*” cultivar under mild drought. Certain spots (C1-7 and C1-8, C2-4 to C2-6) migrated in more neutral pI values, whereas the others (C2-7 and C2-8) represented basic proteins. Based on immunoassay results, we cut out all immunodetected protein spots from 2D gels, and proteins were identified by LC-MS/MS. Position of all those spots from Western blots are superimposed on the spot pattern within the master gel image (Figure 1), and protein identities within individual spots are shown in Table S6. 

Notably, only selected spots contained dehydrin-related tryptic peptides that showed high similarity of their protein sequences to the selected *Brassica* dehydrins. These were: five spots (A2-2 to A2-6) in “*A*” cultivar under severe drought, two spots (C1-1 and C1-3) in “*C*” under mild stress, and the single spot (C2-1, all indicated in red on Figure 6) in the latter cultivar under severe water deficit. Detected tryptic peptides were highly similar to early response dehydrins (ERD14 and ERD14-like) from various *Brassica* species, particularly *B. oleracea* var. *oleracea* and *B. rapa* (GenBank accession.version identifiers XP_013592580.1 and XP_009128158.1, respectively; Table S6). However, analyzed protein spots also contained highly abundant mitochondrial proteins (listed in Table S6). Finally, we focused on the comparison of selected proteomic and transcriptomic responses important for cauliflower mitochondrial biogenesis in drought, including abundance profiling of mRNA coding for the identified dehydrins.

### 2.7. mRNA Abundance and Coordination of Mitochondrial Biogenesis in Drought 

We examined the abundance of selected nuclear transcripts coding the investigated drought-responsive proteins. RT-qPCR assays were carried out with application of primers specific for the dedicated cDNA fragments (Table S7). As an internal calibrator of gene expression, cauliflower actin1 (*ACT1*) mRNA was applied (the partial sequence was previously cloned and deposited in GenBank under accession.version KC631780.1; [13]). The cauliflower *AOX1a* partial sequence was previously cloned and deposited in GenBank (accession.version KC631778.1; [13]). Accumulation of *AOX1a* mRNAs was significantly increased under mild drought in “*C*”; on the contrary, they were strongly decreased in abundance under mild and severe drought in “*A*” and severe drought in “*C*” (Figure 4B). 

**Figure 7:**
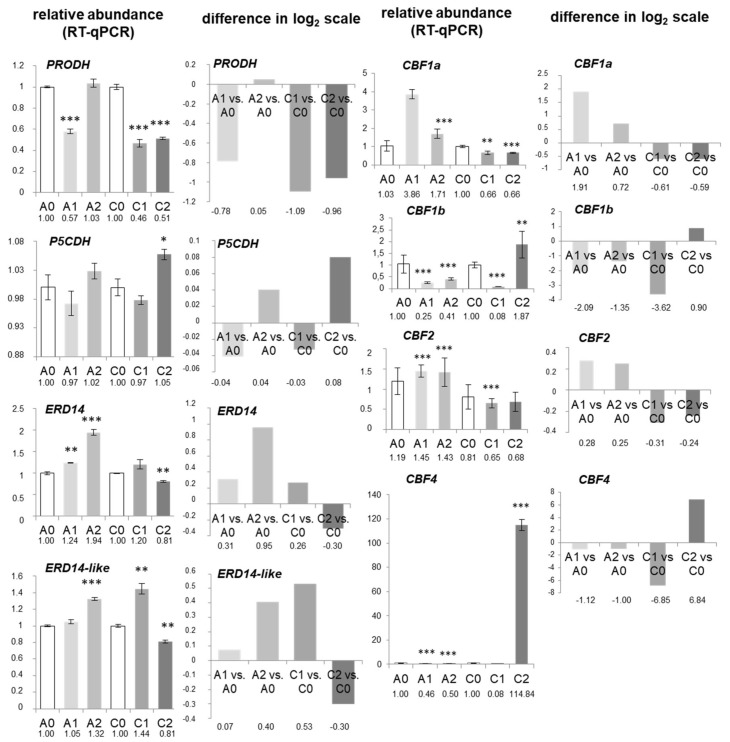
Relative abundance of transcripts (by reverse transcription quantitative PCR (RT-qPCR)) for cauliflower proline dehydrogenase (*PRODH*), Δ-1-pyrroline-5-carboxylate dehydrogenase (*P5CDH*), ERD14 dehydrin (*ERD14*), ERD14-like dehydrin (*ERD14-like*), as well as transcripts for transcription factors (*CBF1a*, *CBF1b*, *CBF2*, *CBF4*) in control grown “*Adelanto*”, “*Casper*”, and “*Pionier*” plants (A0, C0, P0), plants grown in moderate (A1, C1, P1) and severe drought (A2, C2, P2, respectively). Differences in log_2_ scale between treated and control variants are also shown. In all cases, for normalization, actin1 (*ACT1*) is used. Significant alterations are marked with asterisks: ***, *p* < 0.001, **, *p* < 0.01, *, *p* < 0.05 versus control values for each cultivar. Further data found in the text.

We studied the abundance of *PRODH* and *P5CDH* transcripts coding for important Pro catabolism enzymes (proline dehydrogenase and Δ-1-pyrroline-5-carboxylate dehydrogenase, respectively). Generally, the abundance of *PRODH* mRNA decreased in “*C*” in both drought treatments; the most severe impact was visible under mild drought. In “*A*” cultivar, a significant decrease of *PRODH* mRNA abundance was noted only in mild stress. Contrasting with this, *P5CDH* transcript accumulation was only markedly elevated in “*C*” under severe drought (Figure 7).

The abundance of mRNA coding for ERD14 and ERD14-like dehydrins (Section 2.6) was also determined. The highest increase in both transcript abundance was noticed in “*A*” cultivar in mild and severe drought, which is in line with the induction of *ERD14* gene expression within the short dehydration period, as well as with the presence of dehydrin-related tryptic peptides in analyzed protein spots. Notably, *ERD14* and *ERD14-like* transcripts were significantly decreased in abundance in “C” under severe water deficit (Figure 7). 

Finally, we investigated the accumulation of mRNAs coding for five selected transcription factors (CBF1a, CBF1b, CBF2, CBF4) related to stress response. *CBF1a* and *CBF2* transcripts were increased in abundance in “*A*”, but they declined in “*C*” cultivar, as drought progressed. Strikingly, *CBF1b* mRNA showed substantial increase in abundance only in “*C*” in mild and severe drought treatments. Increase in abundance of *CBF4* mRNAs was also very well noted under severe water deficiency in “*C*” (Figure 7). 

Correlation analysis revealed no significant relationship between accumulation pattern of investigated transcripts (Table S3).

## 3. Discussion

### 3.1. Physiological Response of Cauliflower Cultivars under Mild and Severe Drought

We studied leaf respiratory responses within three distinct cauliflower cultivars with varying drought tolerance. Previously, we showed that the respiratory rate exceeded the photosynthetic one in cauliflower leaves under severe (but not moderate) water deficiency [52]. This emphasizes the importance of adequate respiratory adaptations in this species under the mentioned unfavorable conditions. Respiration and photorespiration become a part of the complex network response under water deficiency, and photorespiration participates in oxidative damage avoidance while optimizing photosynthesis [53]. We noticed enhancement of respiration among drought-sensitive cultivars (contrary to “*P*”) under mild drought; the respiration rate was often decreased in severe treatment. Interestingly, respiratory decline in such conditions is a well-known phenomenon (also for *Brassica* species) and R_n_ rate is affected much under fast drying [19,30,54]. Mitochondrial R_d_ can be also inhibited by drought; it markedly increases in prolonged drought but declines under short water deficit [55,56,57]. In our case, the effect depended on the cultivar. Generally, drought-sensitive crop cultivars exhibit larger R_T_ decreases, than sensitive ones; nevertheless, both effects could be reversed under drought recovery [58]. 

The increase of photorespiratory to gross CO_2_ assimilation ratio under drought is often required in order to protect the photosynthetic machinery against photoinhibition. In field-grown *Gossypium hirsutum*, drought resulted in affected stomatal conductance and elevated respiratory and PhR rates, while photosynthetic electron transport was not affected [40,55,59]. The progressive alteration of PhR rate in “*A*” and “*C*” along the stress duration is in line with Liu et al. [60] data, suggesting that the PhR in drought-sensitive cultivars cannot be a major energy dissipation strategy, as was reported for some Asiatic and Mediterranean-originated plant species. In contrast to that, the visible increase of PhR rate in the mild treatment in “*P*” is known among drought-tolerant species [61]. Notably, the general trend in PhR response could be reversed at the early vegetative stage in some species with varying drought tolerance [62]. In drought-tolerant and drought-sensitive cultivars of *Malus domestica*, even moderate drought resulted in major PhR decline [63].

Overall, observed alterations in the respiratory parameters coincide with the level of drought tolerance among investigated cauliflower cultivars and suggest distinct regulation of drought physiological responses in “*P*”.

### 3.2. Mitochondrial Response to Drought Involves Diverse Multifunctional OXPHOS, Transporter and Matrix Proteins in Various Cauliflower Cultivars

We also investigated drought-resulted alterations within the cauliflower mitochondrial proteome. Since the experimental molecular mass of protein spots corresponded roughly to the theoretical one, we are rather convinced that investigated stress conditions do not result in excessive proteolysis [19]. Proteins identified in some double spots (e.g., No. 228, 230) showed similar responses, which is in favor of the correctness of their assignments (Table S1). We applied functional classification for the clustering of drought-responsive proteins (Table S5). Some functional classes are often underrepresented, highlighting the relevance of organelle-specific studies; on the other hand, key stress-related enzymes (for carbohydrate and amino acid metabolism) are often overrepresented in drought response [7,45,64,65], whereas proteins related to protein folding and degradation may decline in abundance [30]. 

Energy and carbohydrate metabolism proteins play a significant role in drought response [44,45]. Malate dehydrogenase (MDH), succinyl-CoA ligase subunit β and ACO2 (spots No. 35, 59 and 109) were decreased in abundance in drought-sensitive cauliflower cultivars (the present study), in roots of drought-sensitive rapeseed (*Brassica napus*) cultivar [35], as well as in other reports elucidating the impact of the extended water deficit on the plant proteome [46,48], but contrary to the Ndimba et al. [18] study on the sorbitol-induced drought. Variations of MDH accumulation are linked to increased NADH demands [38]. ACO2 isoform is predominantly localized in plant mitochondria, and ACO-containing complexes were shown to be unstable in stress [13,66]. 

Decrease in abundance of subunits of cauliflower OXPHOS complexes (e.g., CI and CII) was similar to some studies [19,67]. Massive decrease in accumulation of CII subunit 1 and 5 (SDH1 and SDH5; spots No. 4, 23, 204; Table S1) differed from barley (*Hordeum vulgare*) drought response pattern [47]; CII subunits were increased in abundance after drought recovery in *Populus euphratica* [2]. Contrary to other reports [31,44], CIV subunits were unaffected in our study. ATP synthase was one of the expected complexes that appeared decreased in abundance in cauliflower mitochondria [13]. Its assembly may be affected as diverse cellular energy demands rise under stress [68,69,70]. According to our data (spots No. 135, 166), a decrease in abundance of ATP synthase subunits under water deficit was reported by a number of studies [16,35,39], contrary to other ones [43]. Notably, ATP synthase subunit 24 kDa was increased in abundance in “*C*” cultivar under severe drought (spot No. 317; Table S1). 

Water deficit results in decrease in abundance of pyruvate dehydrogenase (PDH) subunits [19]. However, alterations in abundance of *Hippophae rhamnoides* PDH E1 subunit α in drought [65] contrasts with our data (decreased in abundance spots No. 223, 241 for “*A*” and “*C*”; Table S1). To enhance pyruvate import to mitochondria, *NRGA1* coding for a mitochondrial pyruvate carrier is often co-expressed with other carrier genes [71,72]. Genes for mitochondrial dicarboxylate transporters may be induced in water deficit [67]. In our study, PDH decrease in abundance was not associated with co-regulation of any of the specific substrate carriers among proteins affected in abundance under drought. The accumulation of dicarboxylate/tricarboxylate mitochondrial transporters declined in drought-sensitive cultivars instead (spots No. 202, 204; Table S1). 

A decrease in abundance of mitochondrial processing peptidase subunit β (MPPβ; spot No. 166) in “*A*” (mild stress) and “*P*” (all treatments) was noted, in addition to translocase of the inner mitochondrial membrane subunit (TIM44-2-like; spot No. 230) in “*A*” (Table S1). Regulation of genes coding for MPP subunits in stress has been already reported [14,18,40,46,67]. It is nonetheless known that drought may alter protein import into plant mitochondria [73]. Results of our study indicate some perturbations in the general import pathway.

Some spots (No. 23 (VDAC2-like and adenosine nucleotide translocator protein) and 31 (hydrolase domain-containing protein and carbonic anhydrase)) showed inconsistent alterations among drought-sensitive cultivars [68]. Distinct VDAC isoforms which increased in abundance in *Brassica rapa* under prolonged drought and in drought-tolerant wheat cultivars [32,41,48] contrasted with the decreased accumulation of VDAC isoforms in drought-sensitive cauliflower cultivars (spots No. 23, 202-204; Table S1). 

In general, distinct alterations within the mitochondrial proteome are potentially associated with drought tolerance (Section 2.3; [45]). Such alterations encompass mitochondrial DNA/RNA-binding proteins, chaperonins, heat-shock proteins, as well as a number of enzymes for mitochondrial metabolism. Interestingly, we did not notice any biases towards protein spots increased in abundance in drought-tolerant cultivar, contrary to Mohammadi et al. [35], however, we observed some differences in the distribution of functional categories across responsive proteins (Section 2.3; Table S5). ssDNA-binding proteins, as well as proteins related to RNA metabolism and translation (e.g., RNA editing factors 1 and 6, and mitochondrial EF-Tu), were diversely affected in abundance between drought-sensitive and tolerant cauliflower cultivars (spots No. 59, 61, 218, 331, 332). Also, the abundance of SWIB/MDM2 domain superfamily protein, calcineurin-like metallophosphoesterase superfamily, sucrose/ferredoxin-like proteins, mitoribosomal protein L21, chaperonin and HSP70 isoforms differentiated cultivars with diverse drought tolerance (spots No. 153, 164, 349, 420, and 421). A prevalent decrease in abundance after mild drought treatment and more specific alterations in protein spot accumulation in severe conditions likely represent specific adaptations in the cauliflower mitochondrial proteome to diverse drought conditions. However, functional implications of the observed proteomic alterations require further exploration.

### 3.3. Diverse Variations in Abundance of Matrix Proteins, cyt. c, Components of Dissipating Energy Systems and Dehydrin-Like Proteins Across Drought Treatments/Cultivars

In order to obtain a more complete view on the abundance of additional, drought-affected proteins we extended results of 2D PAGE analysis by Western immunoassays. 

GDC and SHMT belong to important photorespiratory enzymes, often regulated in stress conditions (including drought) [15,19,30,40,45,68]. Ford et al. [41] reported contrasting regulation of GDC, as well as SHMT, which depended on drought tolerance. In our study, changes in GDC-H abundance roughly corresponded to the decline in the photorespiration rate only in drought-sensitive cultivars under both mild and severe drought (Section 2.1; Figure S1). SHMT abundance did not correlate with GDC-H alterations (Figure 3) and remained relatively stable. In contrast, HSP70 abundance alterations were not associated with drought tolerance (Figure 3). In such conditions, an evident HSP70 increase in abundance was noted in pea mitochondria [19]. The HSP70 and IDH abundance pattern is in line with typical variations of those proteins in stress [7,18,41]. Diverse variations in the abundance of ACO isoforms may be a part of the adaptive response of Krebs cycle, due to the altered NADH and carbon skeleton demands in drought. In some cases, however, enzymes of Krebs cycle are massively decreased in abundance in water deficiency [74]. 

Mn-SOD displays distinct expression pattern between cultivars with variable drought sensitivity, often by the increase and decrease in abundance in drought and drought recovery, respectively [14,30,32,41,44,75]. Cauliflower Mn-SOD increased in abundance in most of all investigated treatments and cultivars; the maximal increase was observed among drought-sensitive cultivars (Figure 3) and was accompanied by variations in abundance of spot No. 4 containing mitochondrial-like glutaredoxin in “*C*” (Table S1), suggesting that redox regulation likely accompanies drought response [2]. 

According to our results, cyt. *c* can be released from cauliflower mitochondria even under the first level of water deficiency. Such phenomenon often accompanies programmed cell death (PCD), which indeed was suggested by us in temperature stress response [13]. However, in some plant species (e.g., in pea mitochondria), drought did not result in PCD appearance [19]. 

Plant mitochondrial energy dissipating systems including AOX, PUMPs, and membrane channels functionally modulate drought response by influencing key signaling pathways [8]. We used antisera that were able to immunodetect respective cauliflower PUMP isoforms. An observed decrease in abundance prevailed the pattern, and only in severe drought was the increase in PUMP 40 kDa abundance in “*P*” noted (Figure 3). Also, the abundance of selected PUMP isoforms in pea mitochondria was increased in drought [19]. To extend our proteomic data on further components of energy dissipating systems, we also investigated AOX protein accumulation. This enzyme controls respiration rate and photosynthetic efficiency in drought, and often increases in abundance during severe water deficit and re-watering [22,76,77]. Three polypeptides of 29–36 kDa in cauliflower mitochondria [13] likely represent AOX isoforms resulting from the expression of *AOX* gene family and diverse posttranslational modifications of this protein (Figure 4A). Our results contrast with the data of Taylor et al. [19], who showed drought-affected induction of similarly sized 31 kDa pea isoform. However, abundance of AOX polypeptides could be substantially increased among drought-sensitive cultivars.

Under progressing drought, abundance of late embryogenesis abundant (LEA) proteins preserving the stability of membrane proteins and adjusting intracellular osmotic pressure often increases [32,78]. Some LEA proteins, including dlps, were found to be mitochondrially-localized in a number of crop species [79,80,81,82]. Our previous report showed that accumulation of mitochondrial dlps was altered in abundance under temperature stress and after stress recovery [49]. We extended those analyses to three cultivars of cauliflower and to mild and severe drought treatments (Figure 5) using three independent antisera. Broadly-sized dlps responded in abundance as a part of mitochondrial adaptations to water deficiency, because such a response was visible only in drought-sensitive cultivars. The stable dlp accumulation in “*P*” mitochondria agrees with drought tolerance of this cultivar and with the relatively lower increase in dehydrin mRNA abundance (e.g., *DHN8*) under progressed drought among highly adapted plants [36]. Alterations in dlps abundance, as well as the induction pattern of other dlps in drought (Figure 5), substantially extend the data on mitochondrial proteins related to dehydrins in *Brassicaceae*.

Owing to results on the massive increase of dlps abundance under investigated drought treatments in stress-sensitive cultivars, we identified putative dlps involved in response to water deficit in those cultivars by tandem MS. Protein spots immunopositive with dehydrin K-segment-specific antibodies were selected, and several of them contained tryptic peptides with high sequence similarity to selected *Brassica* dehydrins (Figure 6, Table S6). Due to the fact that highly abundant proteins in spots would hamper identification of dlps, we did not employ protein microsequencing for identification of chosen dlps, and subsequent amplification and cloning full-length cDNAs. We were unable to determine the N-terminal sequence for the given dlps. Notably, depletion of any abundant proteins would enhance the risk of sample cross-contamination. 

### 3.4. Some Transcriptomic Responses and the Coordination of the Mitochondrial Biogenesis in Drought 

We analyzed transcriptomic responses to drought connected with the profiling of the abundance of some nuclear transcripts. Since observed proteomic alterations (particularly those connected with dlps abundance) were especially distinctive among drought-sensitive cultivars, we focused on such experimental variants only for this part of our study. 

The choice of *AOX1a* mRNAs for our study can be justified by the fact that accumulation of *AOX1a* transcripts is often affected by diverse stress stimuli [83]; other members of the AOX family, (e.g., *AOX1d*)*,* can be increased in abundance in drought, as well [31]. In our study, the most severe drought, the most intense decrease of *AOX1a* accumulation in “*A*” was observed. A major imbalance between protein and mRNA abundance in “*C*” and “*A*” cultivars was noticed. In “*A*”, two immunoreactive AOX protein bands decreased in abundance under mild and severe stress (which is in line with *AOX1a* transcript accumulation pattern in the same conditions), whereas the third band (36 kDa) notably increased in abundance in the severe treatment (Section 2.4; Figure 4). In this cultivar, under severe drought, the lowered amount of *AOX1a* mRNA contrasted with enhanced accumulation of 36 kDa AOX isoform. In “*C*”, no significant increase in abundance of AOX protein was visible, contrary to the notable increase in abundance of *AOX1*a transcripts under mild drought. Thus, *AOX1a* mRNA compensatory increase in abundance was accompanied by the decrease in protein abundance and vice versa. Alterations of *AOX1a* mRNAs to drought involved only selected cauliflower cultivars and stress conditions (Figure 4B). Lack of coordination between AOX protein and mRNA may result from synthesis of investigated immunoreactive polypeptides from transcripts coding distinct AOX isoforms (especially 33 kDa protein). Regulation of *AOX1a* mature mRNA abundance may depend on changes in selection of transcripts for translation, diverse mRNA/ribosome interactions and/or affected protein synthesis [7]. The decreased pool of those transcripts in some investigated variants suggests their efficient translation leading to protein excess and thus those mRNAs seems to be fully translatable. 

Participation of non-coding RNAs in the regulation of the abundance of investigated transcripts or their translational efficiency should be also considered. Growing evidence supports the presence of miRNAs (nuclear-encoded and processed before import to mitochondria) or components of miRNA biogenesis machinery within mitochondria [84,85,86,87]. We focused on in silico miRNA candidates that may putatively target *B. oleracea AOX1a* by psRNATarget (Available omline: http://plantgrn.noble.org/psRNATarget/analysis?function=2; [88]) search (Table S8). *Arabidopsis* and *Brassica* miRNAs were taken into account, because of the relative high similarity of *AOX1a* nucleotide sequence (GenBank accession.version KC631778.1) between those genera. According to our data, most of the predicted miRNAs resulted rather in messenger degradation, than in affected translation (Table S8). This does not obviously exclude the possibility that multiple non-coding RNAs could influence *AOX1a* mRNA and protein abundance.

Proline, the potent osmoprotectant, over-accumulates under drought and decreases under drought recovery [7,29,30,89]. Its variations may not simply correspond to the level of drought adaptation [36]. Previously, we showed that cauliflower P5CDH protein increased in abundance both in heat and heat recovery [13]. We postulate the reciprocal regulation of the abundance of *ProDH* as well as *P5CDH* transcripts under severe drought in “*C*”. Decrease in abundance of *ProDH* mRNA was not equal between investigated cultivars, indicating diverse osmolyte accumulation in those cultivars [37].

The respective proteomic responses related with dlps profile were very evident for the drought-sensitive cultivars (Section 2.5). Henceforth, we determined the abundance of transcripts coding ERD14 and ERD14-like dehydrins, because tryptic peptides with high sequence similarity to those proteins were found in protein spots on 2D blots that were immunoreactive with dehydrin K-segment antisera (Section 2.6; Figure 6, Table S6). *ERD* transcripts (including *ERD14*) are known to be highly abundant under ABA, salinity, cold, and drought [64,90,91]. In general, our results suggest that positive transcriptomic response progressing with water deficit coincides with strong increase in abundance of dlps in “*A*” (drought-sensitive) cultivar (Figure 5). 

Finally, to obtain a more complex view on cauliflower drought responses, we examined the abundance of transcripts coding for several nuclear transcription factors (TFs), which did not respond in protein abundance in our study, belonging to C-repeat/dehydration-responsive element binding (CBF/DREB) subfamily (from ETHYLENE RESPONSE FACTOR (ERF) family). These proteins contain conserved DNA-binding domains, and are regulated by a number of stressors, including cold and drought [92]. Notably, DREB subfamily contains at least 91 known members in *B. oleracea*; the whole ERF family and DREB A-4 subgroup are particularly enriched comparing with *Arabidopsis* data. *B. oleracea CBF* genes displayed various expression patterns. In our study, *CBF1a* expression showed contrasting responses between two drought-sensitive cultivars; the most visible increase in abundance was noted in severe drought for “*A*” (*CBF1a*) and “*C*” (*CBF1b*). Results obtained for *CBF1b*, and particularly, for *CBF4* transcripts, roughly agree with the late induction pattern in *B. oleracea* [93]. *CBF4* and *DREB1* regulons were suggested to participate in drought adaptation or enhance drought tolerance, but participation of some other CBFs in this process is still controversial [94,95]. Therefore, our results extend such findings by showing alterations in cauliflower *CBF1a*, *CBF1b*, and *CBF2* mRNA abundance under drought (the latter displayed similar expression profile to *CBF1a*). Rapid responses of *CBF1a, CBF2*, and *CBF4* genes to mild drought suggest that they may participate in positive drought signaling in “*A*” and “*C*”, respectively. 

## 4. Materials and Methods 

### 4.1. Growth of Plant Material and Stress Application

Seeds of three analyzed cauliflower (*Brassica oleracea* var. *botrytis* subvar. *cauliflora* DC) cultivars (“*Adelanto*”, “*Casper*” and “*Pionier*”) were obtained from Bejo Zaden (Warmenhuizen, The Netherlands). Cauliflower seedlings were produced in 0.09 dm^3^ pots filled with peat substrate (Kronen-Clasmann, Gryfice, Poland). Seedlings with 3–4 leaves were transferred to 5 dm^3^ containers. Plants were grown for three months in cultivation chambers at a local breeding station (Poznan University of Life Sciences, Poland) at 23/19 °C (day/night) and 70% relative humidity under photon flux density 200 μmol∙m^−2^s^−1^ (16 h of light/8 h of dark). Stress conditions were applied to plants with developing curds up to a curd diameter of 7–10 cm. The water capacity of the substrate was 40% (*v*/*v*) and under drought stress, the water content decreased to 22% (*v*/*v*) (mild drought, the first level of drought) and to 15% (*v*/*v*) (severe drought, the second level of drought). After the occurrence of the assumed level of drought stress, plants were irrigated to 40% (*v*/*v*) and then drought treatment was applied again and repeated for ca. 2–3 weeks. Curds were harvested immediately after stress treatment cessation. Duration of the drought stress was estimated on the basis of relative water content (RWC) in the soil and in plant leaves and curds. RWC in developed, mature cauliflower leaves in mild drought was achieved at RWC of 94%, 92%, and 95% for “*A*”, “*C*”, and “*P*” cultivars, respectively. Under severe drought, RWC values lasted 69%, 74%, and 73% for the mentioned three cultivars, respectively. RWC of cauliflower leaves under severe drought referred to the third and fourth day of the drought treatment.

### 4.2. Physiological Analyses

Physiological analyses were conducted on well-developed cauliflower leaves with an LCpro+ infrared gas analyzer (ADC BioScientific Ltd., Hoddesdon, UK). To obtain more reliable results, extra experimental replicas (*n* = 8) were used. R_d_ rate was determined according to the Laisk [96] method. CO_2_ assimilation rate representing R_T_ rate was recorded during intercellular CO_2_ concentration (C_i_) decreased to 0 ppm at 22 °C and 50% relative humidity. For each value of photosynthetic photon flux density (PPFD) at 200, 400, and 600 μmol∙m^−2^s^−1^, the linear regression of CO_2_ assimilation (A) versus C_i_ was calculated. The intersection of three regression lines was determined by minimizing the sum of squares of errors between the measured values and calculated for each PPFD level, while minimizing the standard deviation for the intersection. PhR rate was determined as the difference between R_T_ and R_d_ values at C_i_ 0 ppm (the latter one expressed as a given CO_2_ evolution rate at the crossing point of all A/C_i_ curves). R_n_ rate was measured after 30 min of adaptation to the dark. The applied drought treatments were necessary for visible alterations in respiratory and photorespiratory rates (Figure S1). The given drought exposures (Section 3.1) were necessary to observe proteomic and physiological alterations.

### 4.3. Isolation of Mitochondria, Purity Assays, and Protein Determination

Mitochondria from the topmost 5 mm-thick layer of cauliflower curds were extracted by differential centrifugation and purified in Percoll gradients according to Pawlowski et al. [97]. During isolation, the Complete Mini EDTA-free Protease Inhibitor Cocktail (Merck Poland, Warsaw, Poland) was used. Purity assays of isolated mitochondria were conducted according to previous reports [49,97]. Protein content was determined using a Bio-Rad Protein Assay (Bio-Rad Poland, Warsaw, Poland), with bovine serum albumin as a standard curve calibrator. The efficiency of organellar preparations (proteins per 100 g of cauliflower curds) varied from 0.9–3.5 mg for “*C*” and “*P*” and 0.1–1.9 mg for “*A*” cultivar.

### 4.4. Sample Preparation for the Two Dimensional Isoelectric Focusing/SDS Polyacrylamide Gel Electrophoresis (2D IEF/SDS-PAGE)

Proteins were extracted and precipitated overnight at −20 °C in a 10% solution of trichloroacetic acid in acetone containing 20 mM dithiothreitol (DTT) by the method of Staszak and Pawłowski [98]. After centrifugation (16,000× *g* for 5 min at 4 °C), resulting pellets were washed three times with 1 mL of acetone supplemented with 20 mM DTT. Samples were re-centrifuged after each washing, and resulting pellets were vacuum dried and then resuspended in lysis buffer (7 M urea, 2 M thiourea, 2% CHAPS, 1.5% DTT, 0.5% IPG buffer pH 4–7), and supplemented with Protease Inhibitor Cocktail (Roche, Basel, Switzerland) according to the manufacturer’s suggestions. Protein concentration in processed samples was determined using the Bradford assay [99].

### 4.5. 2D IEF/SDS-PAGE

All analyses were conducted at 25 °C with at least three biological replicas. Proteins (500 μg for CBB staining) were first separated according to their charge on rehydrated Immobiline DryStrip Gels (24 cm in length, containing linear gradient of pH 3–10) with rehydration buffer (6 M urea, 2 M thiourea, 2% CHAPS, 20 mM DTT, and 0.5% Pharmalyte, pH 4–7) on an Ettan IPGphor 3 IEF System (GE Healthcare, Uppsala, Sweden). The program for isoelectric focusing was applied according to the manufacturer’s suggestions. Strips were either stored at −80°C or they were directly treated for 10 min with equilibration solution I (6 M urea, 1.5 M Tris-HCl, pH 8.8, 30% glycerol, 2% SDS, and 1% DTT) and for the same time with equilibration in solution II (solution I supplemented with 2.5% 2-iodoacetamide, without DTT) and subjected to a second dimension run (SDS-PAGE). 

For SDS-PAGE, Ettan DALT 12.5% Precast Polyacrylamide Gels and an Ettan DALTsix Electrophoresis System (both from GE Healthcare) were used. Conditions for the run were as follows: 1 h at 80 V and 5 h at 500 V. Broad pI Kit, pH 3–10 (GE Healthcare) for protein spot pI calibration within 3.5–9.3, as well as PageRuler Prestained Protein Ladder (Thermo Scientific, Gdańsk, Poland) and LMW-SDS Marker Kit (GE Healthcare) for protein spot MW calibration were used. Resolved proteins were stained with colloidal CBB, which, in addition to visualization and quantification, also allowed for downstream MS analysis [100].

### 4.6. Proteome Analysis

Gels were scanned and evaluated using ImageMaster 2D Platinum v7.0 software (GE Healthcare). After spot detection, 2D gels (three gels from three independent biological samples) were aligned and matched, and normalized spot volumes were determined quantitatively. For each matched spot, the percent volume was calculated as the volume divided by the total volume of matched spots. Spots with variations in abundance were subjected to ANOVA, Tukey–Kramer HSD tests and contrast analysis (JMP software, SAS Institute, Cary, NC, USA) to assign spots that significantly varied (*p* < 0.05) in abundance for two factors: drought and cultivar, and their interactions (Table S2). An unpaired two-tailed Student’s *t*-test was used to assign significant variations in abundance (a given drought treatment vs. control) within analyzed cultivars (Section 4.10; Table S1). Proteins were subsequently identified by MS from spots that significantly varied in abundance.

### 4.7. Protein Identification by Mass Spectrometry (MS)

Gel spots were subjected to a standard “*in-gel* digestion” procedure in which proteins were reduced with 10 mM DTT (for 30 min at 56 °C), alkylated with 55 mM 2-iodoacetamide (45 min in the dark at room temperature) and digested overnight with trypsin (Promega, Madison, WI, USA) in 25 mM ammonium bicarbonate. The resulting peptides were eluted from the gel matrix with 0.1% trifluoroacetic acid in 2% acetonitrile. 

Peptide mixtures were analyzed by liquid chromatography coupled to a mass spectrometer in the Laboratory of Mass Spectrometry (Institute of Biochemistry and Biophysics, Polish Academy of Sciences, Warsaw, Poland). Samples were concentrated and desalted on a RP-C18 pre-column (nanoACQUITY Symmetry^®^ C18, Waters, Milford, MA, USA), and further peptide separation was achieved on a nano-Ultra Performance Liquid Chromatography (UPLC) RP-C18 column (BEH130 C18 column, 75 µm id, 250 mm long; Waters, Milford, MA, USA) of a nanoACQUITY UPLC system, using a linear 0–60% CAN gradient for 120 min in the presence of 0.05% formic acid with a flow rate of 150 nL min^−1^. The column outlet was directly coupled to the electrospray ionization (ESI) ion source of an Orbitrap Velos type mass spectrometer (Thermo Electron Corp., San Jose, CA, USA), working in the regime of data dependent MS to MS/MS switch. An electrospray voltage of 1.5 kV was used. A blank run preventing cross-contamination from previous samples preceded each analysis.

Proteins were identified using the Mascot search algorithm (Available online: www.matrixscience.com) against the NCBInr (Available online: www.ncbi.nig.gov) databases. Protein identification was performed using the Mascot search probability-based molecular weight search (MOWSE) score. The ion score was −10 × log(*P*), where *P* was the probability that the observed match was a random event. To avoid possible misidentifications resulting from the implementation of large datasets, as pointed out by Schmidt et al. [101], we were able to set the threshold of false positive rate. Peptides with a Mascot score exceeding the threshold value, corresponding to a <5% false positive rate as calculated by the Mascot procedure, were considered to be positively identified.

### 4.8. SDS-PAGE, Western Blotting, and Immunodetection of Proteins

Proteins resolved by one-dimensional SDS polyacrylamide gel electrophoresis (12% SDS-PAGE; [49]) were electroblotted in semidry conditions onto Immobilon-P membranes (Merck, Warsaw, Poland), using a TE77 PWR ECL Semi-Dry blotting apparatus (GE Healthcare Life Science Poland, Warsaw, Poland) and standard transfer buffer (20% methanol, 48 mM Tris, 39 mM glycine, 0.0375% SDS). Proteins resolved on 2D gels were electroblotted in semidry conditions using the same apparatus and alternative transfer buffer (10% methanol, 10 mM CAPS pH 11.0). Protein immunodetection was carried out with rabbit polyclonal antibodies directed against Mn-SOD (product. No. AS09 524, 1:5000 dilution), cyt. *c* (product No. AS08 343A, 1:5000), mitochondrial HSP70 (product No. AS08 347, 1:4000), SHMT1 (product. No. AS05 075, 1:10,000), GDC-H (product No. AS05 074, 1:4000), IDH (product No. AS06 203A, 1:4000), aconitase (product No. AS09 521, 1:5000; all antisera listed above from Agrisera, Vännäs, Sweden), PUMP (1:1000; [102,103]), dehydrin K-segment (1:1000, a gift of T.J. Close, University of California at Riverside, USA; [51]), dehydrin K-segment with N terminal cysteine on the synthetic peptide (product No. PLA-100, 1:1400; Stressgen, Victoria, BC, Canada), SK_3_-motif of *Solanum sogarandinum* DHN24 dehydrin (1:500, a gift of T. Rorat, Institute of Plant Genetics, Polish Academy of Sciences, Poznań, Poland; [104]) and mouse monoclonal antibodies directed against AOX (1:1000; [105]), and ATP1 (1:200; [106]; both antisera donated by T. Elthon, University of Lincoln, Lincoln, NE, USA). Immunoassay details were described previously [13,49]. Enhanced chemiluminescence (ECL) signals were quantified with Multi Gauge (v2.2, Fujifilm, Tokio, Japan). 

### 4.9. RNA Isolation and RT-qPCR 

Total RNA from cauliflower curds was extracted using an EZ-10 Spin Column Plant RNA Mini-Preps Kit (BioBasic, Markham, ON, Canada) according to the manufacturer’s protocol. Genomic DNA contaminants were removed by RQ1 DNase I free of RNase (Promega Poland, Warsaw, Poland). cDNA was synthesized using 1 μg of RNA, 0.2 μg of random hexamers mixture from HexaLabel DNA Labeling Kit (Thermo Scientific, Gdańsk, Poland) and 200 units of M-MLV reverse transcriptase (Promega Poland, Warsaw, Poland) in a 20 μL total volume for 1 h at 37 °C. After first strand synthesis, the reaction mixture was diluted with 10 mM Tris-HCl, pH 8.0 three or six times, and after normalization, aliquots of 1–2 μL were subjected to RT-quantitative PCR (RT-qPCR) using a Thermo Scientific Luminaris Color HiGreen High ROX qPCR Master Mix kit on an Applied Biosystems StepOnePlus Real-Time PCR System. The following profile was used: 5 min at 95 °C followed by 40 cycles of 20 s at 95 °C, 1 min at 60 °C, and finally, a melting step. The quality of qRT-PCR assays was verified by LinRegPCR (v. 2012.3, Heart Failure Research Center, Academic Medical Center, Amsterdam, The Netherlands). Outliers were manually removed. Two biological and at least three technical replicates were included.

Fragments of cauliflower cDNA for selected proteins were amplified using specific primers (Table S7); a 239 bp fragment of cauliflower actin1 (*ACT1*) cDNA was used as an internal standard. Amplicons were directly sequenced bi-directionally (Big Dye Terminator v. 3.1 Cycle Sequencing kit, Applied Biosystems Poland, Warsaw, Poland) on an ABI Prism 31-30 XL system (Applied Biosystems Poland, Warsaw, Poland) for sequence identity verification. In the case of *AOX1a* and *ACT1*, amplicons were additionally cloned to a pGEM T-Easy vector with the pGEM T-Easy Ligation System II (Promega) before sequencing. 

### 4.10. Statistical Analysis

All experiments were conducted in triplicate, unless otherwise indicated. Results of densitometric analyses (Section 2.4, Section 2.5, and Section 2.7), and 2D PAGE spot pattern alterations based on the spot volume (Section 2.2 and Section 2.3) are presented as means ± SE. An unpaired two-tailed Student’s *t*-test was used to identify significant differences; in particular, differences were considered to be statistically significant if *p* < 0.05 (*), *p* < 0.01 (**), or *p* < 0.001 (***). Significant correlations (Table S3) among variables were calculated using Spearman’s correlation coefficient (*p* < 0.05) with the help of STATISTICA 13.1 (StatSoft Poland, Kraków, Poland) software.

## 5. Conclusions

Results of our study that significantly broaden *Brassica* data suggest that plant mitochondria (across distinct cultivars) are actively engaged in the response to water deficit. Variations within the mitochondrial proteome of investigated cauliflower cultivars encompass major decreases in abundance; increased in abundance spots were specific to the intensity of the water deficit. Investigated proteomic variations coincided roughly with drought tolerance. Mitochondrial porin isoforms, ATP synthase subunit, DNA-binding proteins, heat shock proteins, components of energy-dissipating systems (AOX isoform 36 kDa, PUMP isoform 40 kDa) as well as dehydrin-like proteins (18 and 27 kDa) investigated in our study are among the best candidates for stress tolerance markers, highlighting diversity of drought responses within cauliflower mitochondria. Identification of dehydrin-specific tryptic peptides in several spots from 2D gels additionally indicates for the relevant participation of such proteins in acclimation to water deficit. 

The future study of the dynamic pattern of PTMs [30] among cauliflower drought-responsive proteins will use spectral data obtained from MS/MS peptide sequencing. Owing to the relevance of protein phosphorylation in the regulation of protein activity and stress-signaling pathways [107], we will pay special attention to such protein modification.

Alterations of transcripts for the stress-affected AOX isoform were largely unassociated with the proteomic ones, which is contrary to findings from our previous study on plant mitochondrial biogenesis in temperature stress and thermal recovery [13]. We suggest that the enhanced availability of *AOX1a* transcripts for translation may be an important regulatory point for the drought response of cauliflower mitochondria. Such results could be at least partially explained by Nakaminami et al. [108] findings on the imbalanced mRNA/protein pools and the altered pattern of translation initiation through stress acclimation and de-acclimation. Profiling of TF expression highlights valuable variations (additional to the ones at the protein level) in drought responses between stress-sensitive cultivars. 

Additional studies are required (1) to elucidate the impact of drought on transcript binding to ribosomes; (2) to investigate protein biosynthesis patterns [13] and mechanisms of increased selection of mRNA for translation; (3) to analyze participation of non-coding RNAs in the mitochondrial biogenesis (with emphasis on the cauliflower microtranscriptome targeting investigated mRNA); and (4) to characterize cauliflower genes with expression pattern regulated by the investigated CBF factors under drought.

## Supplementary Materials

Supplementary materials can be found at http://www.mdpi.com/1422-0067/19/4/1130/s1.

## Abbreviations

ACO	aconitase
AOX	alternative oxidase
ATP1, ATP2	mitochondrial ATP synthase subunit α or subunit β
CBB	Coomassie Brilliant Blue
CBF/DREB	C-repeat/dehydration-responsive element binding
CI, CII, CIV	respiratory complexes I, II and IV
CAPS	3-[(3-cholamidopropyl)dimethylammonio]-1-propanesulfonate
CHAPS	3-[(3-cholamidopropyl)dimethylammonio]-2-hydroxy-1-propanesulfonate
2D PAGE	two-dimensional gel electrophoresis
DHN	dehydrin
dlp	dehydrin-like protein
DTT	dithiothreitol
EDTA	ethylenediaminetetraacetic acid
EF	elongation factor
ERD	early response to dehydration
ERF	ETHYLENE RESPONSE FACTOR
FDH	formate dehydrogenase
GDC	glycine decarboxylase
HSP	heat shock protein
IDH	isocitrate dehydrogenase
IEF	isoelectrofocusing
LC-MS/MS	liquid chromatography-tandem mass spectrometry
LEA	late embryogenesis abundant
MDH	malate dehydrogenase
miRNA	microRNA
MPP	mitochondrial processing peptidase
OXPHOS	oxidative phosphorylation
P5CDH	Δ-1-pyrroline-5-carboxylate dehydrogenase
PDH	pyruvate dehydrogenase
PhR	photorespiration rate
PPFD	photosynthetic photon flux density
ProDH	proline dehydrogenase
PTMs	posttranslational protein modifications
PUMP	plant-uncoupling mitochondrial protein
R_d_	respiration in the light (day respiration) rate
R_n_	respiration in the dark (night respiration) rate
ROS	reactive oxygen species
R_T_	total respiration rate
RT-qPCR	reverse transcription quantitative PCR
RWC	relative water content
SDH	succinate dehydrogenase (complex II)
SHMT	serine hydroxymethyl aminotransferase
SOD	superoxide dismutase
TF(s)	transcription factor(s)
UPLC	nano-ultra performance liquid chromatography
VDAC	voltage-dependent anion channel

## References

[1] Zhang S., Chen F., Peng S., Ma W., Korpelainen H., Li C. (2010). Comparative physiological, ultrastructural and proteomic analyses reveal sexual differences in the responses of *Populus cathayana* under drought stress. Proteomics.

[2] Bogeat-Triboulot M.-B., Brosché M., Renaut J., Jouve L., Le Thiec D., Fayyaz P., Vinocur B., Witters E., Laukens K., Teichmann T. (2007). Gradual Soil Water Depletion Results in Reversible Changes of Gene Expression, Protein Profiles, Ecophysiology, and Growth Performance in *Populus euphratica*, a Poplar Growing in Arid Regions. Plant Physiol..

[3] Li Y.L., Stanghellini C. (2001). Analysis of the effect of EC and potential transpiration on vegetative growth of tomato. Sci. Hort..

[4] Chaves M.M. (2002). How Plants Cope with Water Stress in the Field? Photosynthesis and Growth. Ann. Bot..

[5] Flexas J., Medrano H. (2002). Drought-inhibition of Photosynthesis in C3 Plants: Stomatal and Non-stomatal Limitations Revisited. Ann. Bot..

[6] Alvarez S., Roy Choudhury S., Pandey S. (2014). Comparative Quantitative Proteomics Analysis of the ABA Response of Roots of Drought-Sensitive and Drought-Tolerant Wheat Varieties Identifies Proteomic Signatures of Drought Adaptability. J. Proteome Res..

[7] Johnová P., Skalák J., Saiz-Fernández I., Brzobohatý B. (2016). Plant responses to ambient temperature fluctuations and water-limiting conditions: A proteome-wide perspective. Biochim. Biophys. Acta.

[8] Atkin O.K., Macherel D. (2008). The crucial role of plant mitochondria in orchestrating drought tolerance. Ann. Bot..

[9] Salvato F., Havelund J.F., Chen M., Rao R.S.P., Rogowska-Wrzesinska A., Jensen O.N., Gang D.R., Thelen J.J., Møller I.M. (2014). The Potato Tuber Mitochondrial Proteome. Plant Physiol..

[10] Møller I.M. (2016). What is hot in plant mitochondria?. Physiol. Plant..

[11] Giegé P., Sweetlove L.J., Cognat V., Leaver C.J. (2005). Coordination of Nuclear and Mitochondrial Genome Expression during Mitochondrial Biogenesis in Arabidopsis. Plant Cell.

[12] Howell K.A., Cheng K., Murcha M.W., Jenkin L.E., Millar A.H., Whelan J. (2007). Oxygen Initiation of Respiration and Mitochondrial Biogenesis in Rice. J. Biol. Chem..

[13] Rurek M., Woyda-Ploszczyca A.M., Jarmuszkiewicz W. (2015). Biogenesis of mitochondria in cauliflower (*Brassica oleracea* var. *botrytis*) curds subjected to temperature stress and recovery involves regulation of the complexome, respiratory chain activity, organellar translation and ultrastructure. BBA Bioenerg..

[14] Taylor N.L., Tan Y.-F., Jacoby R.P., Millar A.H. (2009). Abiotic environmental stress induced changes in the *Arabidopsis thaliana* chloroplast, mitochondria and peroxisome proteomes. J. Proteom..

[15] Ali G.M., Komatsu S. (2006). Proteomic Analysis of Rice Leaf Sheath during Drought Stress. J. Proteome Res..

[16] Aranjuelo I., Molero G., Erice G., Avice J.C., Nogués S. (2011). Plant physiology and proteomics reveals the leaf response to drought in alfalfa (*Medicago sativa* L.). J. Exp. Bot..

[17] Kosová K., Vítámvás P., Urban M.O., Klíma M., Roy A., Prášil I.T. (2015). Biological Networks Underlying Abiotic Stress Tolerance in Temperate Crops--A Proteomic Perspective. Int. J. Mol. Sci..

[18] Ndimba B.K., Chivasa S., Simon W.J., Slabas A.R. (2005). Identification of *Arabidopsis* salt and osmotic stress responsive proteins using two-dimensional difference gel electrophoresis and mass spectrometry. Proteomics.

[19] Taylor N.L., Heazlewood J.L., Day D.A., Millar A.H. (2005). Differential Impact of Environmental Stresses on the Pea Mitochondrial Proteome. Mol. Cell Proteom..

[20] Bies-Ethève N., Gaubier-Comella P., Debures A., Lasserre E., Jobet E., Raynal M., Cooke R., Delseny M. (2008). Inventory, evolution and expression profiling diversity of the LEA (late embryogenesis abundant) protein gene family in *Arabidopsis thaliana*. Plant Mol. Biol..

[21] Taylor N.L., Day D.A., Millar A.H. (2002). Environmental Stress Causes Oxidative Damage to Plant Mitochondria Leading to Inhibition of Glycine Decarboxylase. J. Biol. Chem..

[22] Vanlerberghe G.C. (2013). Alternative Oxidase: A Mitochondrial Respiratory Pathway to Maintain Metabolic and Signaling Homeostasis During Abiotic and Biotic Stress in Plants. Int. J. Mol. Sci..

[23] Zadražnik T., Hollung K., Egge-Jacobsen W., Meglič V., Šuštar-Vozlič J. (2013). Differential proteomic analysis of drought stress response in leaves of common bean (*Phaseolus vulgaris* L.). J. Proteom..

[24] Yu C., Wang L., Xu S., Zeng Y., He C., Chen C., Huang W., Zhu Y., Hu J. (2015). Mitochondrial ORFH79 is Essential for Drought and Salt Tolerance in Rice. Plant Cell Physiol..

[25] Reddy P.S., Kavi Kishor P.B., Seiler C., Kuhlmann M., Eschen-Lippold L., Lee J., Reddy M.K., Sreenivasulu N. (2014). Unraveling Regulation of the Small Heat Shock Proteins by the Heat Shock Factor HvHsfB2c in Barley: Its Implications in Drought Stress Response and Seed Development. PLoS ONE.

[26] Wu G., Wilen R.W., Robertson A.J., Gusta L.V. (1999). Isolation, Chromosomal Localization, and Differential Expression of Mitochondrial Manganese Superoxide Dismutase and Chloroplastic Copper/Zinc Superoxide Dismutase Genes in Wheat. Plant Physiol..

[27] Huseynova I.M., Aliyeva D.R., Aliyev J.A. (2014). Subcellular localization and responses of superoxide dismutase isoforms in local wheat varieties subjected to continuous soil drought. Plant Physiol. Biochem..

[28] Dalal M., Tayal D., Chinnusamy V., Bansal K.C. (2009). Abiotic stress and ABA-inducible Group 4 LEA from *Brassica napus* plays a key role in salt and drought tolerance. J. Biotechnol..

[29] Efeoğlu B., Ekmekçi Y., Çiçek N. (2009). Physiological responses of three maize cultivars to drought stress and recovery. S. Afr. J. Bot..

[30] Koh J., Chen G., Yoo M.-J., Zhu N., Dufresne D., Erickson J.E., Shao H., Chen S. (2015). Comparative Proteomic Analysis of *Brassica napus* in Response to Drought Stress. J. Proteome Res..

[31] Zhang J., Mason A.S., Wu J., Liu S., Zhang X., Luo T., Redden R., Batley J., Hu L., Yan G. (2015). Identification of Putative Candidate Genes for Water Stress Tolerance in Canola (*Brassica napus*). Front. Plant Sci..

[32] Kwon S.-W., Kim M., Kim H., Lee J. (2016). Shotgun Quantitative Proteomic Analysis of Proteins Responding to Drought Stress in *Brassica rapa* L. (Inbred Line “Chiifu”). Int. J. Genom..

[33] Wong C.E., Li Y., Whitty B.R., Díaz-Camino C., Akhter S.R., Brandle J.E., Golding G.B., Weretilnyk E.A., Moffatt B.A., Griffith M. (2005). Expressed sequence tags from the Yukon ecotype of *Thellungiella* reveal that gene expression in response to cold, drought and salinity shows little overlap. Plant Mol. Biol..

[34] Li Z., Zhao L., Kai G., Yu S., Cao Y., Pang Y., Sun X., Tang K. (2004). Cloning and expression analysis of a water stress-induced gene from *Brassica oleracea*. Plant Physiol. Biochem..

[35] Mohammadi P.P., Moieni A., Komatsu S. (2012). Comparative proteome analysis of drought-sensitive and drought-tolerant rapeseed roots and their hybrid F1 line under drought stress. Amino Acids.

[36] De Mezer M., Turska-Taraska A., Kaczmarek Z., Glowacka K., Swarcewicz B., Rorat T. (2014). Differential physiological and molecular response of barley genotypes to water deficit. Plant Physiol. Biochem..

[37] Das A., Mukhopadhyay M., Sarkar B., Saha D., Mondal T.K. (2015). Influence of drought stress on cellular ultrastructure and antioxidant system in tea cultivars with different drought sensitivities. J. Environ. Biol..

[38] Urban M.O., Vašek J., Klíma M., Krtková J., Kosová K., Prášil I.T., Vítámvás P. (2017). Proteomic and physiological approach reveals drought-induced changes in rapeseeds: Water-saver and water-spender strategy. J. Proteom..

[39] Vincent D., Ergül A., Bohlman M.C., Tattersall E.A.R., Tillett R.L., Wheatley M.D., Woolsey R., Quilici D.R., Joets J., Schlauch K. (2007). Proteomic analysis reveals differences between *Vitis vinifera* L. cv. Chardonnay and cv. Cabernet Sauvignon and their responses to water deficit and salinity. J. Exp. Bot..

[40] Bonhomme L., Monclus R., Vincent D., Carpin S., Lomenech A.-M., Plomion C., Brignolas F., Morabito D. (2009). Leaf proteome analysis of eight *Populus*
*xeuramericana* genotypes: Genetic variation in drought response and in water-use efficiency involves photosynthesis-related proteins. Proteomics.

[41] Ford K.L., Cassin A., Bacic A. (2011). Quantitative Proteomic Analysis of Wheat Cultivars with Differing Drought Stress Tolerance. Front. Plant Sci..

[42] Ge P., Ma C., Wang S., Gao L., Li X., Guo G., Ma W., Yan Y. (2012). Comparative proteomic analysis of grain development in two spring wheat varieties under drought stress. Anal. Bioanal. Chem..

[43] Ashoub A., Beckhaus T., Berberich T., Karas M., Brüggemann W. (2013). Comparative analysis of barley leaf proteome as affected by drought stress. Planta.

[44] Budak H., Akpinar B.A., Unver T., Turktas M. (2013). Proteome changes in wild and modern wheat leaves upon drought stress by two-dimensional electrophoresis and nanoLC-ESI-MS/MS. Plant Mol. Biol..

[45] Kausar R., Arshad M., Shahzad A., Komatsu S. (2013). Proteomics analysis of sensitive and tolerant barley genotypes under drought stress. Amino Acids.

[46] Oliveira T.M., da Silva F.R., Bonatto D., Neves D.M., Morillon R., Maserti B.E., Filho M.A.C., Costa M., Pirovani C.P., Gesteira A.S. (2015). Comparative study of the protein profiles of Sunki mandarin and Rangpur lime plants in response to water deficit. BMC Plant Biol..

[47] Vítámvás P., Urban M.O., Škodáček Z., Kosová K., Pitelková I., Vítámvás J., Renaut J., Prášil I.T. (2015). Quantitative analysis of proteome extracted from barley crowns grown under different drought conditions. Front. Plant Sci..

[48] Cheng L., Wang Y., He Q., Li H., Zhang X., Zhang F. (2016). Comparative proteomics illustrates the complexity of drought resistance mechanisms in two wheat (*Triticum aestivum* L.) cultivars under dehydration and rehydration. BMC Plant Biol..

[49] Rurek M. (2010). Diverse accumulation of several dehydrin-like proteins in cauliflower (*Brassica oleracea* var. *botrytis*), *Arabidopsis thaliana* and yellow lupin (*Lupinus luteus*) mitochondria under cold and heat stress. BMC Plant Biol..

[50] Katari M.S., Nowicki S.D., Aceituno F.F., Nero D., Kelfer J., Thompson L.P., Cabello J.M., Davidson R.S., Goldberg A.P., Shasha D.E. (2010). VirtualPlant: A software platform to support systems biology research. Plant Physiol..

[51] Close T.J., Fenton R.D., Moonan F. (1993). A view of plant dehydrins using antibodies specific to the carboxy terminal peptide. Plant Mol. Biol..

[52] Krzesiński W., Kałużewicz A., Frąszczak B., Zaworska A., Lisiecka J. (2016). Cauliflower’s response to drought stress. Nauka Przyroda Technol..

[53] Voss I., Sunil B., Scheibe R., Raghavendra A.S. (2013). Emerging concept for the role of photorespiration as an important part of abiotic stress response. Plant Biol. (Stuttg.).

[54] Kim J., van Iersel M.W. (2011). Slowly developing drought stress increases photosynthetic acclimation of *Catharanthus roseus*. Physiol. Plant..

[55] Haupt-Herting S., Klug K., Fock H.P. (2001). A New Approach to Measure Gross CO_2_ Fluxes in Leaves. Gross CO_2_ Assimilation, Photorespiration, and Mitochondrial Respiration in the Light in Tomato under Drought Stress. Plant Physiol..

[56] Campos H., Trejo C., Peña-Valdivia C.B., García-Nava R., Conde-Martínez F.V., Cruz-Ortega M.R. (2014). Stomatal and non-stomatal limitations of bell pepper (*Capsicum annuum* L.) plants under water stress and re-watering: Delayed restoration of photosynthesis during recovery. Environ. Exp. Bot..

[57] Sperlich D., Barbeta A., Ogaya R., Sabaté S., Peñuelas J. (2016). Balance between carbon gain and loss under long-term drought: impacts on foliar respiration and photosynthesis in *Quercus ilex* L.. J. Exp. Bot..

[58] Vassileva V., Simova-Stoilova L., Demirevska K., Feller U. (2009). Variety-specific response of wheat (*Triticum aestivum* L.) leaf mitochondria to drought stress. J. Plant Res..

[59] Chastain D.R., Snider J.L., Collins G.D., Perry C.D., Whitaker J., Byrd S.A. (2014). Water deficit in field-grown *Gossypium hirsutum* primarily limits net photosynthesis by decreasing stomatal conductance, increasing photorespiration, and increasing the ratio of dark respiration to gross photosynthesis. J. Plant Physiol..

[60] Liu C., Wang Y., Pan K., Wang Q., Liang J., Jin Y., Tariq A. (2017). The Synergistic Responses of Different Photoprotective Pathways in Dwarf Bamboo (*Fargesia rufa*) to Drought and Subsequent Rewatering. Front. Plant Sci..

[61] Abogadallah G.M. (2011). Differential regulation of photorespiratory gene expression by moderate and severe salt and drought stress in relation to oxidative stress. Plant Sci..

[62] Lima Neto M.C., Cerqueira J.V.A., da Cunha J.R., Ribeiro R.V., Silveira J.A.G. (2017). Cyclic electron flow, NPQ and photorespiration are crucial for the establishment of young plants of *Ricinus communis* and *Jatropha curcas* exposed to drought. Plant Biol. (Stuttg.).

[63] Zhou S., Li M., Guan Q., Liu F., Zhang S., Chen W., Yin L., Qin Y., Ma F. (2015). Physiological and proteome analysis suggest critical roles for the photosynthetic system for high water-use efficiency under drought stress in *Malus*. Plant Sci..

[64] Chen L., Ren F., Zhong H., Feng Y., Jiang W., Li X. (2010). Identification and expression analysis of genes in response to high-salinity and drought stresses in *Brassica napus*. Acta Biochim. Biophys. Sin. (Shanghai).

[65] He C.Y., Zhang G.Y., Zhang J.G., Duan A.G., Luo H.M. (2016). Physiological, biochemical, and proteome profiling reveals key pathways underlying the drought stress responses of *Hippophae rhamnoides*. Proteomics.

[66] Bernard D.G., Cheng Y., Zhao Y., Balk J. (2009). An Allelic Mutant Series of *ATM3* Reveals Its Key Role in the Biogenesis of Cytosolic Iron-Sulfur Proteins in Arabidopsis. Plant Physiol..

[67] Landi S., Hausman J.-F., Guerriero G., Esposito S. (2017). *Poaceae* vs. Abiotic Stress: Focus on Drought and Salt Stress, Recent Insights and Perspectives. Front. Plant Sci..

[68] Caruso G., Cavaliere C., Foglia P., Gubbiotti R., Samperi R., Laganà A. (2009). Analysis of drought responsive proteins in wheat (*Triticum durum*) by 2D-PAGE and MALDI-TOF mass spectrometry. Plant Sci..

[69] Hamilton C.A., Good A.G., Taylor G.J. (2001). Induction of Vacuolar ATPase and Mitochondrial ATP Synthase By Aluminum in an Aluminum-Resistant Cultivar of Wheat. Plant Physiol..

[70] Moghadam A.A., Taghavi S.M., Niazi A., Djavaheri M., Ebrahimie E. (2012). Isolation and in silico functional analysis of *MtATP6*, a 6-kDa subunit of mitochondrial F_1_F_0_-ATP synthase, in response to abiotic stress. Genet. Mol. Res..

[71] Li C.-L., Wang M., Ma X.-Y., Zhang W. (2014). NRGA1, a Putative Mitochondrial Pyruvate Carrier, Mediates ABA Regulation of Guard Cell Ion Channels and Drought Stress Responses in *Arabidopsis*. Mol. Plant.

[72] Wang M., Ma X., Shen J., Li C., Zhang W. (2014). The ongoing story: The mitochondria pyruvate carrier 1 in plant stress response in Arabidopsis. Plant Signal. Behav..

[73] Taylor N.L., Rudhe C., Hulett J.M., Lithgow T., Glaser E., Day D.A., Millar A.H., Whelan J. (2003). Environmental stresses inhibit and stimulate different protein import pathways in plant mitochondria. FEBS Lett..

[74] Riccardi F., Gazeau P., de Vienne D., Zivy M. (1998). Protein Changes in Response to Progressive Water Deficit in Maize: Quantitative Variation and Polypeptide Identification. Plant Physiol..

[75] Kaouthar F., Ameny F.-K., Yosra K., Walid S., Ali G., Faiçal B. (2016). Responses of transgenic *Arabidopsis* plants and recombinant yeast cells expressing a novel durum wheat manganese superoxide dismutase *TdMnSOD* to various abiotic stresses. J. Plant Physiol..

[76] Dahal K., Wang J., Martyn G.D., Rahimy F., Vanlerberghe G.C. (2014). Mitochondrial Alternative Oxidase Maintains Respiration and Preserves Photosynthetic Capacity during Moderate Drought in *Nicotiana tabacum*. Plant Physiol..

[77] Galle A., Florez-Sarasa I., Thameur A., de Paepe R., Flexas J., Ribas-Carbo M. (2010). Effects of drought stress and subsequent rewatering on photosynthetic and respiratory pathways in *Nicotiana sylvestris* wild type and the mitochondrial complex I-deficient CMSII mutant. J. Exp. Bot..

[78] Hincha D.K., Thalhammer A. (2012). LEA proteins: IDPs with versatile functions in cellular dehydration tolerance. Biochem. Soc. Trans..

[79] Borovskii G.B., Stupnikova I.V., Antipina A.I., Vladimirova S.V., Voinikov V.K. (2002). Accumulation of dehydrin-like proteins in the mitochondria of cereals in response to cold, freezing, drought and ABA treatment. BMC Plant Biol..

[80] Grelet J., Benamar A., Teyssier E., Avelange-Macherel M.-H., Grunwald D., Macherel D. (2005). Identification in Pea Seed Mitochondria of a Late-Embryogenesis Abundant Protein Able to Protect Enzymes from Drying. Plant Physiol..

[81] Boswell L.C., Moore D.S., Hand S.C. (2014). Quantification of cellular protein expression and molecular features of group 3 LEA proteins from embryos of *Artemia franciscana*. Cell Stress Chaperones.

[82] Boswell L.C., Hand S.C. (2014). Intracellular localization of group 3 LEA proteins in embryos of *Artemia franciscana*. Tissue Cell.

[83] Clifton R., Millar A.H., Whelan J. (2006). Alternative oxidases in Arabidopsis: A comparative analysis of differential expression in the gene family provides new insights into function of non-phosphorylating bypasses. Biochim. Biophys. Acta.

[84] Das S., Ferlito M., Kent O.A., Fox-Talbot K., Wang R., Liu D., Raghavachari N., Yang Y., Wheelan S.J., Murphy E. (2012). Nuclear miRNA Regulates the Mitochondrial Genome in the Heart. Circ. Res..

[85] Leung A.K.L. (2015). The Whereabouts of microRNA Actions: Cytoplasm and Beyond. Trends Cell Biol..

[86] Ro S., Ma H.-Y., Park C., Ortogero N., Song R., Hennig G.W., Zheng H., Lin Y.-M., Moro L., Hsieh J.-T. (2013). The mitochondrial genome encodes abundant small noncoding RNAs. Cell Res..

[87] Rurek M. (2016). Participation of non-coding RNAs in plant organelle biogenesis. Acta Biochim. Pol..

[88] Dai X., Zhao P.X. (2011). psRNATarget: A plant small RNA target analysis server. Nucleic Acids Res..

[89] Wu H., Wu X., Li Z., Duan L., Zhang M. (2012). Physiological Evaluation of Drought Stress Tolerance and Recovery in Cauliflower (*Brassica oleracea* L.) Seedlings Treated with Methyl Jasmonate and Coronatine. J. Plant Growth Regul..

[90] Kiyosue T., Yamaguchi-Shinozaki K., Shinozaki K. (1994). Characterization of two cDNAs (ERD10 and ERD14) corresponding to genes that respond rapidly to dehydration stress in Arabidopsis thaliana. Plant Cell Physiol..

[91] Nylander M., Svensson J., Palva E.T., Welin B.V. (2001). Stress-induced accumulation and tissue-specific localization of dehydrins in Arabidopsis thaliana. Plant Mol. Biol..

[92] Mizoi J., Shinozaki K., Yamaguchi-Shinozaki K. (2012). AP2/ERF family transcription factors in plant abiotic stress responses. Biochim. Biophys. Acta.

[93] Thamilarasan S.K., Park J.-I., Jung H.-J., Nou I.-S. (2014). Genome-wide analysis of the distribution of AP2/ERF transcription factors reveals duplication and CBFs genes elucidate their potential function in *Brassica oleracea*. BMC Genom..

[94] Haake V., Cook D., Riechmann J., Pineda O., Thomashow M.F., Zhang J.Z. (2002). Transcription Factor CBF4 Is a Regulator of Drought Adaptation in Arabidopsis. Plant Physiol..

[95] Mohavedi S., Tabatabaei B.E.S., Alizade H., Ghobadi C., Yamchi A., Khaksar G. (2012). Constitutive expression of *Arabidopsis DREB1B* in transgenic potato enhances drought and freezing tolerance. Biol. Plant..

[96] Laisk A. (1977). Kinetics of Photosynthesis and Photorespiration in C_3_-Plants.

[97] Pawlowski T., Rurek M., Janicka S., Raczynska K.D., Augustyniak H. (2005). Preliminary analysis of the cauliflower mitochondrial proteome. Acta Physiol. Plant..

[98] Staszak A., Pawłowski T. (2014). Proteomic Analysis of Embryogenesis and the Acquisition of Seed Dormancy in Norway Maple (*Acer platanoides* L.). Int. J. Mol. Sci..

[99] Ramagli L.S., Rodriguez L.V. (1985). Quantitation of microgram amounts of protein in two-dimensional polyacrylamide gel electrophoresis sample buffer. Electrophoresis.

[100] Neuhoff V., Arold N., Taube D., Ehrhardt W. (1988). Improved staining of proteins in polyacrylamide gels including isoelectric focusing gels with clear background at nanogram sensitivity using Coomassie Brilliant Blue G-250 and R-250. Electrophoresis.

[101] Schmidt U.G., Endler A., Schelbert S., Brunner A., Schnell M., Neuhaus H.E., Marty-Mazars D., Marty F., Baginsky S., Martinoia E. (2007). Novel Tonoplast Transporters Identified Using a Proteomic Approach with Vacuoles Isolated from Cauliflower Buds. Plant Physiol..

[102] Nantes I.L., Fagian M.M., Catisti R., Arruda P., Maia I.G., Vercesi A.E. (1999). Low temperature and aging-promoted expression of PUMP in potato tuber mitochondria. FEBS Lett..

[103] Ježek P., Žáčková M., Košařová J., Rodrigues E.T., Madeira V.M., Vicente J.A. (2000). Occurrence of plant-uncoupling mitochondrial protein (PUMP) in diverse organs and tissues of several plants. J. Bioenerg. Biomembr..

[104] Rorat T., Szabala B.M., Grygorowicz W.J., Wojtowicz B., Yin Z., Rey P. (2006). Expression of SK_3_-type dehydrin in transporting organs is associated with cold acclimation in *Solanum* species. Planta.

[105] Elthon T.E., Nickels R.L., McIntosh L. (1989). Monoclonal Antibodies to the Alternative Oxidase of Higher Plant Mitochondria. Plant Physiol..

[106] Luethy M.H., Horak A., Elthon T.E. (1993). Monoclonal Antibodies to the α- and β-Subunits of the Plant Mitochondrial F_1_-ATPase. Plant Physiol..

[107] Havelund J.F., Thelen J.J., Møller I.M. (2013). Biochemistry, proteomics, and phosphoproteomics of plant mitochondria from non-photosynthetic cells. Front. Plant Sci..

[108] Nakaminami K., Matsui A., Nakagami H., Minami A., Nomura Y., Tanaka M., Morosawa T., Ishida J., Takahashi S., Uemura M. (2014). Analysis of Differential Expression Patterns of mRNA and Protein During Cold-Acclimation and De-Acclimation in *Arabidopsis*. Mol. Cell. Proteom..

