# Supplementary material for: Mitochondrial Biogenesis in Diverse Cauliflower Cultivars under Mild and Severe Drought. Impaired Coordination of Selected Transcript and Proteomic Responses, and Regulation of Various Multifunctional Proteins
Source: Int J Mol Sci. 2018 Apr 10;19(4):1130. doi: 10.3390/ijms19041130 (PMC5979313; doi:10.3390/ijms19041130)
Supplement: Supplementary file 1 [file ijms-19-01130-s001.zip › Figure S1.pptx]

## Slide 1
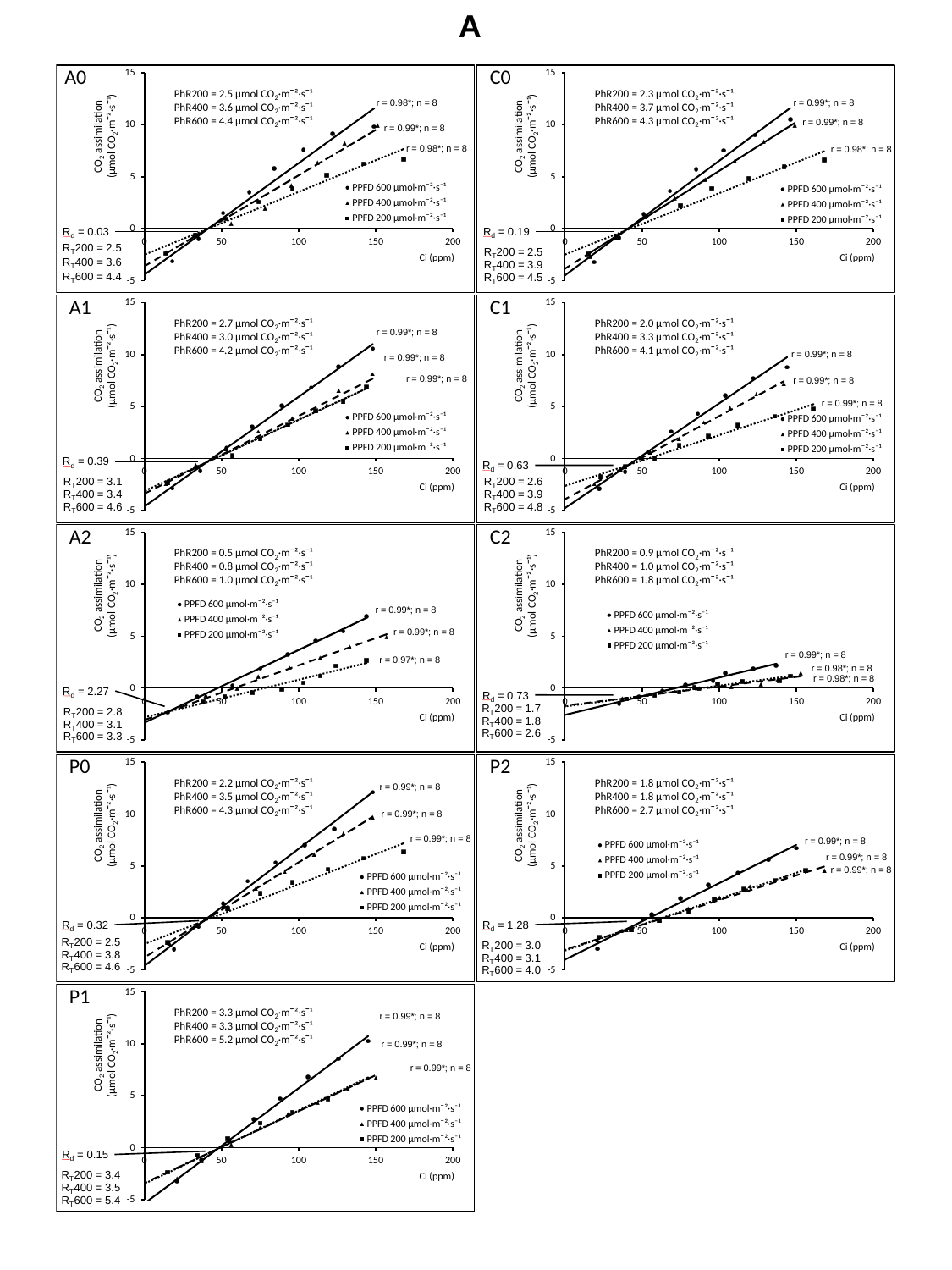

A

## Slide 2
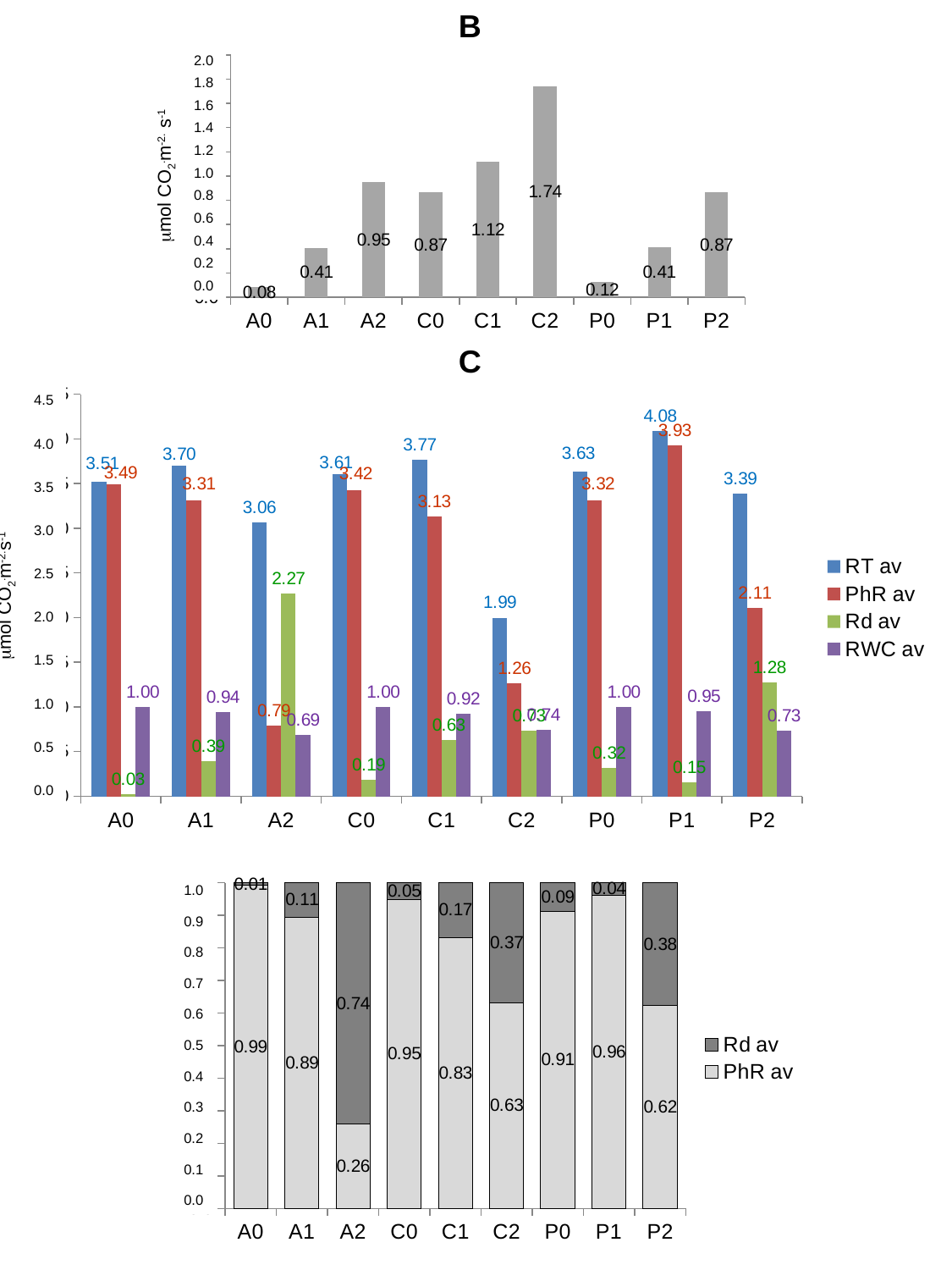

B
### Chart
| Category | |
|---|---|
| A0 | 0.08181818181818182 |
| A1 | 0.40818181818181826 |
| A2 | 0.9536363636363636 |
| C0 | 0.868181818181818 |
| C1 | 1.12 |
| C2 | 1.7372727272727275 |
| P0 | 0.12363636363636364 |
| P1 | 0.41272727272727266 |
| P2 | 0.8690909090909092 |mmol CO2.m-2. s-1
C
### Chart
| Category | RT av | PhR av | Rd av | RWC av |
|---|---|---|---|---|
| A0 | 3.51492658184886 | 3.48911559302493 | 0.0258109888239318 | 1.0 |
| A1 | 3.70415669574317 | 3.31382999827325 | 0.390326697469918 | 0.94 |
| A2 | 3.06546136044413 | 0.793708575862828 | 2.27175278458131 | 0.69 |
| C0 | 3.60891522082524 | 3.42343874418491 | 0.185476476640338 | 1.0 |
| C1 | 3.7672070766505 | 3.13449057330234 | 0.632716503348164 | 0.92 |
| C2 | 1.99812846200845 | 1.26315473717759 | 0.734973724830858 | 0.74 |
| P0 | 3.63220896210026 | 3.31562338403046 | 0.316585578069802 | 1.0 |
| P1 | 4.0837203393409 | 3.92922066825728 | 0.154499671083621 | 0.95 |
| P2 | 3.38635735982635 | 2.1086990248788 | 1.27765833494754 | 0.73 |mmol CO2.m-2.s-1
### Chart
| Category | PhR av | Rd av |
|---|---|---|
| A0 | 0.9926567488045928 | 0.007343251195407659 |
| A1 | 0.8946246799120338 | 0.10537532008796573 |
| A2 | 0.2589197783095964 | 0.7410802216904061 |
| C0 | 0.9486060310948736 | 0.051393968905128685 |
| C1 | 0.8320462638569045 | 0.1679537361430965 |
| C2 | 0.6321689326760854 | 0.3678310673239135 |
| P0 | 0.9128393819372276 | 0.08716061806277303 |
| P1 | 0.9621669315610981 | 0.037833068438902155 |
| P2 | 0.6227042219155904 | 0.3772957780844068 |2.0
1.8
1.6
1.4
1.2
1.0
0.8
0.6
0.4
0.2
0.0
4.5
4.0
3.5
3.0
2.5
2.0
1.5
1.0
0.5
0.0
1.0
0.9
0.8
0.7
0.6
0.5
0.4
0.3
0.2
0.1
0.0

## Slide 3
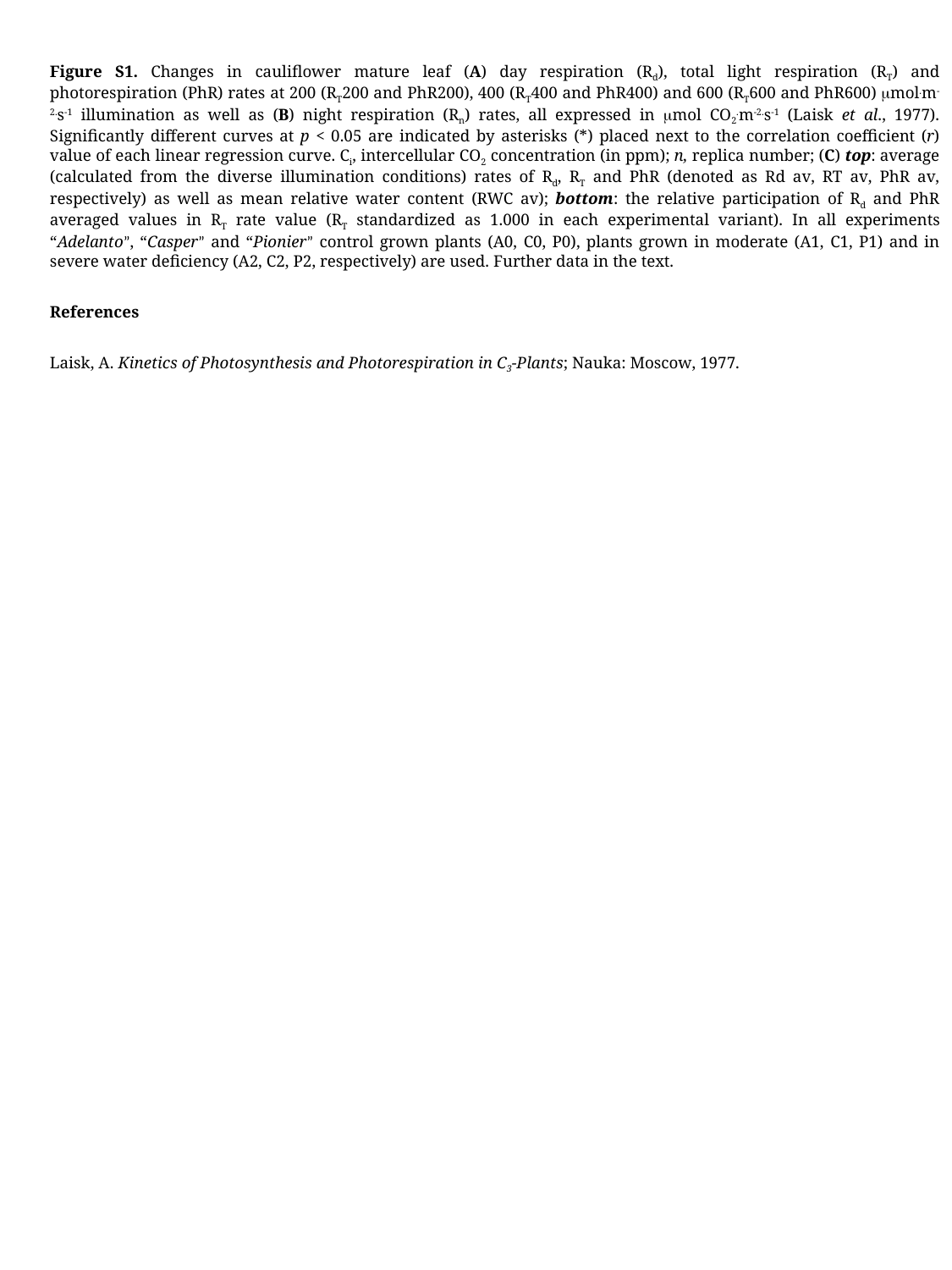

Figure S1. Changes in cauliflower mature leaf (A) day respiration (Rd), total light respiration (RT) and photorespiration (PhR) rates at 200 (RT200 and PhR200), 400 (RT400 and PhR400) and 600 (RT600 and PhR600) mmol.m-2.s-1 illumination as well as (B) night respiration (Rn) rates, all expressed in mmol CO2.m-2.s-1 (Laisk et al., 1977). Significantly different curves at p < 0.05 are indicated by asterisks (*) placed next to the correlation coefficient (r) value of each linear regression curve. Ci, intercellular CO2 concentration (in ppm); n, replica number; (C) top: average (calculated from the diverse illumination conditions) rates of Rd, RT and PhR (denoted as Rd av, RT av, PhR av, respectively) as well as mean relative water content (RWC av); bottom: the relative participation of Rd and PhR averaged values in RT rate value (RT standardized as 1.000 in each experimental variant). In all experiments “Adelanto”, “Casper” and “Pionier” control grown plants (A0, C0, P0), plants grown in moderate (A1, C1, P1) and in severe water deficiency (A2, C2, P2, respectively) are used. Further data in the text.
References
Laisk, A. Kinetics of Photosynthesis and Photorespiration in C3-Plants; Nauka: Moscow, 1977.
