# Supplementary material for: Mitochondrial Biogenesis in Diverse Cauliflower Cultivars under Mild and Severe Drought. Impaired Coordination of Selected Transcript and Proteomic Responses, and Regulation of Various Multifunctional Proteins
Source: Int J Mol Sci. 2018 Apr 10;19(4):1130. doi: 10.3390/ijms19041130 (PMC5979313; doi:10.3390/ijms19041130)
Supplement: Supplementary file 1 [file ijms-19-01130-s001.zip › Figure S2.pptx]

## Slide 1
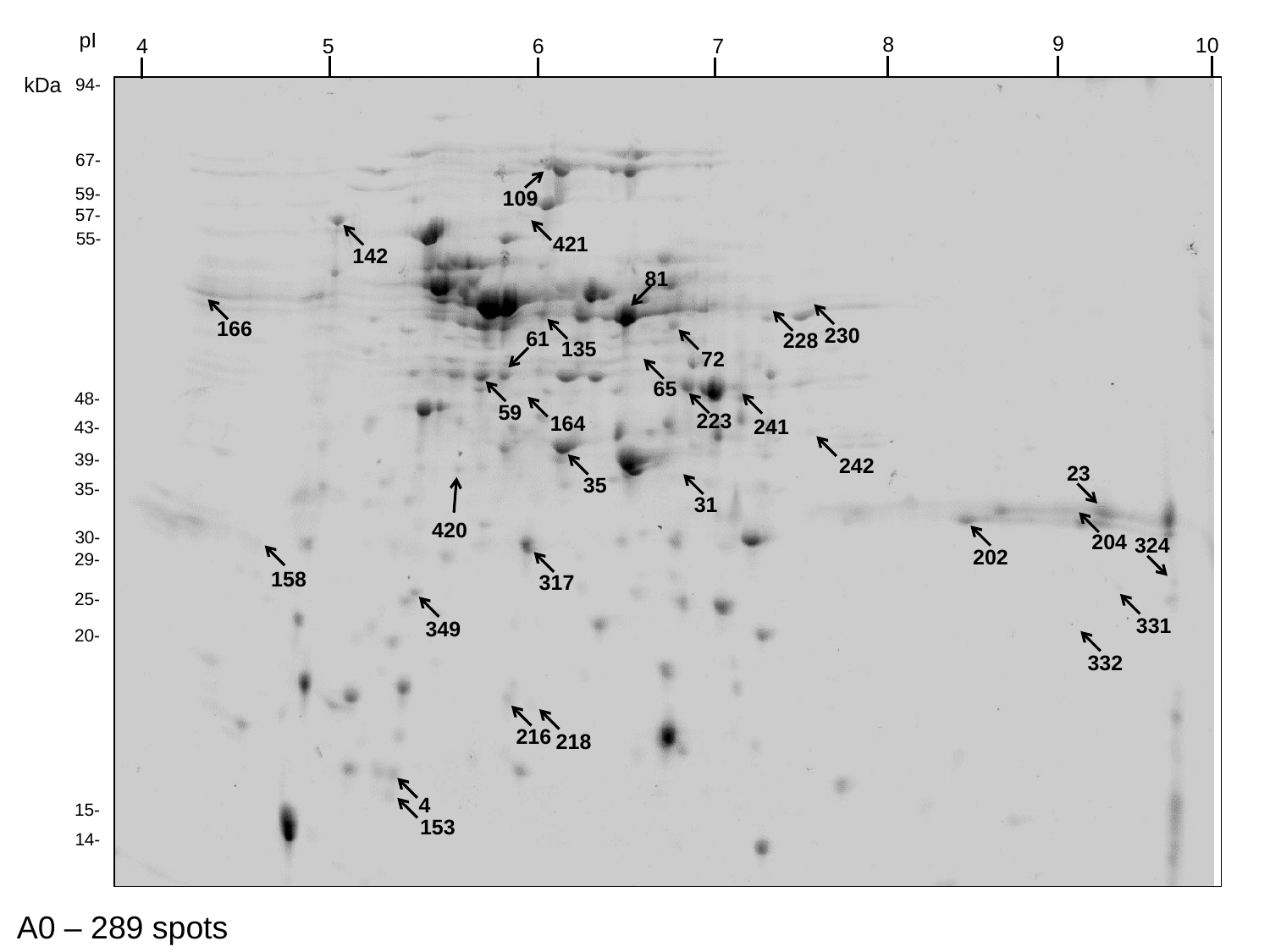

pI
9
8
10
7
5
4
6
kDa
94-
67-
59-
57-
109
55-
421
142
81
166
230
61
228
135
72
65
48-
59
223
164
241
43-
39-
242
23
35
35-
31
420
30-
204
324
202
29-
158
317
25-
331
349
20-
332
216
218
4
15-
153
14-
A0 – 289 spots

## Slide 2
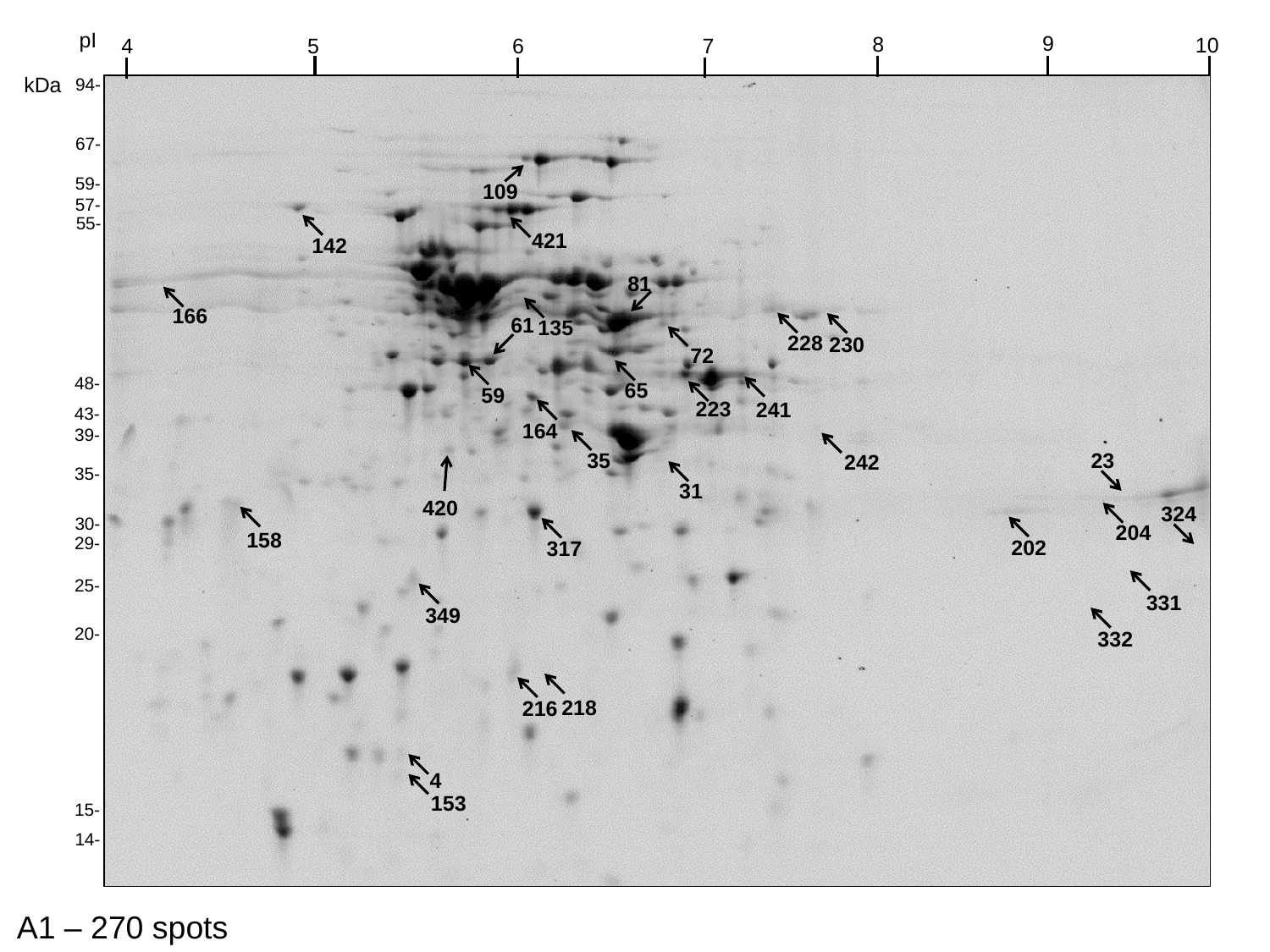

pI
9
8
10
7
5
4
6
kDa
94-
67-
59-
57-
109
55-
421
142
81
166
61
135
228
230
72
48-
65
59
223
241
43-
164
39-
35
23
242
35-
31
420
324
30-
204
158
29-
202
317
25-
331
349
20-
332
218
216
4
153
15-
14-
A1 – 270 spots

## Slide 3
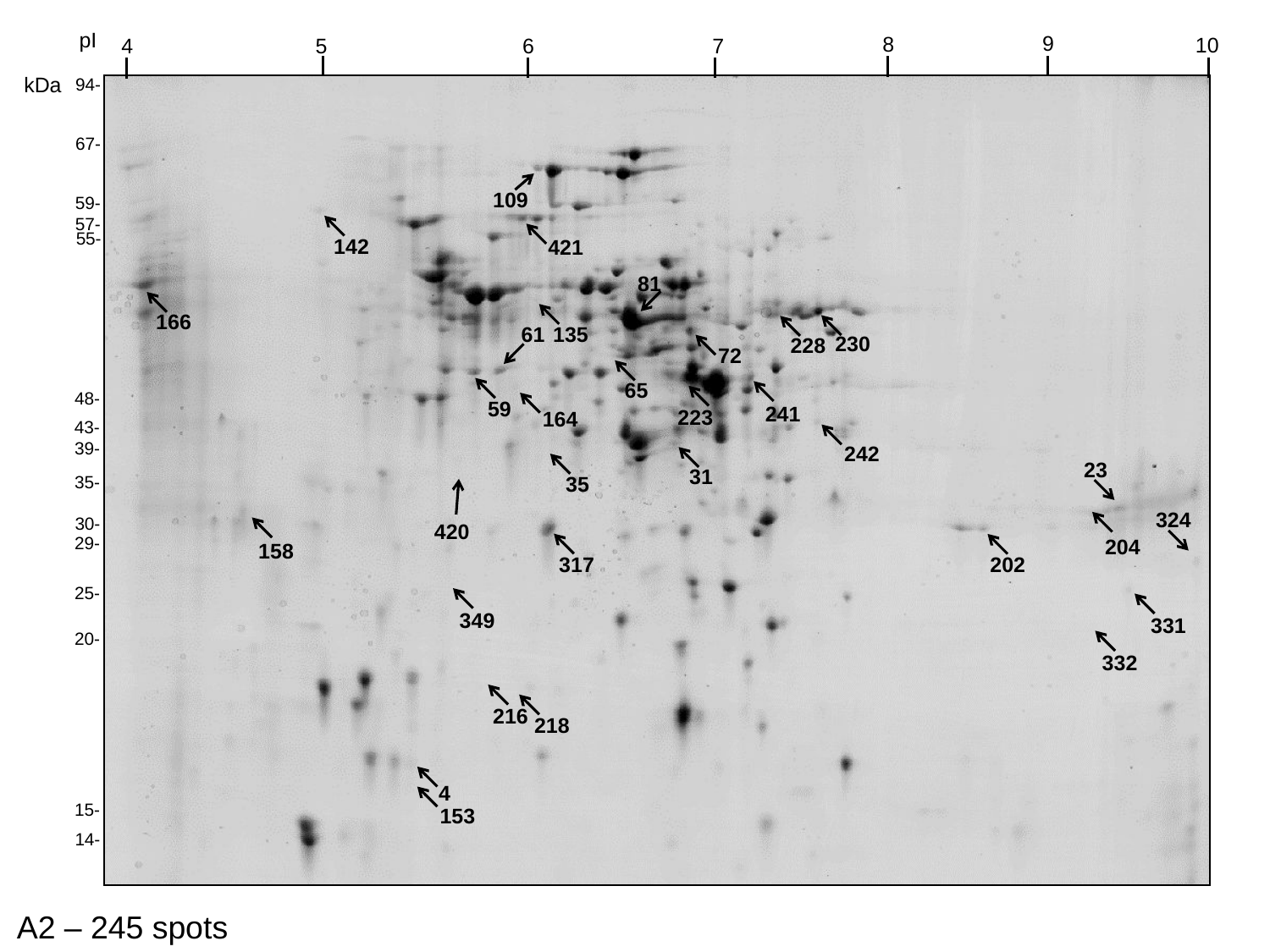

pI
9
8
10
7
5
4
6
kDa
94-
67-
109
59-
57-
55-
142
421
81
166
135
61
230
228
72
65
48-
59
241
223
164
43-
39-
242
23
31
35
35-
324
30-
420
29-
204
158
317
202
25-
349
331
20-
332
216
218
4
15-
153
14-
A2 – 245 spots

## Slide 4
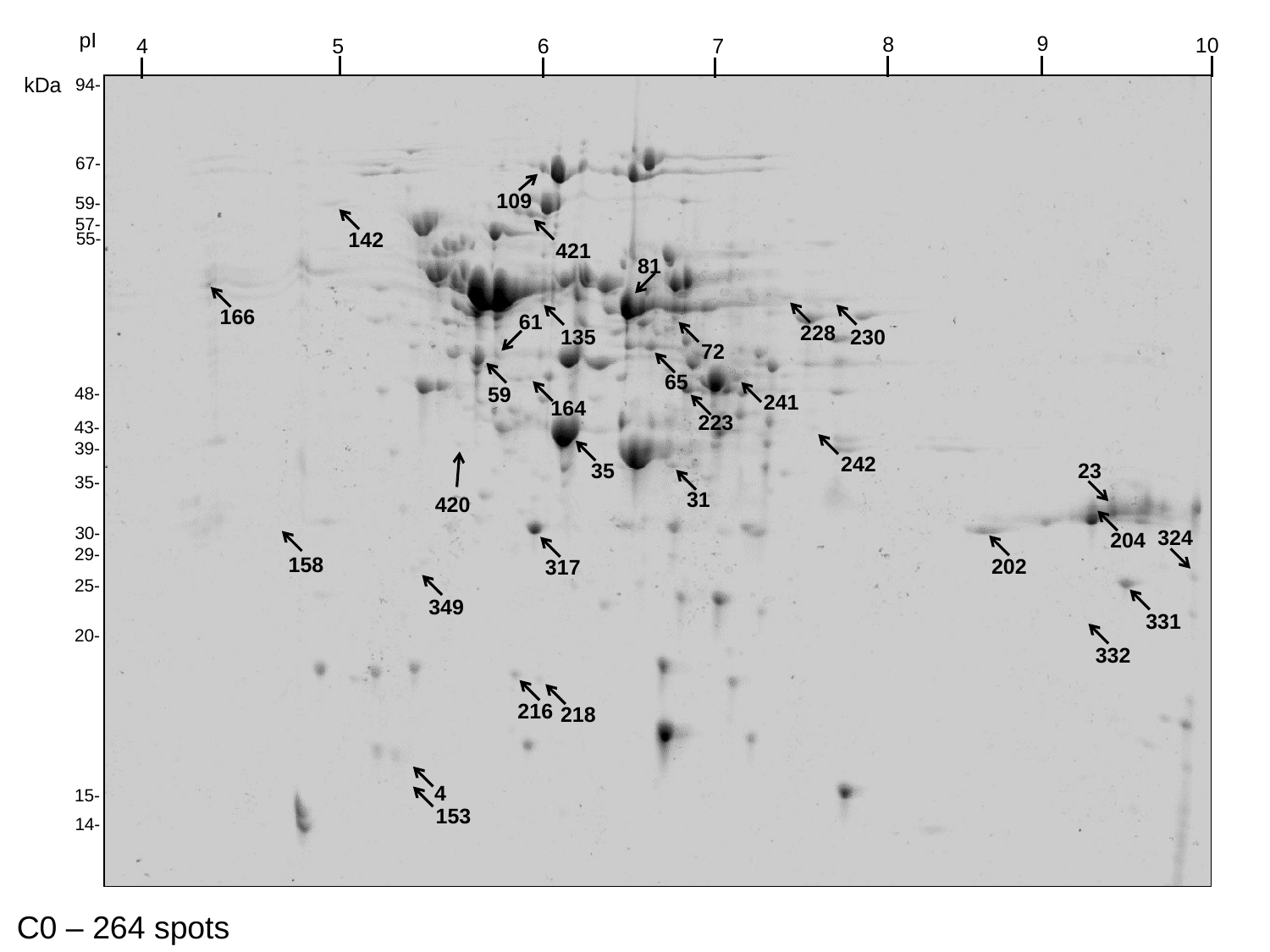

pI
9
8
10
7
5
4
6
kDa
94-
67-
109
59-
57-
142
55-
421
81
166
61
228
230
135
72
65
59
48-
241
164
223
43-
39-
242
35
23
35-
31
420
30-
324
204
29-
158
202
317
25-
349
331
20-
332
216
218
4
15-
153
14-
C0 – 264 spots

## Slide 5
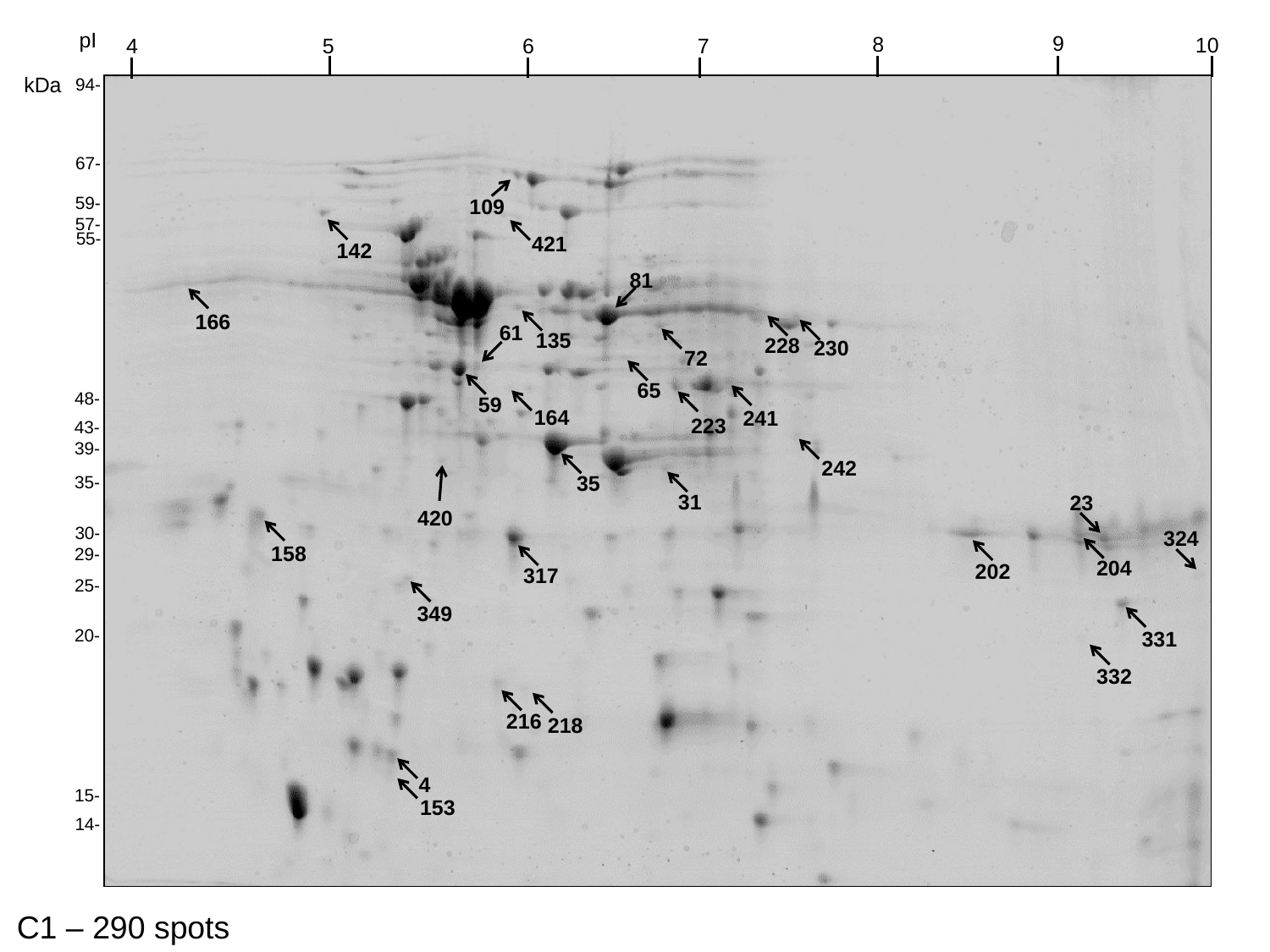

pI
9
8
10
7
5
4
6
kDa
94-
67-
59-
57-
109
55-
421
142
81
166
61
135
228
230
72
65
48-
59
164
241
223
43-
39-
242
35
35-
31
23
420
30-
324
158
29-
204
202
317
25-
349
20-
331
332
216
218
4
15-
153
14-
C1 – 290 spots

## Slide 6
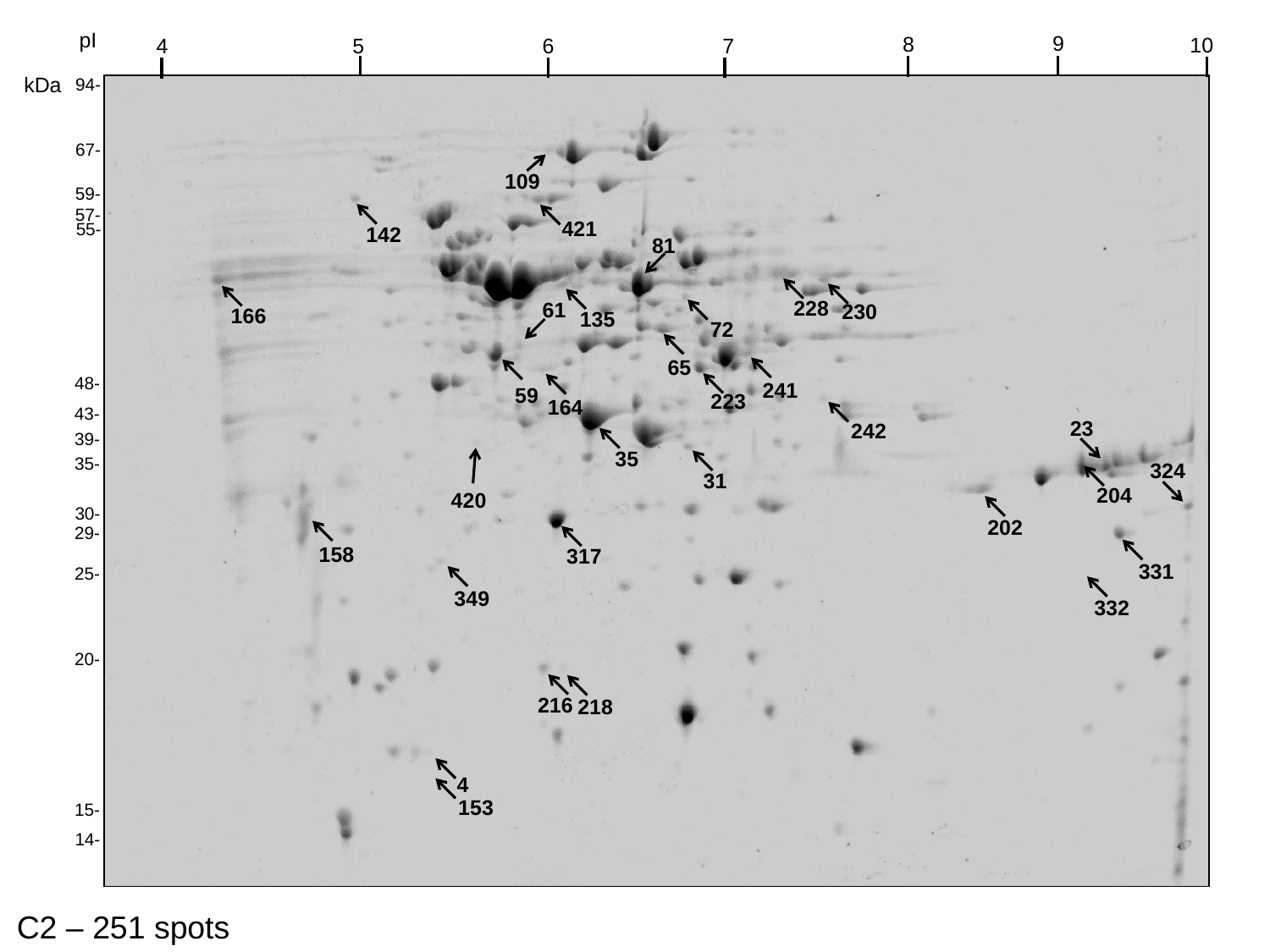

pI
9
8
10
7
5
4
6
kDa
94-
67-
109
59-
57-
421
55-
142
81
228
61
230
166
135
72
65
48-
241
59
223
164
43-
23
242
39-
35
35-
324
31
204
420
30-
202
29-
158
317
331
25-
349
332
20-
216
218
4
153
15-
14-
C2 – 251 spots

## Slide 7
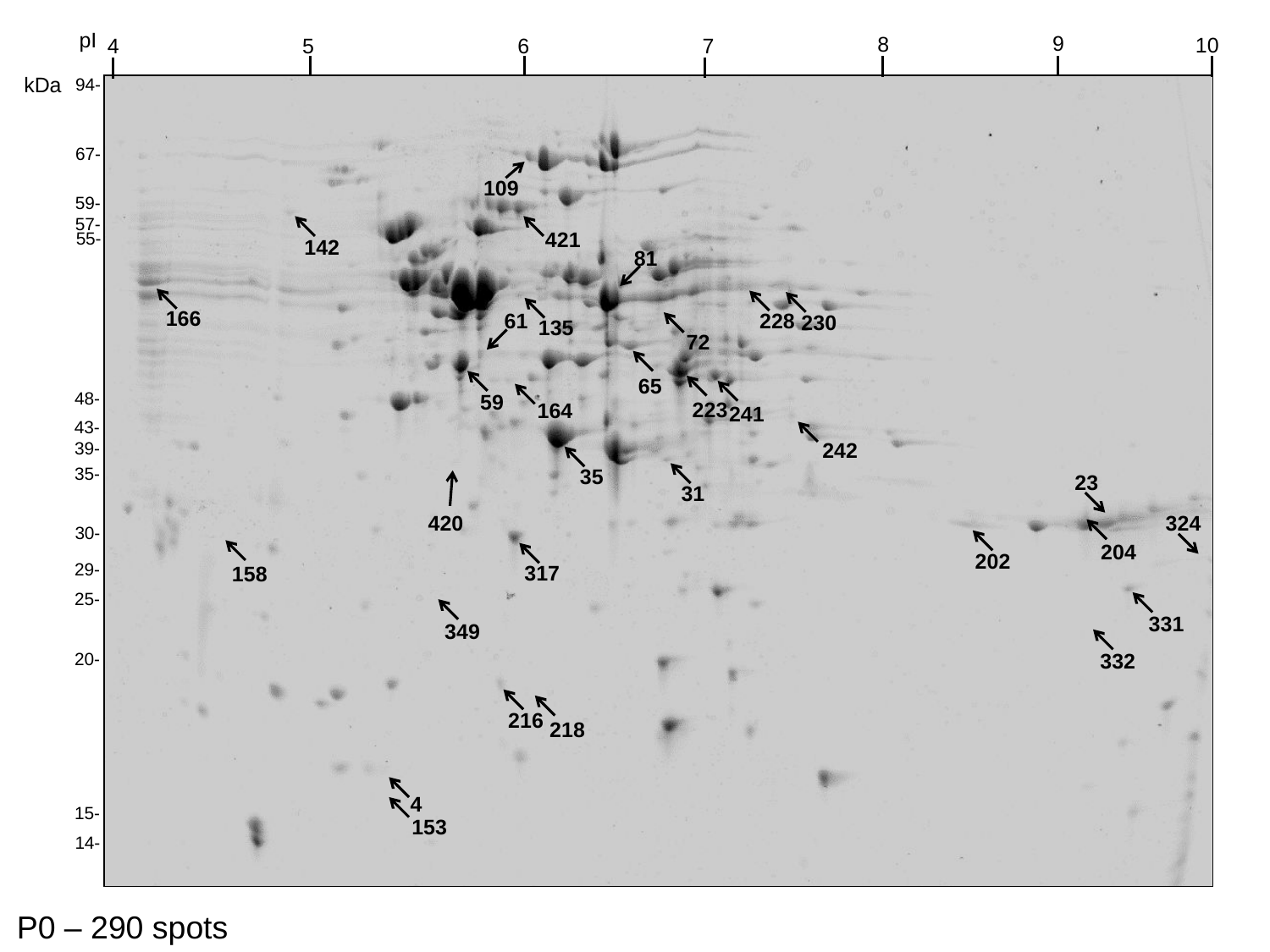

pI
9
8
10
7
5
4
6
kDa
94-
67-
109
59-
57-
421
55-
142
81
166
61
228
230
135
72
65
48-
59
223
164
241
43-
242
39-
35-
35
23
31
420
324
30-
204
202
29-
317
158
25-
331
349
332
20-
216
218
4
15-
153
14-
P0 – 290 spots

## Slide 8
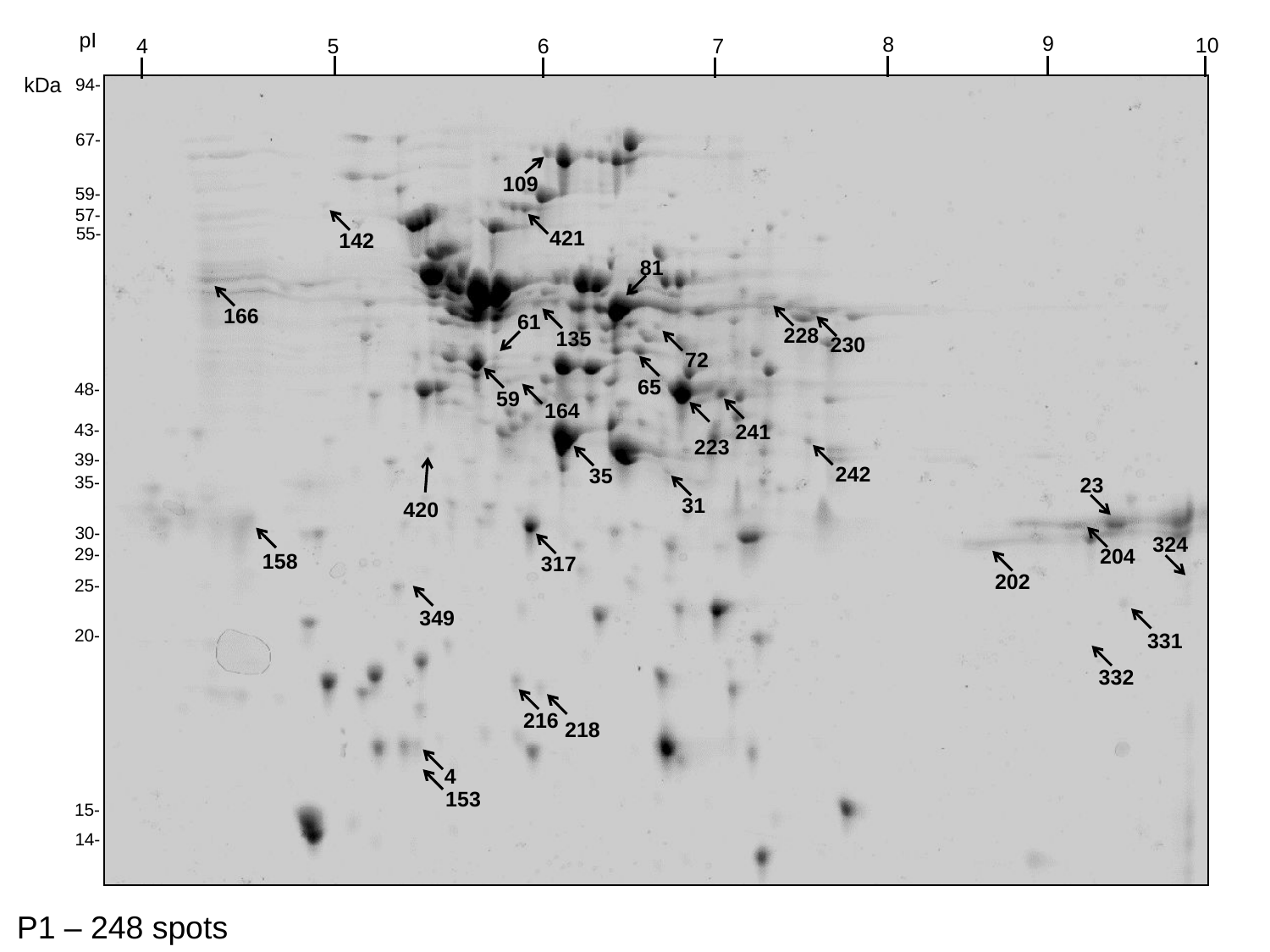

pI
9
8
10
7
5
4
6
kDa
94-
67-
109
59-
57-
55-
421
142
81
166
61
228
135
230
72
65
48-
59
164
43-
241
223
39-
242
35
35-
23
31
420
30-
324
204
29-
158
317
202
25-
349
20-
331
332
216
218
4
153
15-
14-
P1 – 248 spots

## Slide 9
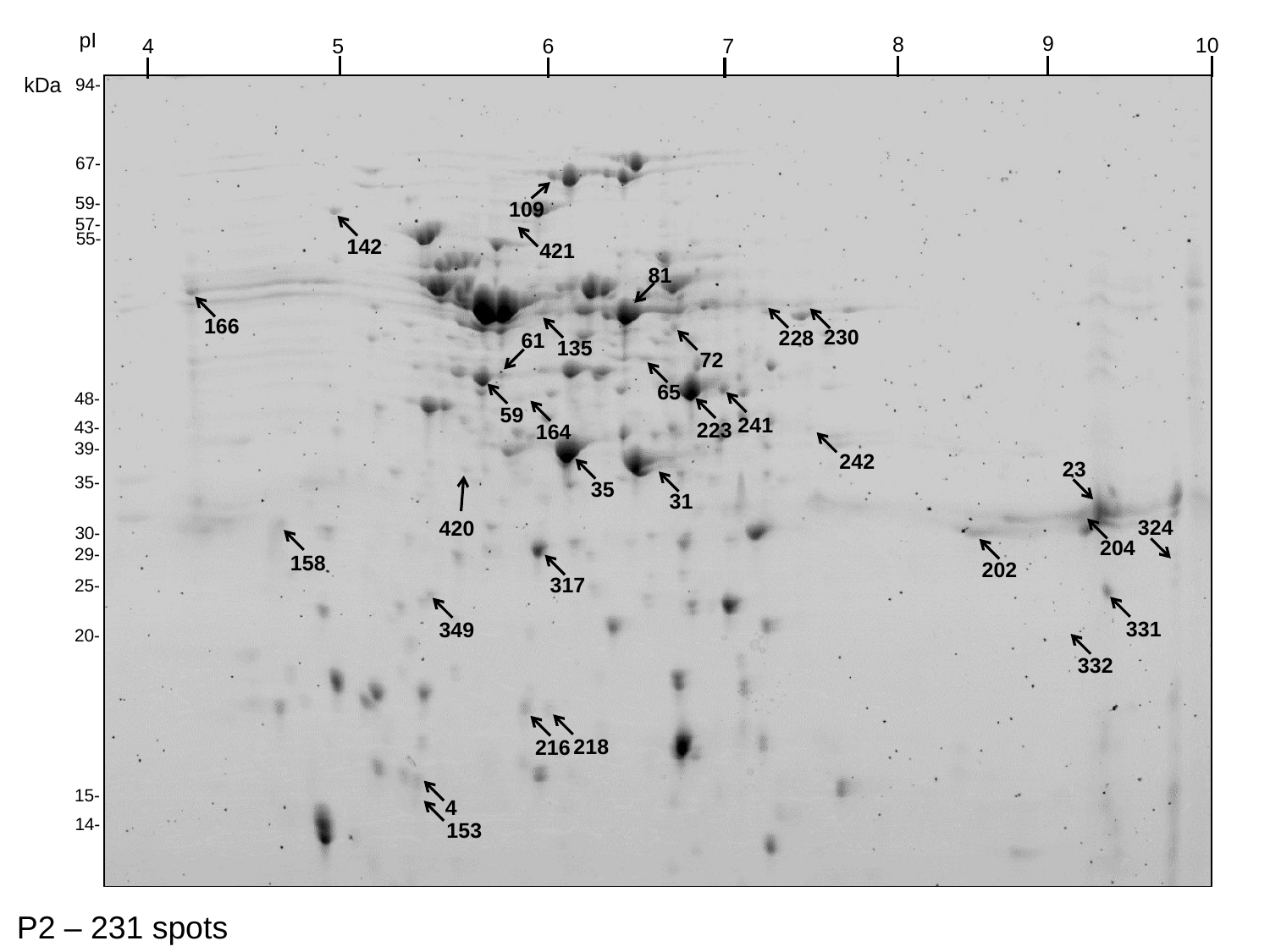

pI
9
8
10
7
5
4
6
kDa
94-
67-
59-
57-
109
55-
142
421
81
166
230
228
61
135
72
65
48-
59
241
43-
223
164
39-
242
23
35-
35
31
324
420
30-
204
29-
158
202
317
25-
331
349
20-
332
218
216
15-
4
14-
153
P2 – 231 spots

## Slide 10
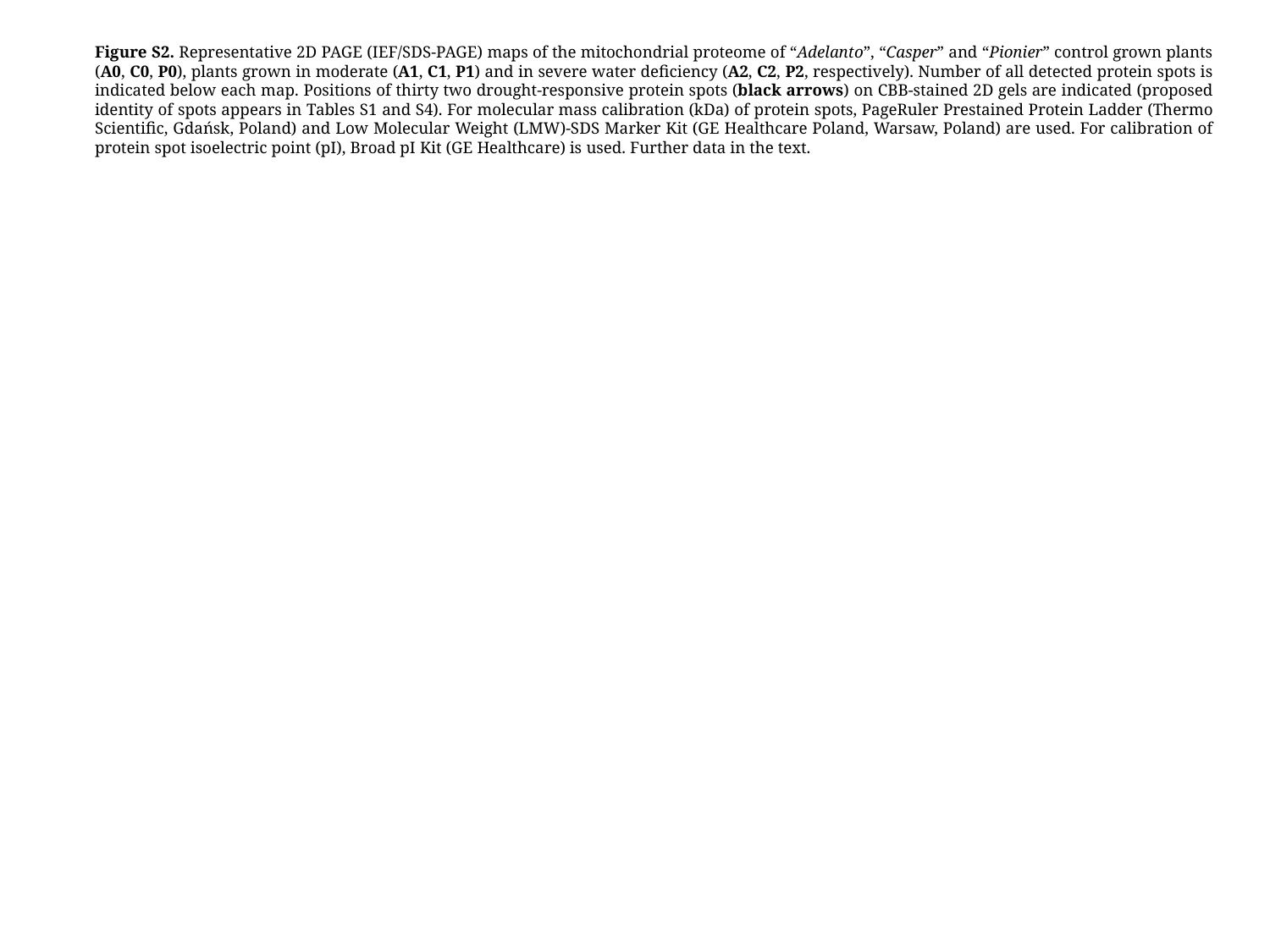

Figure S2. Representative 2D PAGE (IEF/SDS-PAGE) maps of the mitochondrial proteome of “Adelanto”, “Casper” and “Pionier” control grown plants (A0, C0, P0), plants grown in moderate (A1, C1, P1) and in severe water deficiency (A2, C2, P2, respectively). Number of all detected protein spots is indicated below each map. Positions of thirty two drought-responsive protein spots (black arrows) on CBB-stained 2D gels are indicated (proposed identity of spots appears in Tables S1 and S4). For molecular mass calibration (kDa) of protein spots, PageRuler Prestained Protein Ladder (Thermo Scientific, Gdańsk, Poland) and Low Molecular Weight (LMW)-SDS Marker Kit (GE Healthcare Poland, Warsaw, Poland) are used. For calibration of protein spot isoelectric point (pI), Broad pI Kit (GE Healthcare) is used. Further data in the text.
