# Supplementary material for: Mitochondrial Biogenesis in Diverse Cauliflower Cultivars under Mild and Severe Drought. Impaired Coordination of Selected Transcript and Proteomic Responses, and Regulation of Various Multifunctional Proteins
Source: Int J Mol Sci. 2018 Apr 10;19(4):1130. doi: 10.3390/ijms19041130 (PMC5979313; doi:10.3390/ijms19041130)
Supplement: Supplementary file 1 [file ijms-19-01130-s001.zip › Figure S3.pptx]

## Slide 1
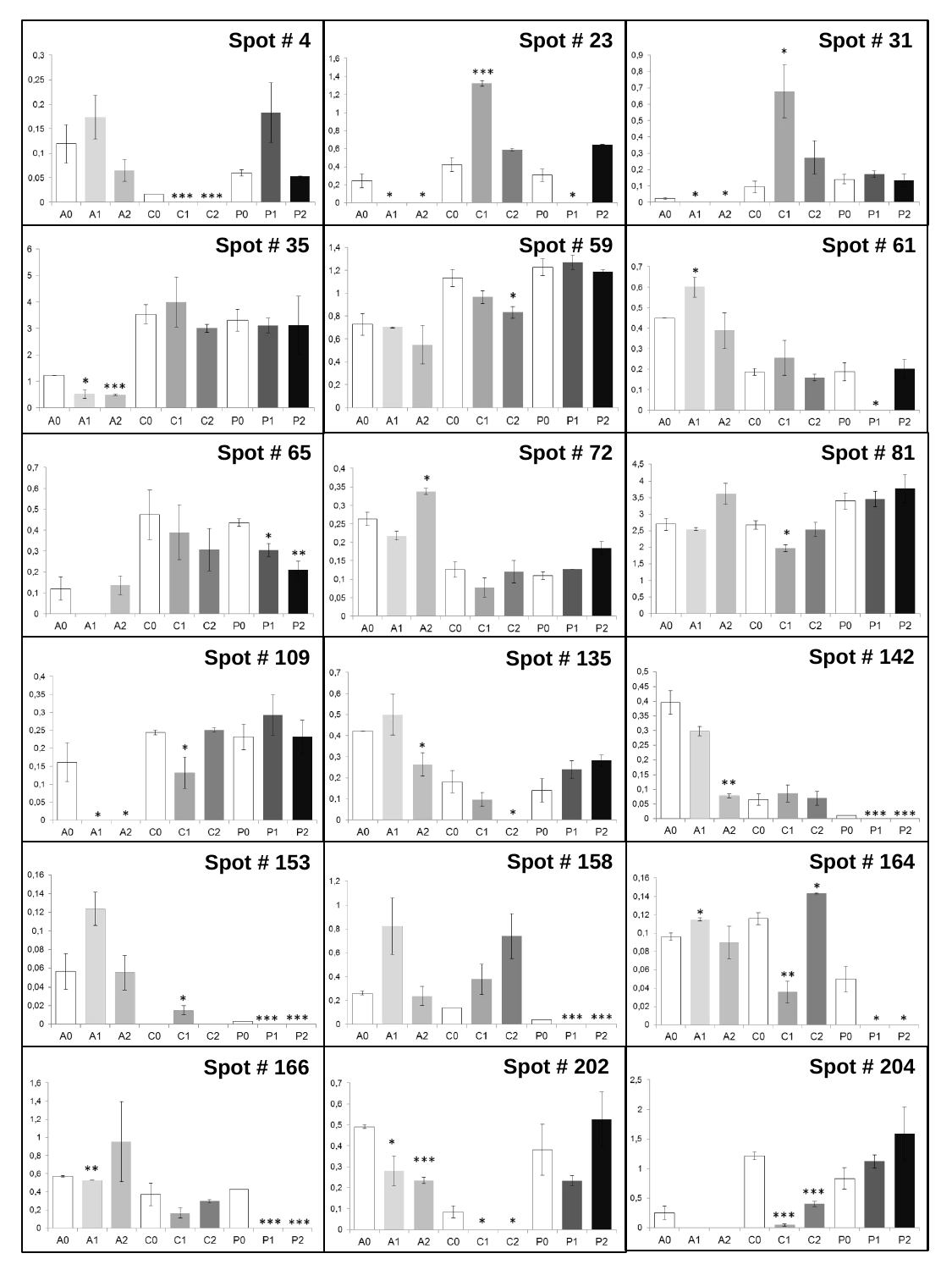

Spot # 31
Spot # 23
Spot # 4
Spot # 61
Spot # 59
Spot # 35
Spot # 65
Spot # 72
Spot # 81
Spot # 142
Spot # 109
Spot # 135
Spot # 164
Spot # 158
Spot # 153
Spot # 204
Spot # 202
Spot # 166

## Slide 2
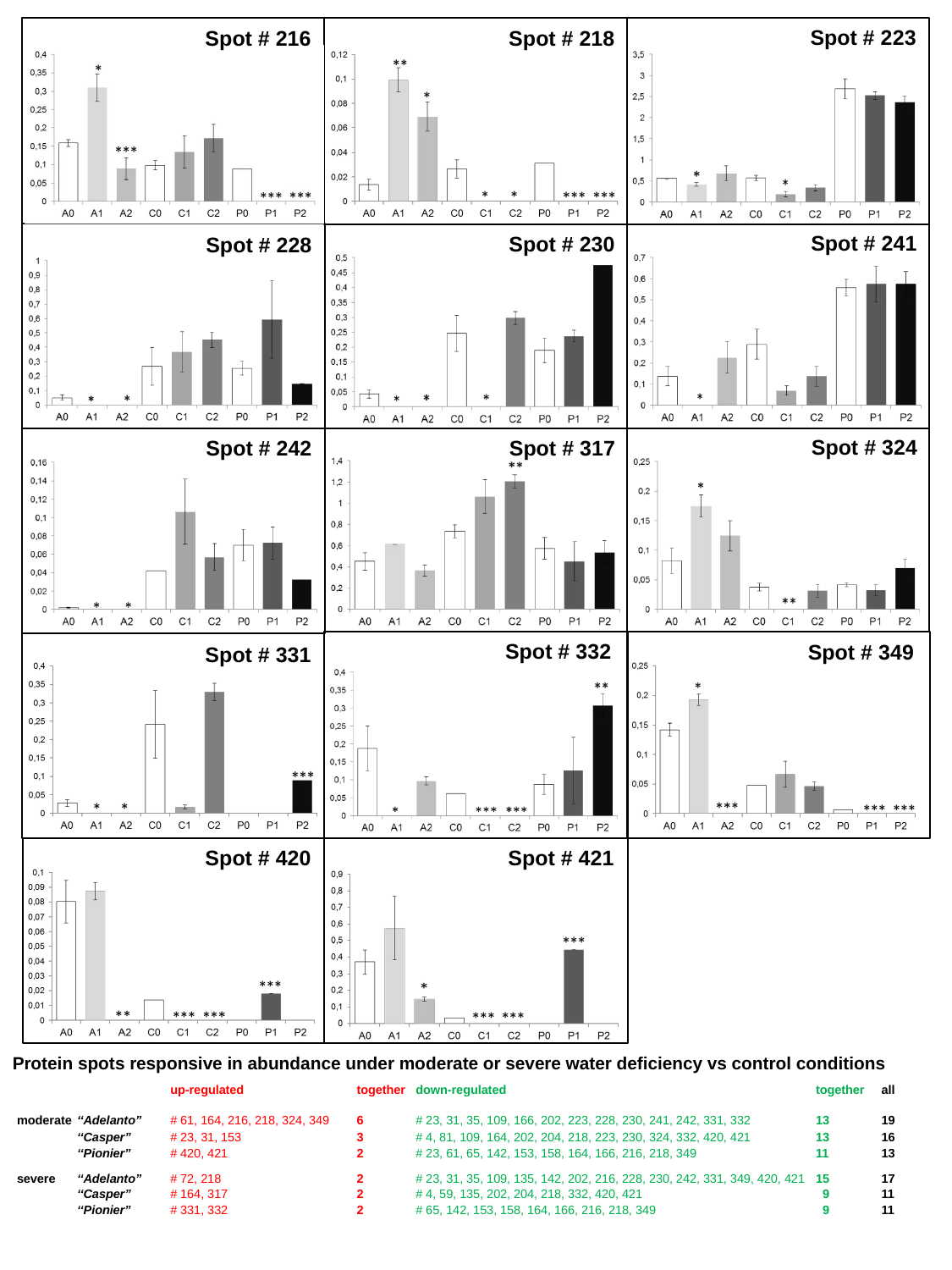

Spot # 223
Spot # 216
Spot # 218
Spot # 241
Spot # 230
Spot # 228
Spot # 324
Spot # 242
Spot # 317
Spot # 332
Spot # 349
Spot # 331
Spot # 420
Spot # 421
Protein spots responsive in abundance under moderate or severe water deficiency vs control conditions
| | | up-regulated | together | down-regulated | together | all |
| --- | --- | --- | --- | --- | --- | --- |
| moderate | ‘‘Adelanto” | # 61, 164, 216, 218, 324, 349 | 6 | # 23, 31, 35, 109, 166, 202, 223, 228, 230, 241, 242, 331, 332 | 13 | 19 |
| | ‘‘Casper” | # 23, 31, 153 | 3 | # 4, 81, 109, 164, 202, 204, 218, 223, 230, 324, 332, 420, 421 | 13 | 16 |
| | ‘‘Pionier” | # 420, 421 | 2 | # 23, 61, 65, 142, 153, 158, 164, 166, 216, 218, 349 | 11 | 13 |
| severe | ‘‘Adelanto” | # 72, 218 | 2 | # 23, 31, 35, 109, 135, 142, 202, 216, 228, 230, 242, 331, 349, 420, 421 | 15 | 17 |
| | ‘‘Casper” | # 164, 317 | 2 | # 4, 59, 135, 202, 204, 218, 332, 420, 421 | 9 | 11 |
| | ‘‘Pionier” | # 331, 332 | 2 | # 65, 142, 153, 158, 164, 166, 216, 218, 349 | 9 | 11 |

## Slide 3
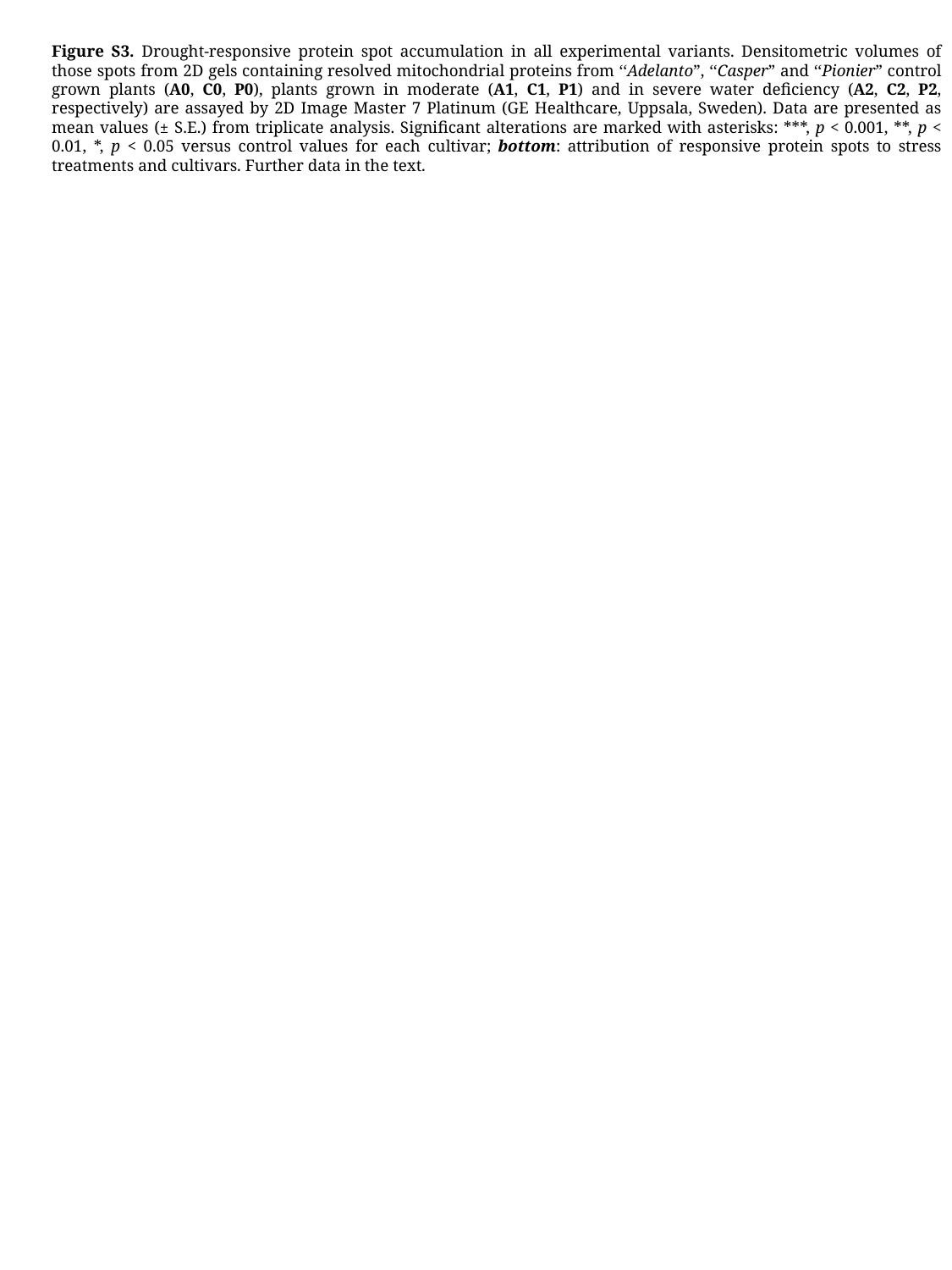

Figure S3. Drought-responsive protein spot accumulation in all experimental variants. Densitometric volumes of those spots from 2D gels containing resolved mitochondrial proteins from ‘‘Adelanto”, ‘‘Casper” and ‘‘Pionier” control grown plants (A0, C0, P0), plants grown in moderate (A1, C1, P1) and in severe water deficiency (A2, C2, P2, respectively) are assayed by 2D Image Master 7 Platinum (GE Healthcare, Uppsala, Sweden). Data are presented as mean values (± S.E.) from triplicate analysis. Significant alterations are marked with asterisks: ***, p < 0.001, **, p < 0.01, *, p < 0.05 versus control values for each cultivar; bottom: attribution of responsive protein spots to stress treatments and cultivars. Further data in the text.
