# Supplementary material for: Mitochondrial Biogenesis in Diverse Cauliflower Cultivars under Mild and Severe Drought. Impaired Coordination of Selected Transcript and Proteomic Responses, and Regulation of Various Multifunctional Proteins
Source: Int J Mol Sci. 2018 Apr 10;19(4):1130. doi: 10.3390/ijms19041130 (PMC5979313; doi:10.3390/ijms19041130)
Supplement: Supplementary file 1 [file ijms-19-01130-s001.zip › Table S7.doc]

**Table S7.** Primers used for RT-qPCR analyses.

| **Primer identifier** | **Primer sequence** | **Primer length** | **Primer T*_m_* [^o^C]** | **Primer positions and accession.version identifiers**  **of respective templates** | | **Amplicon description** |
| --- | --- | --- | --- | --- | --- | --- |
| AOX1a-F1 | TATGAGCGGTTGGATCTGGACT | 22-mer | 59.3 | 1-22 | KC631778.1 | *AOX1a* (alternative oxidase isoform 1a) |
| AOX1a-R2 | TTCCCTCCCTTGTTACCTCC | 20-mer | 58.3 | 165-146 |  |  |
| PRODH-F2 | ACCATCGAAGCAGCAAAATCC | 21-mer | 57.7 | 656-676 | XM_013743860.1 | *PRODH* (mitochondrial proline dehydrogenase 1) |
| PRODH-R1 | AGGGCTTGAATCGGAGAAAACC | 22-mer | 59.5 | 841-820 |  |  |
| P5CDH-F2 | CTTCAAACTACAACACGCTTCTG | 23-mer | 55.4 | 336-358 | XM_013773859.1 | *P5CDH* (mitochondrial Δ-1-pyrroline-5-carboxylate dehydrogenase 12A1) |
| P5CDH-R2 | AACTCCCGATTCCTCCACTTC | 21-mer | 58.5 | 415-395 |  |  |
| ERD14-F1 | CACCTATTTTCTCACTCGCC | 20-mer | 54.8 | 58-77 | XM_013737126.1 | *ERD14* (dehydrin) |
| ERD14-R2 | ACTATGTTTCTTCTCTTCTCCTTCT | 24-mer | 53.9 | 394-370 |  |  |
| ERD14L-F2 | AGGTTCATATCTCAGAGCCAGT | 22-mer | 56.2 | 116-137 | XM_013760369.1 | *ERD14-like* (dehydrin) |
| ERD14L-R1 | TCTCCTTCAGCAGTAGTAGCAG | 22-mer | 57.2 | 305-284 |  |  |
| CBF1a-F1 | ACGAGTCTCCTGCATTAAGC | 20-mer | 56.2 | 242-261 | XM_013770359.1 | *CBF1a* (transcription factor) |
| CBF1a-R1 | ATCTCGGCGGTTAGGAAAGTA | 21-mer | 57.1 | 449-429 |  |  |
| CBF1b-F1 | CGGGTAGGAAGAAGTTTCG | 19-mer | 54.4 | 263-281 | XM_013747430.1 | *CBF1b* (transcription factor) |
| CBF1b-R1 | TCTTCCACGGAGAGCTAAGG | 20-mer | 58.0 | 450-431 |  |  |
| CBF2-F1 | TGAGTCTCCGGTTACATTAGG | 21-mer | 54.9 | 190-210 | XM_013739789.1 | *CBF2* (transcription factor) |
| CBF2-R1 | AGGAAAGTACCGAGCCAAATC | 21-mer | 56.4 | 387-367 |  |  |
| CBF4-F1 | GTCTCCTGTTTCTGACAGTAG | 21-mer | 53.4 | 137-157 | XM_013753471.1 | *CBF4* (transcription factor) |
| CBF4-R1 | CCTAACCAAATCCTCGACTTC | 21-mer | 54.5 | 322- 302 |  |  |
| ACT1-F1 | AAAATGGCTGAGGCTGATGAC | 21-mer | 57.1 | 1-21 | KC631780.1 | *ACT1* (actin 1) |
| ACT1-R2 | ATCCCAACCATGACACCGTG | 20-mer | 59.0 | 149-130 |  |  |

Primer sequences are given in 5’🡪3’ orientation. The term ‘template’ refers to the accession used for primer design with their nucleotide coordinates indicating primer position. F, forward (upper) primer; R, reverse (lower) primer; T*_m_*, melting temperature calculated according to ThermoScientific T*_m_* Calculator at [www.thermofisher.com](../../Downloads/www.thermofisher.com) and expressed in Celsius degrees (^o^C).
