# Supplementary material for: Mitochondrial Biogenesis in Diverse Cauliflower Cultivars under Mild and Severe Drought. Impaired Coordination of Selected Transcript and Proteomic Responses, and Regulation of Various Multifunctional Proteins
Source: Int J Mol Sci. 2018 Apr 10;19(4):1130. doi: 10.3390/ijms19041130 (PMC5979313; doi:10.3390/ijms19041130)
Supplement: Supplementary file 1 [file ijms-19-01130-s001.zip › Table S1.doc]

**Table S1.** Variations in abundance of cauliflower mitochondrial protein spots and their proposed identities.

| **ID no. ^a^** | **Ratio of mean volumes of stress variants/ control variants ^b^** | | | | | | **MW exp.** | **pI exp.** | **Record no. ^c^** | **Proposed assignment; Species ^d^** | **accession.version identifiers** | **Ath homolog** | **MW theor.** | **pI theor.** | **Score** | **emPAI** | **Coverage [%]** | **U/T ^e^** |
| --- | --- | --- | --- | --- | --- | --- | --- | --- | --- | --- | --- | --- | --- | --- | --- | --- | --- | --- |
|  | **A1** | **A2** | **C1** | **C2** | **P1** | **P2** |  |  |  |  |  |  |  |  |  |  |  |  |
| 4 |  |  | LA^f^  *** | LA  *** |  |  | 16 | 5.41 | 1 | **reactive intermediate deaminase A, chloroplastic; *Bol*** | XP_013638176.1 | AT3G20390 | 19.984 | 8.52 | 993 | 2.12 | 57 | 9(3)/29(12) |
|  |  |  |  |  |  |  |  |  | 4 | **succinate dehydrogenase subunit 5, mitochondrial-like; *Bna*** | XP_013701701.1 | AT1G47420 | 26.216 | 8.29 | 613 | 2.32 | 31 | 6(3)/12(7) |
|  |  |  |  |  |  |  |  |  | 5 | **monothiol glutaredoxin-S15, mitochondrial-like; *Bra*** | XP_009115348.1 | AT3G15660 | 18.576 | 6.08 | 539 | 3.50 | 47 | 5(3)/11(7) |
|  |  |  |  |  |  |  |  |  | 6 | NADH dehydrogenase [ubiquinone] 1 alpha subcomplex subunit 12; *Bra* | XP_009119053.1 | AT3G03100 | 18.409 | 9.38 | 406 | 2.24 | 33 | 0(0)/7(7) |
|  |  |  |  |  |  |  |  |  | 8 | uncharacterized protein At2g27730, mitochondrial-like, copper ion binding protein; *Bol* | XP_013633838.1 | AT2G27730 | 12.111 | 9.43 | 352 | 1.72 | 40 | 2(2)/4(4) |
| 23 | LA  * | LA  * | 3.14  *** |  | LA  * |  | 33 | 9.62 | 1 | **mitochondrial outer membrane protein porin 2-like; *Bna*** | XP_013712390.1 | AT5G67500 | 29.539 | 9.01 | 1159 | 8.42 | 44 | 2(1)/30(14) |
|  |  |  |  |  |  |  |  |  | 3 | succinate dehydrogenase [ubiquinone] iron-sulfur subunit 1, mitochondrial; *Bra* | XP_009111569.1 | AT3G27380 | 31.970 | 8.83 | 926 | 6.22 | 43 | 1(1)/26(12) |
|  |  |  |  |  |  |  |  |  | 5 | **adenosine nucleotide translocator; *Bol*** | CAG17934.1 | AT5G13490 | 35.113 | 9.71 | 765 | 2.54 | 29 | 0(0)/14(10) |
|  |  |  |  |  |  |  |  |  | 8 | mitochondrial outer membrane protein porin 3; *Bol* | XP_013616085.1 | AT5G15090 | 29.440 | 8.44 | 636 | 1.62 | 32 | 5(3)/10(8) |
| 31 | LA  * | LA  * | 7.18  * |  |  |  | 38 | 6.93 | 1 | 3-hydroxyisobutyrate dehydrogenase, mitochondrial; *Bol* | XP_013625603.1 | AT4G20930 | 38.151 | 8.45 | 838 | 3.11 | 32 | 1(1)/21(11) |
|  |  |  |  |  |  |  |  |  | 3 | **α/β hydrolase domain-containing protein 11; *Bol*** | XP_013602016.1 | AT3G52570 | 37.124 | 6.52 | 797 | 1.79 | 37 | 14(11)/  14(11) |
|  |  |  |  |  |  |  |  |  | 4 | momilactone A synthase; *Bol* | XP_013613370.1 | AT3G29100 | 31.955 | 7.57 | 764 | 1.60 | 30 | 10(9)/10(9) |
|  |  |  |  |  |  |  |  |  | 6 | **γ carbonic anhydrase 2, mitochondrial; *Bol*** | XP_013599787.1 | AT1G47260 | 30.058 | 6.07 | 603 | 1.57 | 30 | 2(1)/9(8) |
|  |  |  |  |  |  |  |  |  | 7 | malate dehydrogenase 1, mitochondrial-like; *Bol* | XP_013587918.1 | AT1G53240 | 36.010 | 8.48 | 589 | 1.02 | 24 | 4(4)/8(8) |
| 35 | -2.38  * | -2.52  *** |  |  |  |  | 40 | 6.4 | 1 | **MDH_glyoxysomal_mitochondrial; *Bna*** | CDY26002.1 | AT1G53240 | 36.143 | 8.23 | 1865 | 20.47 | 67 | 0(0)/148(18) |
|  |  |  |  |  |  |  |  |  | 7 | **UPF0160 protein-like; *Bna*** | XP_013661846.1 | AT5G41970 | 42.637 | 5.97 | 1416 | 8.39 | 48 | 8(7)/30(20) |
|  |  |  |  |  |  |  |  |  | 13 | bifunctional L-3-cyanoalanine synthase/cysteine synthase C1, mitochondrial; *Bol* | XP_013631344.1 | AT3G61440 | 40.137 | 7.68 | 526 | 0.89 | 20 | 3(3)/8(7) |
| 59 |  |  |  | -1.36  * |  |  | 44 | 5.92 | 1 | **succinyl-CoA ligase [ADP-forming] subunit β, mitochondrial-like isoform X1; *Bna*** | XP_013747772.1 | AT2G20420 | 45.580 | 6.57 | 3291 | 69.90 | 73 | 0(0)/197(39) |
|  |  |  |  |  |  |  |  |  | 6 | multiple organellar RNA editing factor 1, mitochondrial-like; *Bol* | XP_013597851.1 | AT4G20020 | 41.654 | 8.50 | 1165 | 3.27 | 33 | 38(11)/  38(11) |
|  |  |  |  |  |  |  |  |  | 13 | **3-hydroxyisobutyryl-CoA hydrolase-like protein 3, mitochondrial; *Bol*** | XP_013637117.1 | AT4G13360 | 46.176 | 6.23 | 894 | 1.29 | 31 | 10(9)/12(11) |
| 61 | 1.33  * |  |  |  | LA  * |  | 44 | 6.05 | 4 | **elongation factor E1 (EF-Tu), mitochondrial; *Bol*** | AFL69959.1 | AT4G02930 | 49.414 | 6.11 | 942 | 1.32 | 25 | 2(2)/13(11) |
|  |  |  |  |  |  |  |  |  | 8 | **dihydropyrimidine dehydrogenase [NADP(+)], chloroplastic-like; *Bol*** | XP_013584617.1 | AT3G17810 | 47.553 | 6.05 | 817 | 1.24 | 27 | 1(1)/12(11) |
|  |  |  |  |  |  |  |  |  | 9 | **acetylornithine aminotransferase; *Bna*** | XP_013727002.1 | AT1G80600 | 48.408 | 6.15 | 808 | 0.81 | 22 | 1(1)/12(10) |
|  |  |  |  |  |  |  |  |  | 10 | dihydrolipoamide dehydrogenase 1, mitochondrial; *Ath* | NP_175237.1 | AT1G48030 | 54.239 | 6.96 | 782 | 0.92 | 22 | 0(0)/11(10) |
| 65 |  |  |  |  | -1.43  * | -2.07  ** | 45 | 6.76 | 1 | dihydrolipoyllysine-residue succinyltransferase component of 2-oxoglutarate dehydrogenase complex 1, mitochondrial-like isoform X1; *Bna* | XP_013685866.1 | AT5G55070 | 50.178 | 9.12 | 1258 | 3.05 | 39 | 1(1)/47(17) |
|  |  |  |  |  |  |  |  |  | 3 | **ATPase subunit 1; *Bna*** | YP_717155.1 | ATMG01190 | 55.382 | 6.01 | 1189 | 2.17 | 29 | 3(2)/22(17) |
|  |  |  |  |  |  |  |  |  | 6 | **glutamate dehydrogenase 1, mitochondrial; *Bol*** | XP_013605964.1 | AT5G18170 | 44.881 | 6.23 | 807 | 1.18 | 25 | 0(0)/12(10) |
|  |  |  |  |  |  |  |  |  | 7 | **cysteine desulfurase 1, mitochondrial; *Bra*** | XP_009112196.1 | AT5G65720 | 50.972 | 6.96 | 769 | 0.99 | 28 | 7(7)/11(11) |
| 72 |  | 1.28  * |  |  |  |  | 48 | 6.92 | 1 | **alanine-glyoxylate aminotransferase 2 homolog 2, mitochondrial isoform X1; *Bol*** | XP_013630039.1 | AT2G38400 | 52.064 | 6.87 | 1760 | 5.70 | 56 | 29(13)/  41(22) |
|  |  |  |  |  |  |  |  |  | 2 | **cysteine desulfurase 1, mitochondrial; *Bra*** | XP_009112196.1 | AT5G65720 | 50.972 | 6.96 | 1109 | 2.50 | 36 | 9(7)/20(15) |
|  |  |  |  |  |  |  |  |  | 3 | ATPase subunit 1; *Bna* | YP_717155.1 | ATMG01190 | 55.382 | 6.01 | 1080 | 1.67 | 26 | 3(3)/19(16) |
|  |  |  |  |  |  |  |  |  | 4 | aldehyde dehydrogenase family 2 member B7, mitochondrial isoform X1; *Bol* | XP_013604865.1 | AT1G23800 | 62.039 | 8.07 | 843 | 0.86 | 20 | 5(5)/12(12) |
|  |  |  |  |  |  |  |  |  | 5 | **dihydrolipoyl dehydrogenase 2, mitochondrial; *Bol*** | XP_013586991.1 | AT3G17240 | 54.083 | 6.93 | 751 | 0.81 | 22 | 1(1)/10(10) |
| 81 |  |  | -1.36  * |  |  |  | 48 | 6.68 | 1 | **ATPase subunit 1; *Bna*** | YP_717155.1 | ATMG01190 | 55.382 | 6.01 | 3015 | 44.18 | 67 | 3 (1)/  260(34) |
|  |  |  |  |  |  |  |  |  | 32 | succinate-semialdehyde dehydrogenase, mitochondrial; *Bol* | XP_013588031.1 | AT1G79440 | 57.111 | 7.51 | 898 | 1.07 | 25 | 13(12)/  13(12) |
|  |  |  |  |  |  |  |  |  | 33 | aldehyde dehydrogenase family 2 member B7; *Bra* | XP_009110081.1 | AT1G23800 | 58.895 | 6.84 | 805 | 0.72 | 18 | 5(4)/10(9) |
| 109 | LA  * | LA  * | -1.84  * |  |  |  | 64 | 6.29 | 1 | **aconitate hydratase 2, mitochondrial; *Bol*** | XP_013628812.1 | AT2G05710 | 109.012 | 6.58 | 2282 | 2.46 | 31 | 9(1)/61(28) |
| 135 |  | -1.60  * |  | LA  * |  |  | 48 | 6.27 | 2 | **chloroplast biotin carboxylase; *Bna*** | ADI79332.1 | AT5G35360 | 58.882 | 6.49 | 1646 | 2.90 | 44 | 0(0)/30(20) |
|  |  |  |  |  |  |  |  |  | 4 | **leucine aminopeptidase 3, chloroplastic; *Bna*** | XP_013730759.1 | AT4G30920 | 61.290 | 6.49 | 1249 | 1.43 | 29 | 7(6)/18(13) |
|  |  |  |  |  |  |  |  |  | 10 | **mitochondrial F1 ATP synthase β subunit; *Ath*** | CAC81058.1 | AT5G08670 | 63.560 | 6.52 | 814 | 0.83 | 21 | 5(4)/13(11) |
|  |  |  |  |  |  |  |  |  | 11 | aldehyde dehydrogenase family 2 member B4, mitochondrial; *Bol* | XP_013604322.1 | AT3G48000 | 58.787 | 7.60 | 807 | 0.72 | 21 | 6(6)/10(10) |
|  |  |  |  |  |  |  |  |  | 13 | succinate-semialdehyde dehydrogenase, mitochondrial; *Bol* | XP_013588031.1 | AT1G79440 | 57.111 | 7.51 | 733 | 0.71 | 17 | 10(9)/10(9) |
| 142 |  | -5.10  ** |  |  | LA  *** | LA  *** | 57 | 5.12 | 1 | **chloroplast Hsp70; *Esa*** | XP_006413448.1 | AT4G24280 | 76.275 | 5.18 | 2941 | 6.24 | 48 | 0(0)/74(36) |
|  |  |  |  |  |  |  |  |  | 2 | heat shock 70 kDa protein 6, chloroplastic-like; *Bra* | XP_009137440.1 | AT4G24280 | 76.078 | 5.32 | 2880 | 5.98 | 47 | 0(0)/74(35) |
|  |  |  |  |  |  |  |  |  | 4 | **heat shock protein 70-1; *Bna*** | AHL64247.1 | AT4G24280 | 75.697 | 5.19 | 2580 | 4.91 | 41 | 2(1)/69(32) |
| 153 |  |  | 1.51  * |  | LA  *** | LA  *** | 16 | 5.39 | 5 | **SWIB/MDM2 domain superfamily protein; *Bna*** | CDY50651.1 | AT3G03590 | 15.741 | 10.24 | 199 | 1.18 | 24 | 4(4)/4(4) |
| 158 |  |  |  |  | LA  *** | LA  *** | 32 | 4.37 | 1 | **RNA-binding protein CP33, chloroplastic-like; *Bol*** | XP_013593189.1 | AT3G52380 | 34.760 | 4.89 | 659 | 1.49 | 28 | 4(3)/11(8) |
|  |  |  |  |  |  |  |  |  | 2 | **glycine cleavage system H protein 2, mitochondrial-like; *Bra*** | XP_009133011.1 | AT2G35120 | 17.189 | 4.91 | 641 | 13.75 | 32 | 8(2)/23(9) |
|  |  |  |  |  |  |  |  |  | 6 | 31 kDa ribonucleoprotein, chloroplastic; *Bol* | XP_013632147.1 | AT4G24770 | 33.291 | 4.83 | 461 | 1.35 | 21 | 2(1)/9(6) |
| 164 | 1.19  * |  | -3.27  ** | 1.24  * | LA  * | LA  * | 43 | 6.13 | 1 | **uncharacterized protein At1g18480-like, similar to calcineurin-like metallo-phosphoesterase superfamily protein; *Bol*** | XP_013600166.1 | AT1G18480 | 46.917 | 6.41 | 1392 | 4.10 | 47 | 13(7)/30(19) |
|  |  |  |  |  |  |  |  |  | 2 | putative transferase At4g12130, mitochondrial isoform X1, contains aminomethyltransferase folate-binding domain; *Bna* | XP_013684131.1 | AT4G12130 | 42.154 | 7.67 | 1368 | 2.34 | 46 | 5(2)/22(16) |
|  |  |  |  |  |  |  |  |  | 5 | 4-diphosphocytidyl-2-C-methyl-D-erythritol kinase, chloroplastic-like; *Bna* | XP_013723782.1 | AT2G26930 | 42.947 | 6.48 | 694 | 0.95 | 20 | 9(8)/9(8) |
|  |  |  |  |  |  |  |  |  | 6 | **uncharacterized protein LOC106306345, sucrase/ferredoxin-like; *Bol*** | XP_013598376.1 | AT3G27570 | 40.545 | 6.47 | 690 | 1.56 | 24 | 8(6)/12(9) |
| 166 | -1.08  ** |  |  |  | LA  *** | LA  *** | 50 | 4.45 | 1 | **F0F1 ATP synthase subunit β; *Bna*** | CDY44783.1 | AT5G08670 | 59.579 | 6.26 | 2179 | 8.54 | 46 | 7(5)/73(25) |
|  |  |  |  |  |  |  |  |  | 3 | aldehyde dehydrogenase family 2 member B7, mitochondrial isoform; *Bol* | XP_013604865.1 | AT1G23800 | 62.039 | 8.07 | 1462 | 2.28 | 33 | 3(3)/23(18) |
|  |  |  |  |  |  |  |  |  | 7 | **alanine aminotransferase 1, mitochondrial-like; *Bol*** | XP_013587030.1 | AT1G72330 | 60.162 | 6.25 | 939 | 1.47 | 24 | 7(6)/20(15) |
|  |  |  |  |  |  |  |  |  | 9 | MPPβ, insulinase (peptidase family M16) protein; *Bna* | CDY43262.1 | AT3G02090 | 58.858 | 6.23 | 893 | 0.92 | 22 | 1(1)/13(12) |
|  |  |  |  |  |  |  |  |  | 11 | **methylmalonate-semialdehyde dehydrogenase [acylating], ALDH6B2, mitochondrial; *Bol*** | XP_013612807.1 | AT2G14170 | 57.229 | 6.67 | 839 | 0.95 | 19 | 4(4)/13(10) |
| 202 | -1.75  * | -2.10  *** | LA  * | LA  * |  |  | 32 | 9.24 | 1 | **mitochondrial outer membrane protein porin 1; *Bna*** | XP_013684234.1 | AT3G01280 | 29.397 | 8.48 | 1372 | 8.50 | 64 | 18(7)/68(16) |
|  |  |  |  |  |  |  |  |  | 2 | mitochondrial outer membrane protein porin 1-like; *Bol* | XP_013637592.1 | AT3G01280 | 29.265 | 8.57 | 877 | 3.05 | 38 | 0(0)/39(11) |
|  |  |  |  |  |  |  |  |  | 5 | **mitochondrial outer membrane protein porin 3; *Bol*** | XP_013616085.1 | AT5G15090 | 29.440 | 8.44 | 753 | 2.62 | 53 | 7(5)/13(10) |
|  |  |  |  |  |  |  |  |  | 6 | **adenosine nucleotide translocator; *Bol*** | CAG17934.1 | AT5G13490 | 35.113 | 9.71 | 704 | 1.70 | 26 | 0(0)/11(10) |
|  |  |  |  |  |  |  |  |  | 7 | **NADH-cytochrome b_5_ reductase-like protein; *Bol*** | XP_013622739.1 | AT5G20080 | 35.974 | 9.15 | 691 | 1.64 | 33 | 11(9)/11(9) |
|  |  |  |  |  |  |  |  |  | 9 | **mitochondrial dicarboxylate/tricarboxylate transporter DTC-like; *Bna*** | XP_013667347.1 | AT5G19760 | 32.342 | 9.35 | 642 | 1.18 | 29 | 2(2)/8(8) |
|  |  |  |  |  |  |  |  |  | 10 | adenylate kinase (ADK); *Aal* | KFK26681.1 | AT5G50370 | 27.505 | 7.64 | 594 | 1.80 | 29 | 1(1)/10(8) |
|  |  |  |  |  |  |  |  |  | 12 | uncharacterized protein LOC106444193; *Bna* | XP_013741151.1 | AT4G26410 | 29.091 | 8.59 | 564 | 1.38 | 32 | 8(8)/8(8) |
| 204 |  |  | -25.87  *** | -3.02  *** |  |  | 33 | 9.51 | 1 | **mitochondrial outer membrane protein porin 1; *Bna*** | XP_013684234.1 | AT3G01280 | 29.397 | 8.48 | 1580 | 19.11 | 61 | 33(9)/57(16) |
|  |  |  |  |  |  |  |  |  | 2 | mitochondrial outer membrane protein porin 1-like; *Bol* | XP_013637592.1 | AT3G01280 | 29.265 | 8.57 | 1533 | 15.40 | 60 | 7(1)/68(15) |
|  |  |  |  |  |  |  |  |  | 5 | **mitochondrial outer membrane protein porin 3; *Bol*** | XP_013616085.1 | AT5G15090 | 29.440 | 8.44 | 1160 | 7.53 | 59 | 22(7)/32(13) |
|  |  |  |  |  |  |  |  |  | 6 | **NADH-cytochrome b_5_ reductase-like protein; *Bol*** | XP_013622739.1 | AT5G20080 | 35.974 | 9.15 | 1150 | 5.95 | 61 | 19(14)/  22(15) |
|  |  |  |  |  |  |  |  |  | 7 | mitochondrial outer membrane protein porin 3-like; *Bra* | NP_001288856.1 | AT5G15090 | 29.389 | 8.73 | 978 | 7.53 | 51 | 13(5)/25(12) |
|  |  |  |  |  |  |  |  |  | 9 | **mitochondrial dicarboxylate/tricarboxylate transporter DTC-like; *Bra*** | XP_009120859.1 | AT5G19760 | 32.110 | 9.43 | 790 | 1.92 | 35 | 2(1)/11(9) |
|  |  |  |  |  |  |  |  |  | 14 | **putative enoyl-CoA hydratase/isomerase YngF; *Bna*** | XP_013707823.1 | AT4G16800 | 33.103 | 9.12 | 576 | 2.15 | 27 | 12(7)/12(7) |
|  |  |  |  |  |  |  |  |  | 16 | succinate dehydrogenase [ubiquinone] iron-sulfur subunit 1, mitochondrial-like; *Bna* | XP_013670878.1 | AT3G27380 | 31.732 | 8.66 | 553 | 1.99 | 34 | 2(2)/11(8) |
| 216 | 1.94  * | -1.79  *** |  |  | LA  *** | LA  *** | 19 | 6.08 | 1 | **uncharacterized protein At3g15000, mitochondrial-like; *Bra*** | XP_009143687.1 | AT3G15000 | 26.763 | 9.06 | 922 | 8.32 | 44 | 10(4)/25(11) |
|  |  |  |  |  |  |  |  |  | 4 | **plastid developmental protein DAG, chloroplastic-like; *Tha*** | XP_010538368.1 | AT1G32580 | 26.048 | 9.26 | 344 | 1.06 | 22 | 2(1)/10(5) |
| 218 | 7.35  ** | 5.11  * | LA  * | LA  * | LA  *** | LA  *** | 18 | 6.20 | 1 | **multiple organellar RNA editing factor 6, mitochondrial-like; *Bna*** | XP_013744657.1 | AT2G35240 | 26.255 | 9.30 | 913 | 5.04 | 40 | 3(2)/16(12) |
|  |  |  |  |  |  |  |  |  | 3 | **cysteine desulfurase 1, mitochondrial; *Bra*** | XP_009112196.1 | AT5G65720 | 50.972 | 6.96 | 356 | 0.37 | 11 | 2(2)/5(5) |
|  |  |  |  |  |  |  |  |  | 4 | **hypothetical protein AMTR_s00067p00179200 contains peptidase inhibitor I9 region; *Atr*** | ERN18921.1 | AT2G33430 | 21.522 | 5.56 | 273 | 1.06 | 16 | 1(1)/5(4) |
| 223 | -1.32  * |  | -2.97  * |  |  |  | 44 | 6.99 | 1 | **pyruvate dehydrogenase E1 component subunit α-1, mitochondrial; *Bra*** | NP_001288895.1 | AT1G59900 | 43.488 | 7.59 | 2150 | 43.93 | 55 | 4(2)/78(28) |
|  |  |  |  |  |  |  |  |  | 7 | isovaleryl-CoA dehydrogenase, mitochondrial; *Bna* | XP_013642511.1 | AT3G45300 | 45.367 | 8.38 | 886 | 1.24 | 31 | 9(7)/13(10) |
|  |  |  |  |  |  |  |  |  | 10 | **fructose-bisphosphate aldolase, cytoplasmic isozyme-like; *Bol*** | XP_013637326.1 | AT3G52930 | 38.809 | 6.28 | 783 | 1.67 | 34 | 6(4)/12(10) |
|  |  |  |  |  |  |  |  |  | 13 | formate dehydrogenase, mitochondrial; *Bra* | XP_009121632.1 | AT5G14780 | 42.584 | 8.13 | 665 | 1.27 | 25 | 2(1)/12(10) |
|  |  |  |  |  |  |  |  |  | 14 | isocitrate dehydrogenase [NAD] regulatory subunit 1, mitochondrial; *Bna* | XP_013611345.1 | AT4G35260 | 40.296 | 8.04 | 641 | 0.99 | 24 | 6(4)/11(8) |
| 228 | LA  * | LA  * |  |  |  |  | 49 | 7.99 | 1 | **serine hydroxymethyltransferase 1, mitochondrial isoform X1; *Bol*** | XP_013629759.1 | AT4G37930 | 58.112 | 8.08 | 2075 | 5.49 | 56 | 3(2)/48(27) |
|  |  |  |  |  |  |  |  |  | 4 | serine hydroxymethyltransferase 2, mitochondrial; *Bol* | XP_013592155.1 | AT5G26780 | 57.289 | 8.57 | 1515 | 3.04 | 40 | 3(2)/31(21) |
|  |  |  |  |  |  |  |  |  | 8 | **aldehyde dehydrogenase family 2 member B7, mitochondrial isoform X1; *Bol*** | XP_013604865.1 | AT1G23800 | 62.039 | 8.07 | 1095 | 1.06 | 26 | 1(1)/14(14) |
|  |  |  |  |  |  |  |  |  | 14 | dihydrolipoyl dehydrogenase 2, mitochondrial; *Bra* | XP_009146028.1 | AT3G17240 | 53.837 | 6.93 | 824 | 0.92 | 23 | 5(5)/11(11) |
|  |  |  |  |  |  |  |  |  | 17 | **probable Xaa-Pro aminopeptidase 3; *Bol*** | XP_013604203.1 | AT1G09300 | 53.675 | 7.23 | 774 | 1.30 | 20 | 13(9)/15(11) |
| 230 | LA  * | LA  * | LA  * |  |  |  | 49 | 8.57 | 1 | ATPase subunit 1; *Bna* | YP_717155.1 | ATMG01190 | 55.382 | 6.01 | 2446 | 20.33 | 48 | 17(7)/64(33) |
|  |  |  |  |  |  |  |  |  | 5 | **serine hydroxymethyltransferase 2, mitochondrial-like isoform X1; *Bol*** | XP_013592395.1 | AT5G26780 | 56.739 | 8.70 | 1178 | 2.66 | 31 | 1(1)/23(17) |
|  |  |  |  |  |  |  |  |  | 6 | catalase-1; *Bra* | XP_009103333.1 | AT1G20630 | 57.281 | 6.83 | 1144 | 2.61 | 40 | 5(3)/26(17) |
|  |  |  |  |  |  |  |  |  | 10 | **fumarate hydratase 1, mitochondrial-like; *Bna*** | XP_013676489.1 | AT2G47510 | 52.954 | 8.65 | 1062 | 1.79 | 31 | 3(3)/18(14) |
|  |  |  |  |  |  |  |  |  | 15 | **mitochondrial import inner membrane translocase subunit TIM44-2-like; *Bna*** | XP_013675966.1 | AT2G36070 | 50.601 | 8.89 | 789 | 1.21 | 25 | 12(10)/  13(11) |
|  |  |  |  |  |  |  |  |  | 16 | **succinate-semialdehyde dehydrogenase, mitochondrial; *Bol*** | XP_013588031.1 | AT1G79440 | 57.111 | 7.51 | 727 | 1.12 | 20 | 13(10)/  14(11) |
|  |  |  |  |  |  |  |  |  | 17 | catalase-2-like; *Bna* | XP_013723778.1 | AT4G35090 | 57.242 | 6.75 | 673 | 1.07 | 20 | 0(0)/15(10) |
| 241 | LA  * |  |  |  |  |  | 43 | 7.48 | 1 | **alcohol dehydrogenase 1B; *Bra*** | XP_009150414.1 | AT5G63620 | 46.938 | 7.57 | 1857 | 9.01 | 53 | 0(0)/70(21) |
|  |  |  |  |  |  |  |  |  | 3 | **pyruvate dehydrogenase E1 component subunit α-1, mitochondrial; *Bra*** | NP_001288895.1 | AT1G59900 | 43.488 | 7.59 | 1813 | 11.95 | 58 | 1(1)/41(24) |
|  |  |  |  |  |  |  |  |  | 6 | formate dehydrogenase, mitochondrial; *Bra* | XP_009121632.1 | AT5G14780 | 42.584 | 8.13 | 1296 | 2.83 | 52 | 10(8)/22(17) |
|  |  |  |  |  |  |  |  |  | 7 | phosphoserine aminotransferase 1, chloroplastic; *Bol* | XP_013630270.1 | AT4G35630 | 47.686 | 8.58 | 1036 | 1.91 | 33 | 1(1)/16(13) |
|  |  |  |  |  |  |  |  |  | 8 | **isocitrate dehydrogenase [NAD] regulatory subunit 1, mitochondrial; *Bol*** | XP_013611345.1 | AT4G35260 | 40.296 | 8.04 | 963 | 2.98 | 33 | 5(3)/26(12) |
|  |  |  |  |  |  |  |  |  | 9 | aminomethyltransferase, mitochondrial; *Bol* | XP_013605123.1 | AT1G11860 | 44.753 | 8.82 | 896 | 1.52 | 31 | 0(0)/13(11) |
|  |  |  |  |  |  |  |  |  | 13 | pyruvate dehydrogenase (acetyl-transferring) kinase, mitochondrial; *Bol* | XP_013621899.1 | AT3G06483 | 41.809 | 6.43 | 742 | 1.31 | 37 | 4(3)/11(10) |
|  |  |  |  |  |  |  |  |  | 15 | bifunctional L-3-cyanoalanine synthase/cysteine synthase C1, mitochondrial; *Bol* | XP_013604593.1 | AT3G61440 | 40.000 | 8.84 | 704 | 1.81 | 27 | 12(8)/14(9) |
| 242 | LA  * | LA  * |  |  |  |  | 41 | 8.46 | 1 | **stomatin-like protein 2, mitochondrial; *Bol*** | XP_013625752.1 | AT4G27585 | 39.096 | 9.02 | 1615 | 13.63 | 48 | 6(4)/69(19) |
|  |  |  |  |  |  |  |  |  | 3 | **electron transfer flavoprotein subunit α, mitochondrial isoform X1; *Bra*** | XP_009107213.1 | AT1G50940 | 38.001 | 8.54 | 659 | 1.31 | 25 | 10(8)/11(9) |
|  |  |  |  |  |  |  |  |  | 5 | **bifunctional L-3-cyanoalanine synthase/cysteine synthase C1, mitochondrial; *Bol*** | XP_013604593.1 | AT3G61440 | 40.000 | 8.84 | 600 | 1.60 | 21 | 2(1)/12(8) |
| 317 |  |  |  | 1.64  ** |  |  | 30 | 6.18 | 1 | **probable ATP synthase 24 kDa subunit, mitochondrial; *Bol*** | XP_013633568.1 | AT2G21870 | 27.639 | 8.45 | 1861 | 187.14 | 58 | 0(0)/134(22) |
| 324 | 2.12  * |  | LA  ** |  |  |  | 25 | 9.71 | 1 | **copper ion binding protein, contains glutamyl-tRNA(Gln) amidotransferase, subunit E region; *Bna*** | CDY46359.1 | AT4G05400 | 27.797 | 9.70 | 926 | 6.70 | 35 | 2(2)/21(11) |
|  |  |  |  |  |  |  |  |  | 3 | **outer envelope pore protein 24B, chloroplastic; *Bol*** | XP_013614074.1 | AT5G42960 | 23.698 | 9.23 | 650 | 2.76 | 41 | 1(1)/11(9) |
| 331 | LA  * | LA  * |  |  |  | 8.86  *** | 26 | 9.56 | 1 | **single-stranded DNA-binding protein WHY2, mitochondrial isoform X1; *Bol*** | XP_013590037.1 | AT1G71260 | 26.660 | 9.48 | 1047 | 15.97 | 46 | 56(10)/  70(14) |
|  |  |  |  |  |  |  |  |  | 2 | **NADH-cytochrome b_5_ reductase-like protein; *Bol*** | XP_013622739.1 | AT5G20080 | 35.974 | 9.15 | 464 | 0.85 | 23 | 7(7)/7(7) |
|  |  |  |  |  |  |  |  |  | 3 | mimitin, mitochondrial-like isoform X1; *Bol* | XP_013617304.1 | AT4G26965 | 20.680 | 8.96 | 377 | 1.47 | 28 | 7(5)/7(5) |
| 332 | LA  * |  | LA  *** | LA  *** |  | 3.51  ** | 24 | 9.53 | 1 | **NADH-cytochrome b_5_ reductase-like protein; *Bol*** | XP_013622739.1 | AT5G20080 | 35.974 | 9.15 | 1003 | 3.48 | 42 | 24(11)/  27(13) |
|  |  |  |  |  |  |  |  |  | 2 | **ribosome recycling factor (RRF), chloroplast; *Bna*** | CDX93926.1 | AT3G63190 | 29.686 | 9.37 | 587 | 1.11 | 29 | 6(6)/8(8) |
|  |  |  |  |  |  |  |  |  | 3 | **single-strand DNA-binding protein, OB-fold-like protein, mitochondrial; *Ath*** | NP_188488.1 | AT3G18580 | 24.256 | 9.29 | 481 | 1.46 | 33 | 7(7)/7(7) |
| 349 | 1.35  * | LA  *** |  |  | LA  *** | LA  *** | 26 | 5.53 | 1 | **20 kDa chaperonin, chloroplastic; *Bra*** | XP_009131811.1 | AT5G20720 | 26.801 | 7.77 | 1425 | 17.87 | 67 | 13(6)/39(16) |
|  |  |  |  |  |  |  |  |  | 2 | **50S ribosomal protein L21, mitochondrial-like; *Bna*** | XP_013671504.1 | AT4G30930 | 29.113 | 6.14 | 633 | 1.96 | 29 | 0(0)/14(8) |
| 420 |  | LA  ** | LA  *** | LA  *** | 1.79  *** |  | 38 | 5.91 | 1 | **cysteine synthase, chloroplastic/ chromoplastic; *Bra*** | XP_009142968.1 | AT2G43750 | 42.426 | 7.55 | 1222 | 3.84 | 43 | 3(1)/27(15) |
|  |  |  |  |  |  |  |  |  | 3 | **fructose-bisphosphate aldolase 2, chloroplastic; *Bol*** | XP_013605252.1 | AT4G38970 | 43.106 | 6.78 | 963 | 1.43 | 30 | 1(1)/12(11) |
|  |  |  |  |  |  |  |  |  | 4 | **malonyl-CoA-acyl carrier protein transacylase, mitochondrial-like; *Bra*** | XP_009144320.1 | AT2G30200 | 40.918 | 8.83 | 902 | 1.94 | 27 | 8(6)/14(10) |
|  |  |  |  |  |  |  |  |  | 6 | **cysteine synthase, mitochondrial; *Bra*** | XP_009138900.1 | AT3G59760 | 45.323 | 8.70 | 824 | 1.50 | 30 | 2(2)/13(11) |
| 421 |  | -2.52  * | LA  *** | LA  *** | 44.52  *** |  | 56 | 6.29 | 1 | **heat shock 70 kDa protein 9, mitochondrial; *Bol*** | XP_013629229.1 | AT4G37910 | 73.256 | 5.90 | 2384 | 3.84 | 39 | 0(0)/39(30) |
|  |  |  |  |  |  |  |  |  | 2 | heat shock 70 kDa protein 9, mitochondrial-like; *Bol* | XP_013595853.1 | AT4G37910 | 73.188 | 5.73 | 2110 | 2.90 | 36 | 2(2)/33(26) |
|  |  |  |  |  |  |  |  |  | 3 | **heat shock 70 kDa protein 10, mitochondrial; *Bol*** | XP_013629357.1 | AT5G09590 | 72.964 | 5.75 | 1727 | 2.01 | 28 | 0(0)/26(19) |
|  |  |  |  |  |  |  |  |  | 4 | heat shock 70 kDa protein 10, mitochondrial-like; *Bna* | XP_013726845.1 | AT5G09590 | 72.938 | 5.55 | 1693 | 2.01 | 28 | 1(1)/26(19) |
|  |  |  |  |  |  |  |  |  | 6 | **heat shock 70 kDa protein, mitochondrial-like; *Gma*** | XP_003529268.1 | AT5G09590 | 72.596 | 5.82 | 919 | 0.94 | 18 | 0(0)/17(12) |

Records for most abundant proteins (with respect to Exponentially Modified Protein Abundance Index, emPAI) are shown. For each spot, the nominal (theor.) and the observed (exp.) isoelectric point (pI) as well as molecular mass (MW in kD) values are indicated. ^a^ Spot ID as the index in the reference gel (see Figure 1); ^b^ The ratio of mean normalized volumes for stressed/control variants (1- drought 1. level (the moderate drought); 2- drought 2. level (the severe drought) in each investigated cultivar (**A**- *‘‘Adelanto”*, **C**- *‘‘Casper”*, **P**- *‘‘Pionier”*)). Only significant in *p*-test fold change values are shown (down-regulations are highlighted in green, up-regulations- in red), more data on Figure S3; **p*<0.05, ***p*<0.01, ****p*<0.001; ^c^ Record number- for each spot, all records for the proposed protein assignments (from the LC-MS/MS protein prediction list) that fit to the experimental data are included (top-fit assignments are **bolded**); ^d^ Species abbreviations: *Aal*- *Arabis alpina*; *Ath*- *Arabidopsis thaliana*; *Atr*- *Amborella trichopoda*; *Bna*- *Brassica napus*; *Bol*- *Brassica oleracea*; *Bra*- *Brassica rapa*; *Esa*- *Eutrema salsugineum*; *Gma*- *Glycine max*; *Tha*- *Tarenaya hassleriana*. ^e^ U/T- the number of unique to all peptides for the each protein spot. In brackets- the number of non-redundant sequences within unique/total peptides number. ^f^ LA- low-abundant protein (with the % volume < 0.01) indicating a strong down-regulation. Detailed peptide data are shown in Table S4; no.- number
